# Risk of Mild Cognitive Impairment or Probable Dementia in New Users of Angiotensin II Receptor Blockers and Angiotensin-Converting Enzyme Inhibitors: A Secondary Analysis of Data From the Systolic Blood Pressure Intervention Trial (SPRINT)

**DOI:** 10.1001/jamanetworkopen.2022.20680

**Published:** 2022-07-14

**Authors:** Jordana B. Cohen, Zachary A. Marcum, Chong Zhang, Catherine G. Derington, Tom H. Greene, Lama Ghazi, Jennifer S. Herrick, Jordan B. King, Alfred K. Cheung, Nick Bryan, Mark A. Supiano, Joshua A. Sonnen, William S. Weintraub, Daniel Scharfstein, Jeff Williamson, Nicholas M. Pajewski, Adam P. Bress

**Affiliations:** 1 Department of Medicine, Renal-Electrolyte and Hypertension Division, Perelman School of Medicine, University of Pennsylvania, Philadelphia; 2 Department of Biostatistics, Epidemiology, and Informatics, Perelman School of Medicine, University of Pennsylvania, Philadelphia; 3 Department of Pharmacy, University of Washington School of Pharmacy, Seattle; 4 Intermountain Healthcare Department of Population Health Sciences, Division of Biostatistics, Spencer Fox Eccles School of Medicine, University of Utah, Salt Lake City; 5 Intermountain Healthcare Department of Population Health Sciences, Division of Health System Innovation and Research, Spencer Fox Eccles School of Medicine, University of Utah, Salt Lake City; 6 Clinical and Translational Research Accelerator, Yale University School of Medicine, New Haven, Connecticut; 7 George E. Wahlen Department of Veterans Affairs Medical Center, Salt Lake City, Utah; 8 Institute for Health Research, Kaiser Permanente Colorado, Aurora; 9 Department of Internal Medicine, Spencer Fox Eccles School of Medicine, University of Utah, Salt Lake City; 10 Medical Service, Veterans Affairs Salt Lake City Healthcare System, Salt Lake City, Utah; 11 Department of Radiology, Perelman School of Medicine, University of Pennsylvania, Philadelphia; 12 Geriatrics Division, Spencer Fox Eccles School of Medicine, University of Utah, Salt Lake City; 13 Departments of Pathology, Neurology, and Neurosurgery, McGill University, Montréal, Québec, Canada; 14 MedStar Health Research Institute and Georgetown University, Washington, DC; 15 Sticht Center for Healthy Aging and Alzheimer’s Prevention, Wake Forest University School of Medicine, Winston-Salem, North Carolina; 16 Department of Biostatistics and Data Science, Wake Forest University School of Medicine, Winston-Salem, North Carolina

## Abstract

**Importance:**

The cardiovascular and renal outcomes of angiotensin-II receptor blocker (ARB) and angiotensin-converting enzyme inhibitor (ACEI) treatment are well-known; however, few studies have evaluated initiation of these agents and cognitive impairment.

**Objective:**

To emulate a target trial to evaluate the cognitive outcomes of initiating an ARB- vs ACEI-based antihypertensive regimen in individuals at risk for mild cognitive impairment (MCI) and probable dementia (PD).

**Design, Setting, and Participants:**

Active comparator, new-user observational cohort study design using data from the Systolic Blood Pressure Intervention Trial (SPRINT), conducted November 2010 through July 2018. Marginal cause-specific hazard ratios (HRs) and treatment-specific cumulative incidence functions were estimated with inverse probability (IP) weighting to account for confounding. Participants were using neither an ARB nor ACEI at baseline. Data analysis was conducted from April 7, 2021, to April 26, 2022.

**Exposures:**

New users of ARB vs ACEI during the first 12 months of trial follow-up.

**Main Outcomes and Measures:**

Composite of adjudicated amnestic MCI or PD.

**Results:**

Of 9361 participants, 727 and 1313 new users of an ARB or ACEI, respectively, with well-balanced baseline characteristics between medication exposure groups after inverse probability weighting (mean [SD] age, 67 [9.5] years; 1291 ]63%] male; 240 [33%] Black; 89 [12%] Hispanic; 383 [53%] White; and 15 [2%] other race or ethnicity. In the primary analysis, during a median follow-up of 4.9 years, the inverse probability–weighted rate of amnestic MCI or PD was 4.3 vs 4.6 per 100 person-years among participants initiating ARB vs ACEI (HR, 0.93; 95% CI, 0.76-1.13). In subgroup analyses, new users of an ARB vs ACEI had a lower rate of amnestic MCI or PD among those in the standard systolic blood pressure treatment arm (HR, 0.61; 95% CI, 0.41-0.91) but not in the intensive arm (HR, 1.17; 95% CI, 0.90-1.52) (*P* = .007 for interaction).

**Conclusions and Relevance:**

In this observational cohort study of US adults at high cardiovascular disease risk, there was no difference in the rate of amnestic MCI or PD among new users of an ARB compared with ACEI, although 95% CIs were wide.

## Key Points

**Question:**

Is initiation of angiotensin II receptor blockers (ARBs), compared with angiotensin-converting enzyme inhibitors (ACEIs), associated with a reduced risk of cognitive impairment, after controlling for potential pretreatment confounders?

**Findings:**

This active-comparator, new-user design cohort study identified 2040 new users of ACEIs/ARBs from the Systolic Blood Pressure Intervention Trial (SPRINT). The overall risk of cognitive impairment was not appreciably different between new users of an ARB vs ACEI.

**Meaning:**

The findings of this study suggest that pragmatic trials reflective of blood pressure management outside the clinical trial setting are warranted to further elucidate cognitive outcomes with ARBs vs ACEIs initiation.

## Teaser

This cohort study examines the rates of mild cognitive impairment and probable dementia among initiators of an angiotensin II receptor blocker (ARB) vs angiotensin-converting enzyme inhibitor (ACEI)–based antihypertensive medication regimen.

## Introduction

Hypertension is a leading modifiable risk factor for cognitive decline and dementia.^1,2^ A recent meta-analysis of randomized clinical trials (RCTs) reported that lowering blood pressure (BP) with antihypertensive medications reduces the risk of poor cognitive outcomes.^3^ Whether cognitive benefits are achieved via BP reduction alone or via direct effects of the antihypertensive medications on the brain independent of BP lowering remains unclear.^4^

Angiotensin II receptor blockers (ARBs) and angiotensin-converting enzyme inhibitors (ACEIs) are used by approximately 40 million adults in the US, and current BP guidelines recommend ARBs and ACEIs interchangeably.^5,6^Compared with ACEIs, ARBS may have beneficial effects on the brain. Angiotensin II receptor blockers directly antagonize the angiotensin II type 1 receptor, which is downstream in the renin-angiotensin system from where ACEIs act. By directly antagonizing angiotensin II type 1 receptors, ARBs shift circulating angiotensin II to bind to and stimulate angiotensin II type 2 and type 4 receptors. Stimulation of angiotensin II type 2 and angiotensin II type 4 receptors leads to reduced oxidative stress, neuroinflammation, and endothelial dysfunction, and improved cerebral hypoperfusion, potentially leading to memory-enhancing effects.^7,8,9^ In contrast, by inhibiting the conversion of angiotensin I to angiotensin II, ACEIs lower circulating angiotensin II and thereby reduce stimulation of angiotensin II type 1, as well as angiotensin II type 2 and angiotensin II type 4 receptor activity.

Although several large RCTs have compared the effects of ARBs vs ACEIs on cardiovascular and renal outcomes,^10^ fewer data are available for cognitive outcomes^11^ and, to our knowledge, none have evaluated adjudicated mild cognitive impairment (MCI), which is a transitional state to dementia. Using an active-comparator, new-user design as a secondary analysis of the Systolic Blood Pressure Intervention Trial (SPRINT), we sought to emulate a target trial^12^ to compare the risk of developing MCI and probable dementia (PD) between those initiating an ARB vs ACEI.

## Methods

### Study Design

We conducted an observational cohort study using a new-user, active comparator^13,14^ design as a secondary analysis of SPRINT to emulate a target trial.^12^ Eligibility criteria included SPRINT-eligible US adults not taking an ARB or ACEI at the baseline visit. New use of an ARB or an ACEI was ascertained between months 1 and 12 after SPRINT randomization. Participants were followed up from initiation date until occurrence of an outcome event or the end of SPRINT follow-up. SPRINT was approved by the institutional review board at each participating site, and each participant provided written informed consent. The present analysis was approved by the institutional review board at the University of Utah. This study followed the Strengthening the Reporting of Observational Studies in Epidemiology (STROBE) reporting guideline for cohort studies.

### The SPRINT Study Population

The SPRINT design and main results for cardiovascular disease and cognitive outcomes have been reported.^15,16,17^ Briefly, SPRINT was an RCT of intensive (target goal <120 mm Hg) vs standard (target goal <140 mm Hg) systolic BP (SBP) control in 9361 US adults aged 50 years or older with a high risk of cardiovascular disease and SBP 130 to 180 mm Hg. Key exclusion criteria in SPRINT included individuals with diabetes, history of stroke, heart failure, living in a nursing home, and with a diagnosis of dementia or receiving medications primarily for dementia (additional details in the eMethods in Supplement 1). After randomization, participants’ antihypertensive medications were adjusted using treatment algorithms (eFigure 1 in Supplement1) to achieve their target SBP goal.

### Identification of ARB or ACEI New Users in SPRINT

To identify participants who were ARB or ACEI new users (ie, initiators) in SPRINT, we restricted the analyses to SPRINT participants not taking an ARB or ACEI at the baseline visit (n = 3892) (eFigure 2 in Supplement 1). Next, we identified the first follow-up study visit in which an ARB or ACEI was initiated and measured the time in days from randomization to initiation (eFigure 3 in Supplement 1). Participants who did not initiate an ACEI or ARB or who initiated both on a single visit during the first 12 months of SPRINT follow-up were excluded. The month of initiation of an ARB or ACEI served as the index date and start of follow-up (eFigure 4 in Supplement 1). We chose a 12-month identification window to observe new ARB or ACEI use to provide enough time between the last initiation date and the first cognitive assessment during which the medication would plausibly have an observable effect on cognitive outcomes (ie, 1 year).

### Cognitive Outcome Ascertainment

In SPRINT, cognitive assessments were planned at baseline, 2 years, and 4 years of follow-up, and at study closeout (eFigure 5 in Supplement 1). Because the randomized intervention was stopped early at the recommendation of the data safety monitoring board due to high degree of efficacy of the intensive BP-lowering intervention, a final extended follow-up visit including cognitive assessment was conducted between October 2017 and July 2018. For this analysis, the final date of follow-up was July 22, 2018. Data analysis for the present study was performed from April 7, 2021, to April 26, 2022. In SPRINT, cognitive status was ascertained using a 3-step process. First, at each site, trained examiners administered in-person cognitive screening assessments to all participants at baseline and at 2 and 4 years of follow-up and at a close-out visit. The cognitive screening assessments included a test of global cognitive function (Montreal Cognitive Assessment [MoCA]; range, 0-30 [higher scores denote better cognitive function]), processing speed (Digit Symbol Coding Test of the Wechsler Adult Intelligence Scale; range, 0-135 [higher scores denote better cognitive function]), and learning and memory (Logical Memory forms I and II subtests of the Wechsler Memory Scale; ranges, 0-28 and 0-14 [higher scores denote better cognitive function]).^1,2,3^ Next, a preidentified proxy, the Functional Activities Questionnaire, was administered. This 10-item measure of functional abilities (range, 0-30 [higher scores indicating greater functional abilities])^4^ was used in the following situations: (1) White participants with a MoCA score less than 19 (with <12 years of education) or less than 21 (with ≥12 years of education), (2) participants of other races or ethnicities with a MoCA score less than 17 (with <12 years of education) or less than 19 (with ≥12 years of education), or (3) any participant with a decrease of 5 or more points from a previous MoCA assessment. Participants who scored greater than 0 on the Functional Activities Questionnaire or less than or equal to 1 on the 5-point Delayed Recall subtest of the MoCA underwent further testing (ie, an extended cognitive battery that measured attention and concentration, verbal and nonverbal memory, language, and executive functions (eTable 1 in Supplement 1). For participants who could not be assessed in person during follow-up, a validated telephone battery was administered.^5^ In this case, the Functional Activities Questionnaire was administered if the participant scored less than or equal to 31 on the Modified Telephone Interview for Cognitive Status.^6^ The Dementia Questionnaire was administered to a prespecified contact if a participant died or was unable to communicate by telephone. These data were reviewed by an expert panel masked to treatment assignment to adjudicate cognitive status. Participants were classified into 1 of 3 primary categories: no cognitive impairment, MCI, or probable dementia. Mild cognitive impairment was subtyped into amnestic vs nonamnestic MCI, using criteria adapted from Winblad et al.^7^ Unclassifiable cases were placed in a cannot classify category. Each case was reviewed independently by 2 SPRINT study adjudicators using standardized diagnostic criteria for probable dementia and MCI.^8,9^ Agreements by the 2 adjudicators were final. Disagreements were discussed by the full panel, with the final classification decision achieved by a majority vote. No subclassification of probable dementia was made. Additional details of the adjudication process can be found in the trial protocol (Supplement 2). More details of the SPRINT MIND cognitive outcome ascertainment and adjudication process can be found in the trial protocol (eMethods in Supplement 1).^16^

### Primary Outcome

The primary outcome was a composite of adjudicated amnestic MCI or PD. The definition of MCI in the original SPRINT protocol (ie, protocol-defined MCI) required 2 consecutive occurrences of amnestic or nonamnestic MCI. For the present analysis, because amnestic MCI has been shown to be consistently associated with an increased risk of progression to dementia,^18^ we chose to analyze time to amnestic MCI to avoid conditioning the outcome on future assessments and to capture as many MCI events as possible. Because adjudicated MCI status was not available on or before the index date, incident amnestic MCI could not be determined definitively.

### Secondary Outcomes

Secondary outcomes included PD or protocol-defined MCI, PD alone, amnestic MCI alone, and protocol-defined MCI alone. In addition, death was examined as a composite outcome with each of the secondary outcomes and separately. We also assessed serious adverse events (hypotension, syncope, bradycardia, electrolyte abnormality, acute kidney injury or failure, orthostatic hypotension with or without dizziness, and changes in serum potassium or sodium levels) (Supplement Protocol).

### Covariate Information

Preinitiation covariates were selected a priori based on their potential role as confounders (ie, variables that are associated with ARB and ACEI initiation and cognitive outcomes) (Table 1). For sociodemographic characteristics, comorbidities, cognitive function, and concomitant nonantihypertensive medication use variables, we used the prerandomization values because they were the only available measurements before the index date. Race and ethnicity was self-reported in SPRINT, and categories were collapsed in this cohort study for subgroup analysis because there were a small number of or no individuals in certain categories. Data on race and ethnicity were included because there are prior studies in SPRINT and other trials that have identified racial differences in cognitive and cardiac outcomes which merited assessment for effect modification, with appropriate adjustment for social determinants of health. The categories included Hispanic, non-Hispanic Black (hereafter Black), non-Hispanic White (hereafter White), and other (which included Asian, Hawaiian or Pacific Islander, Native American, and other race or ethnicity). The NIH, the funding organization, requires reporting of race and ethnicity. For BP and laboratory test results, which were measured more frequently, we used the most recent value before the index date. All covariates were measured before ARB or ACEI initiation.

**Table 1.** Baseline Characteristics of SPRINT Participants Included in the Current Analysis Before and After IP Weighting^a^

Characteristic	Before IP weighting			After IP weighting		
	No. (%)		ASMD	%		ASMD
	ARB new users (n = 727)	ACEI new users (n = 1313)		ARB new users	ACEI new users	
Demographic						
Age, mean (SD), y	67 (10)	67 (9)	0.01	67 (10)	67 (10)	0.00
Sex						
Male	419 (58)	872 (66)	0.18	36	36	0.01
Female	308 (42)	441 (34)		64	64	
Race and ethnicity						
Black	240 (33)	374 (28)	0.10	29	30	0.01
Hispanic	89 (12)	100 (8)	0.16	10	9	0.01
White	383 (53)	817 (62)	0.19	60	59	0.00
Other^b^	15 (2)	22 (2)	0.03	2	2	0.00
Social and behavioral						
Lives with others	501 (69)	888 (68)	0.03	69	68	0.01
Smoker						
Current	107 (15)	220 (17)	0.06	15	16	0.01
Former	271 (37)	550 (42)	0.09	40	40	0.01
Never	349 (48)	542 (41)	0.13	45	44	0.02
Educational level						
Less than high school	68 (9)	135 (10)	0.03	10	10	0.00
High school graduate only	96 (13)	231 (18)	0.12	16	16	0.01
Post high school graduate	260 (36)	446 (34)	0.04	35	35	0.01
College graduate or greater	303 (42)	501 (38)	0.07	39	39	0.00
Health insurance status						
Medicare	373 (51)	680 (52)	0.01	51	52	0.01
Medicaid	49 (7)	89 (7)	0.00	7	7	0.01
Veterans Affairs	88 (12)	270 (21)	0.22	17	18	0.03
Private	340 (47)	529 (40)	0.13	44	43	0.03
Usual source of care						
Physician office/outpatient clinic	601 (83)	1057 (81)	0.05	82	81	0.01
Community health care facility/other	82 (11)	146 (11)	0.01	11	11	0.02
None or missing	42 (6)	109 (8)	0.09	7	7	0.00
Medical history						
Clinical CVD	77 (11)	200 (15)	0.14	13	14	0.01
Atrial fibrillation	54 (7)	92 (7)	0.02	8	7	0.01
Depression	138 (19)	246 (19)	0.01	19	19	0.01
Baseline cognitive assessment, mean (SD)						
MoCA^c^	23 (4)	23 (4)	0.07	23 (4)	23 (4)	0.00
Logical Memory Form II^d^	8 (3)	8 (3)	0.08	8 (3)	8 (3)	0.00
Digit symbol coding test^e^	52 (16)	51 (15)	0.12	51 (15)	51 (15)	0.01
Clinical/laboratory measurements						
Blood pressure, mean (SD), mm Hg						
Systolic	144 (16)	142 (16)	0.09	143 (15)	143 (16)	0.00
Diastolic	80 (12)	79 (12)	0.07	80 (12)	80 (13)	0.00
Resting heart rate, bpm	67 (12)	68 (12)	0.05	67 (12)	67 (12)	0.00
BMI	30 (6)	29 (6)	0.08	30 (6)	30 (6)	0.01
Serum potassium, mEq/L	4.1 (0.5)	4.1 (0.5)	0.04	4.1 (0.5)	4.1 (0.5)	0.00
Serum creatinine, mg/dL	1.0 (0.4)	1.0 (0.3)	0.01	1.0 (0.3)	1.0 (0.3)	0.00
Albumin to creatinine ratio, mg/g	44 (167)	42 (156)	0.01	44 (166)	44 (162)	0.00
Total cholesterol, mg/dL	199 (40)	197 (43)	0.05	198 (41)	198 (43)	0.01
HDL-C, mg/dL	55 (14)	53 (15)	0.10	54 (14)	54 (15)	0.00
Triglycerides, mg/dL	124 (71)	130 (104)	0.06	128 (75)	128 (97)	0.00
Serum glucose, mg/dL	99 (15)	99 (13)	0.00	99 (15)	99 (13)	0.00
Medication use						
Aspirin	330 (45)	639 (49)	0.07	48	48	0.00
Statin	216 (30)	475 (36)	0.14	34	34	0.00
NSAID	244 (34)	444 (34)	0.01	33	34	0.01
No. of nonantihypertensive medications, mean (SD)	3 (3)	3 (3)	0.07	3 (3)	3 (3)	0.01
No. of antihypertensive medications, mean (SD)	2 (1)	2 (1)	0.10	2 (1)	2 (1)	0.00
CCB	300 (41)	414 (32)	0.20	36	35	0.02
Thiazide diuretic	337 (46)	656 (50)	0.07	49	49	0.00
Loop diuretic	36 (5)	62 (5)	0.01	5	5	0.00
β-Blocker	237 (33)	417 (32)	0.02	32	32	0.01
α-Blocker	33 (5)	58 (4)	0.01	4	4	0.02
Other antihypertensive class	59 (8)	77 (6)	0.09	7	7	0.01
Intensive treatment arm	453 (62)	828 (63)	0.02	63	63	0.00

Abbreviations: ACEI, angiotensin-converting enzyme inhibitor; ARB, angiotensin-II receptor blocker; ASMD, absolute standardized mean difference; BMI, body mass index (calculated as weight in kilograms divided by height in meters squared); CCB, calcium channel blocker; CVD, cardiovascular disease; HDL-C, high-density lipoprotein cholesterol; IP, inverse probability; MoCA, Montreal Cognitive Assessment; NSAID, nonsteroidal anti-inflammatory drug; SPRINT, Systolic Blood Pressure Intervention Trial.

SI conversion factors: To convert creatinine to micromoles per liter, multiply by 88.4; glucose to millimoles per liter, multiply by 0.0555; HDL-C and total cholesterol to micromoles per liter, multiply by 0.0259; potassium to millimoles per liter, multiply by 1; and triglycerides to millimoles per liter, multiply by 0.0113.

^a^ Data for before weighting are presented as number (percentage) of participants, and data for after weighting as percentage of participants unless otherwise indicated. The total numbers of patients in the post–inverse probability weighted columns were omitted because the numbers were slightly different as a result of the weighting.

^b^ Includes Asian, Hawaiian or Pacific Islander, Native American, and other as reported by the participant at baseline.

^c^ Scores range from 0 to 30, with higher scores denoting better cognitive function.

^d^ Subtest of the Wechsler Memory Scale. Scores range from 0 to 14, with higher scores denoting better cognitive function.

^e^ Subtest of the Wechsler Adult Intelligence Scale. Scores range from 0 to 135, with higher scores denoting better cognitive function.

### Statistical Analysis

The target trial for this study would randomize eligible participants to receive either an ARB or ACEI. In this target trial, we estimated the marginal cause-specific hazard ratios (HRs) and treatment-specific cumulative incidence functions using inverse probability (IP) of treatment weighting to account for baseline (ie, preinitiation) differences between participants initiating ARB vs ACEI new users for all primary and secondary outcomes.^19^ To estimate the IP of treatment weights, we fit a logistic regression model with new use of an ARB vs ACEI as the dependent variable, conditional on all pretreatment variables listed in Table 1. To prevent outliers with extreme weights from influencing our results, we truncated the IP of treatment weights at their 99th percentile. All 2-way interactions between each covariate and the binary subgroups listed herein were entered into the propensity score model and assessed using least absolute shrinkage and selection operator.^20^ All product terms between covariates and subgroup variables were shrunk to 0 by the least absolute shrinkage and selection operator procedure. Thus, our final propensity score model included only main effect terms. The overlap in the distribution of the propensity scores between ARB and ACEI new users was evaluated with histograms. We used the predicted probabilities from the logistic regression model to calculate each participant’s contribution to the IP-weighted analysis. We verified covariate balance overall and within subgroups^20^ before and after IP weighting; an absolute standardized mean difference greater than or equal to 0.1 was considered acceptable.^21^ To account for missing data (eTable 2 in Supplement 1), we used multiple imputation with chained equations with 10 imputed data sets.^22^ We recomputed the propensity score used for IP weighting separately within each imputed data set, and the effect size estimates were averaged across the imputed data sets. We computed an IP-weighted Aalen-Johansen estimator to generate the cumulative incidence function. The entire process, sans propensity score model selection, was bootstrapped to construct 95% CIs, using 2500 replicates.^23,24^

#### Subgroup and Sensitivity Analyses

We repeated the main analysis for the primary outcome in the following subgroups: age (<75 vs ≥75 years), sex (male vs female), race and ethnicity (Black vs all other race and ethnicity groups), randomized SBP treatment group (intensive vs standard), and baseline (unadjudicated) MCI status (based on race and ethnicity and education-specific MOCA thresholds).^25^ We performed 3 additional analyses to evaluate the robustness of our inferences. First, we repeated all analyses using overlap weights.^20^ Second, we repeated the main analysis after expanding the new-user identification window to the first 24 months of trial follow-up. Third, we assessed the possibility of residual bias due to selection, confounding, or misclassification by repeating our analytic approach with a composite negative control outcome of infectious, orthopedic, or hematologic serious adverse events (ie, outcomes thought not to be caused by the medication exposure groups). Negative control analyses were repeated in subgroups as in the primary analysis. All analyses were completed using R, version 4.0.2 (R Foundation for Statistical Computing).

## Results

### Participant Characteristics

Among 9361 participants enrolled in SPRINT, 5469 participants (58.4%) were not taking an ARB or ACEI at the baseline visit; 727 and 1313 were ARB or ACEI new users, respectively, within the first 12 months of follow-up (unweighted mean [SD] age, 67 [9.5] years; 1291 [63%] male; 749 [37%] female; 240 [33%] Black; 89 [12%] Hispanic; 383 [53%] White; and 15 [2%] other [Asian, Hawaiian or Pacific Islander, Native American, and other race or ethnicity]) (eFigure 2 in Supplement 1). Among ARB new users, the most common ARB was losartan (53.8%), followed by valsartan (44.3%), azilsartan (0.7%), and any other ARB (1.2%). Among ACEI new users, 99.4% initiated lisinopril and 0.6% initiated other ACEIs. The median baseline MoCA score was 24 (IQR, 21-26) and 23 (IQR, 20-26) among ARB and ECEI new users, respectively. Baseline characteristics before and after IP weighting are reported overall in Table 1 and in subgroups in eFigure 6 and eFigure 7 in Supplement 1. Before IP weighting, there were minimal differences in baseline characteristics between ARB and ACEI new users, except ARB new users were more likely to be women (308 [42%] vs 441 [34%]), Black (240 [33%] vs 374 [28%]), Hispanic (89 [12%] vs 100 [8%]), privately insured (340 [47%] vs 529 [40%]), and calcium channel blocker users (300 [41%] vs 414 [32%]); they were less likely to have Veterans Affairs insurance (88 [12%] vs 270 [21%]), history of cardiovascular disease (77 [11%] vs 200 [15%]), and current statin use (216 [30%] vs 475 [36%]). The region of common support was large (eFigure 8 in Supplement 1) and, after applying IP weighting, all baseline covariates were well balanced between treatment groups; all absolute standardized differences were less than 0.10 after weighting for each of the 50 covariates (eFigure 7 in Supplement 1). The ARB vs ACEI new users had similar achieved SBPs throughout the remainder of the follow-up period (eFigure 9 in Supplement 1).

### Primary Outcome

**Figure 1.**
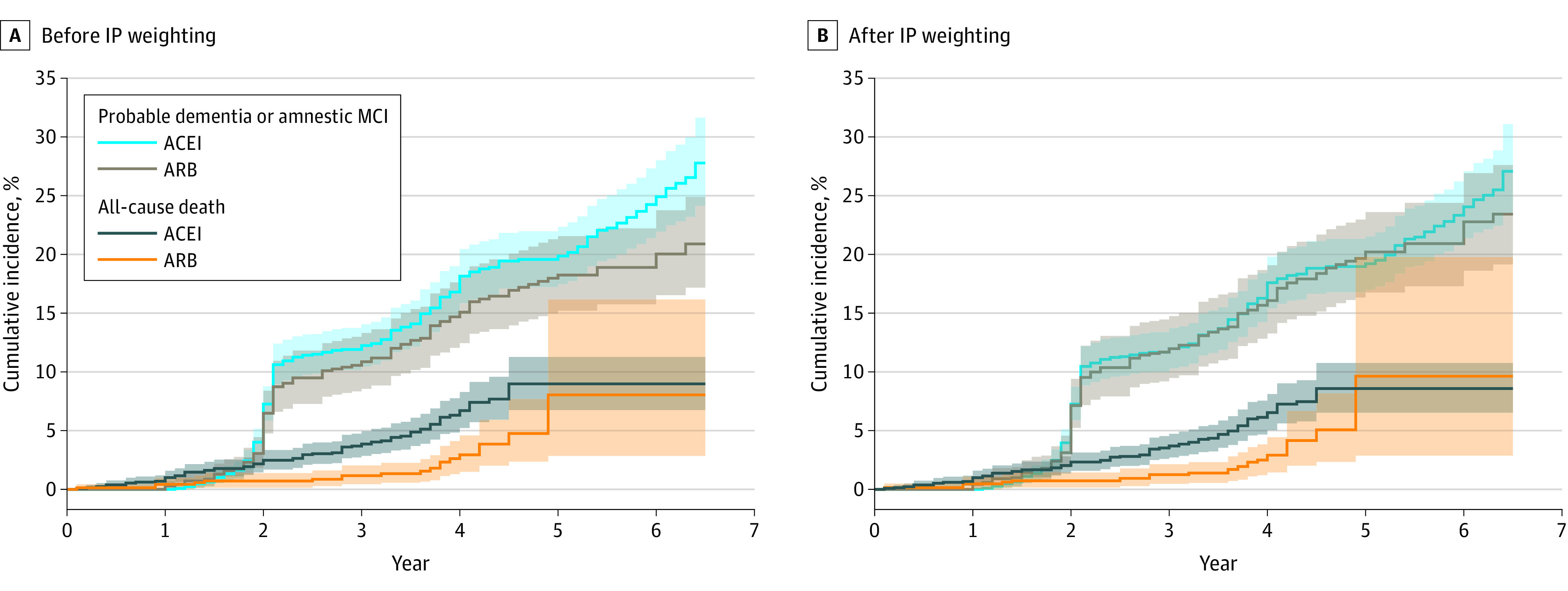
Cumulative Incidence of Amnestic Mild Cognitive Impairment or Probable Dementia and All-Cause Death Among New Users of an ARB- vs ACEI-Based Antihypertensive Medication Regimen Before and After IP Weighting Shown are the treatment-specific cumulative incidence functions for the primary outcome (a composite of amnestic MCI or probable dementia) in the unweighted (A) and inverse-probability weighted analysis (B). Numbers at risk are omitted because the cumulative incidence curves were generated from a model using weighted samples. ACEI indicates angiotensin-converting enzyme inhibitor; ARB, angiotensin II receptor blocker; IP, inverse probability; MCI, mild cognitive impairment.

Among the 2040 SPRINT participants included in the present analysis (n = 727 ARB new users and n = 1313 ACEI new users), amnestic MCI or PD occurred in 118 ARB and 260 ACEI new users over a median follow-up of 4.9 years. The unadjusted rate of the primary outcome was 3.9 vs 4.8 events per 100 person-years among ARB vs ACEI new users (HR, 0.80; 95% CI, 0.64-1.01) (Table 2 and Figure 1). The IP-weighted rate of the primary outcome was 4.3 vs 4.6 events per 100 person-years among ARB vs ACEI new users (HR, 0.93; 95% CI, 0.76-1.13) (Table 2 and Figure 1).

**Table 2.** Incidence Rates and Hazard Ratios for the Primary and Secondary Outcomes Among New Users of an ARB vs ACEI

Outcome	Unadjusted			IP weighted		
	No. (rate per 100 person-years)		Hazard ratio (95% CI)^a^	No. (rate per 100 person-years)		Hazard ratio (95% CI)^a^
	ARB new user (n = 727)	ACEI new user (n = 1313)		ARB new user	ACEI new user	
**Primary outcome**						
Amnestic MCI or PD	118 (3.9)	260 (4.8)	0.80 (0.64-1.01)	4.3	4.6	0.93 (0.76-1.13)
**Secondary outcomes**						
Analyses censoring death						
Protocol-defined MCI or PD	57 (1.8)	323 (2.2)	0.83 (0.60-1.13)	2.1	2.2	0.97 (0.72-1.31)
Probable dementia alone	20 (0.6)	41 (0.7)	0.85 (0.49-1.49)	0.7	0.7	1.02 (0.58-1.79)
Amnestic MCI alone	103 (3.5)	233 (4.4)	0.78 (0.61-0.99)	3.8	4.3	0.90 (0.72-1.12)
Protocol-defined MCI alone	44 (1.4)	96 (1.8)	0.82 (0.57-1.17)	1.7	1.7	0.97 (0.69-1.38)
Analysis incorporating death into the composite outcome						
Amnestic MCI, PD, or death	134 (4.4)	323 (5.9)	0.74 (0.60-0.91)	4.8	5.7	0.85 (0.71-1.02)
Protocol-defined MCI, PD, or death	74 (2.4)	196 (3.5)	0.68 (0.52-0.89)	2.7	3.4	0.79 (0.61-1.03)
Probable dementia or death	37 (1.1)	115 (2.0)	0.58 (0.39-0.84)	1.3	1.9	0.67 (0.46-0.98)
Amnestic MCI or death	120 (3.9)	298 (5.5)	0.72 (0.58-0.89)	4.3	5.2	0.83 (0.68-1.01)
Protocol-defined MCI or death	62 (2.0)	170 (3.0)	0.66 (0.49-0.89)	2.3	2.9	0.77 (0.58-1.03)
Death	19 (0.7)	78 (1.6)	0.43 (0.25-0.73)	0.8	1.6	0.48 (0.28-0.82)

Abbreviations: ACEI, angiotensin-converting enzyme inhibitor; ARB, angiotensin II receptor blocker; IP, inverse probability; MCI, mild cognitive impairment; PD, probable dementia.

^a^ The 95% CI values were constructed using SEs from 2500 bootstrap samples, assuming normal distribution.

### Secondary Outcomes

A total of 336 amnestic MCI events, 61 PD events, and 97 deaths occurred during follow-up. There was no appreciable difference between ARB vs ACEI new users regarding the IP-weighted rate of the individual components of the primary outcome (amnestic MCI: HR, 0.90; 95% CI, 0.72-1.12; or PD: HR, 1.02; 95% CI, 0.58-1.79). However, ARB new users had a lower IP-weighted risk of death compared with ACEI new users (HR, 0.48; 95% CI, 0.28-0.82). ARB new users also had a lower IP-weighted risk of the composite outcomes of PD or death (HR, 0.67; 95% CI, 0.46-0.98), amnestic MCI or death (HR, 0.83; 95% CI, 0.68-1.01), and PD or amnestic MCI or death (HR, 0.85; 95% CI, 0.71-1.02). Results of other secondary outcomes are presented in Table 2 and eFigure 10 in Supplement 1.

### Serious Adverse Events

Serious adverse events occurred in 39 ARB new users (IP weighted, 1.7 per 100 person-years) and 87 ACEI new users (IP weighted, 2.0 per 100 person-years) (HR, 0.84; 95% CI, 0.56-1.20) (eTable 3 in Supplement 1). There was a lower rate of bradycardia and hypotension among ARB vs ACEI new users, with no difference in the rates of other adverse events.

### Sensitivity and Subgroup Analyses

**Figure 2.**
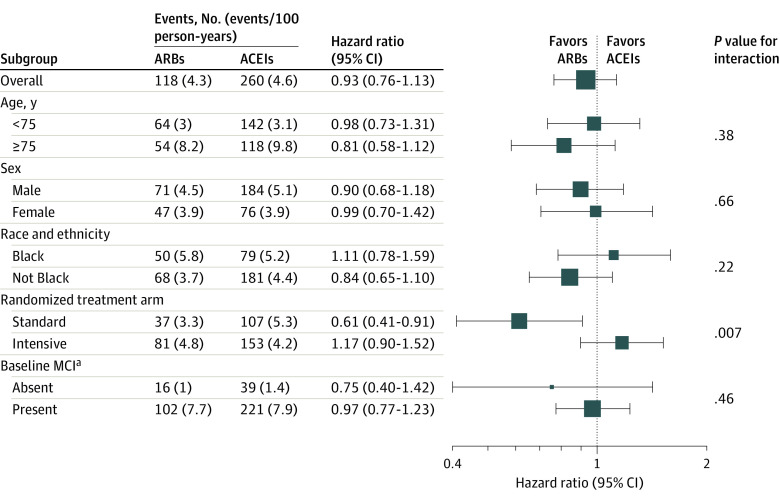
IP-Weighted Primary Outcome Results in Subgroups Among New Users of an ARB- vs ACEI-Based Antihypertensive Medication Regimen For statistical purposes, race and ethnicity subgroups were categorized as binary. The Not Black category comprises Hispanic, White, and other race and ethnicity categories. ACEI indicates angiotensin-converting enzyme inhibitor; ARB, angiotensin-II receptor blocker; IP, inverse probability; MCI, mild cognitive impairment. ^a^Unadjudicated. Based on race-and education-specific Montreal Cognitive Assessment thresholds.

Results were similar within subgroups with 1 exception: ARB new users had a lower IP-weighted risk of amnestic MCI or PD among those in the standard SBP treatment arm (Figure 2; eTable 4 in Supplement 1) (HR, 0.61; 95% CI, 0.41-0.91) but not in the intensive arm (HR, 1.17; 95% CI, 0.90-1.52) (*P* = .007 for interaction). Results were similar when repeating all analyses using overlap weights (eTables 5-7; eFigure 11 and eFigure 12 in Supplement 1). Results were also similar when extending the new-user identification window to 24 months (eTable 8 in Supplement 1). There was no appreciable difference between ARB vs ACEI new users regarding the rate of the negative control outcome. However, in subgroup analyses, the point estimate suggested a lower risk of the composite negative control outcome in ARB vs ACEI new users in the standard treatment arm, although evidence of an interaction was weak (eTable 9 in Supplement 1) (standard arm: HR, 0.61; 95% CI, 0.33-1.13; intensive arm: HR, 0.97; 95% CI, 0.67-1.40; *P* = .20 for interaction).

## Discussion

We estimated the comparative effectiveness of an ARB vs ACEI on cognitive outcomes among SPRINT participants and did not find evidence of an appreciable difference during 4.9 years of follow-up in the overall study population. In a subgroup analysis, ARB vs ACEI new users had an appreciably lower risk of amnestic MCI or PD in the standard SBP treatment arm but not in the intensive treatment arm, suggesting that the benefits of intensive SBP control may have diminished any potential protective association of ARBs vs ACEIs on cognitive outcomes. Findings were consistent across several sensitivity analyses. In the negative control outcome analysis, the point estimate for the standard treatment subgroup suggested that residual confounding may be present.

Our findings should be interpreted in the context of previous literature on this topic.^1,11,26,27,28^ A network meta-analysis of 13 734 patients in 19 RCTs reported that ARBs were superior to ACEIs in preventing cognitive decline.^11^ In addition, 2 small RCTs demonstrated that ARBs improved episodic memory and visuospatial abilities compared with ACEIs.^26,27^ These studies did not assess the risk of adjudicated amnestic MCI or PD. In addition, BP control in the populations from these prior studies most closely represented the standard treatment arm of SPRINT, with mean achieved SBPs in the 130s to 140s mm Hg during follow-up. Accordingly, our subgroup analysis noting a lower adjusted risk of amnestic MCI or PD with ARB vs ACEI initiation in the SPRINT standard, but not the intensive SBP treatment arm is consistent with previous literature. Although results from this subgroup analysis should be interpreted with caution, this finding may indicate that, in the absence of intensive SBP control (<120 mm Hg), which applies to most US adults with hypertension, there may be a lower adjusted risk of amnestic MCI or PD with ARB vs ACEI initiation.^28^ However, in the setting of intensive BP control, the specific renin-angiotensin system blocker (ARB or ACEI) may not meaningfully affect cognitive outcomes due to the substantial, independent benefit of intensive BP control on cognitive outcomes.^1^ In addition, we observed a lower risk of death among ARB vs ACEI initiators (HR, 0.48; 95% CI, 0.28-0.82). Future work should explore cause-specific mortality differences between ARB vs ACEI initiators to better understand this result.

While the overall adjusted analysis did not find an appreciable difference between ARB vs ACEI initiation and amnestic MCI or PD (HR 0.93 [95% CI 0.76-1.13]), the direction of the point estimate in the overall analysis was consistent with our hypothesis and results from the previous network meta-analysis.^11^ There are several potential explanations for why we did not observe a lower adjusted risk of amnestic MCI or PD with ARB vs ACEI initiation in the overall analysis. First, earlier studies did not incorporate intensive SBP control as part of the treatment intervention. Second, we had a relatively smaller sample size and limited number of events compared with the earlier meta-analysis, leading to uncertainty around our association estimates.^11^ Third, long-term cognitive benefits of ARBs may be due to properties of specific medications within this class: ARBs that penetrate the blood-brain barrier may yield greater benefit than ARBs that do not.^29^ There was little variation in the individual ARBs (54% losartan and 44% valsartan) and ACEIs (99% lisinopril), limiting our ability to precisely estimate differences in risks of cognitive outcomes between initiators of specific antihypertensive medications in the present analysis.

### Strengths and Limitations

Our study is strengthened by using prospectively collected data among participants of a high-quality RCT, including medication use and carefully adjudicated cognitive end points. The study leveraged adjudicated clinical end points of amnestic MCI and PD, which provide robust evidence of early cognitive dysfunction that is associated with progression to dementia.^18,30,31^ In addition, we used methods of target trial emulation that included an active comparator new-user design with robust adjustment for a rich set of sociodemographic, clinical, cognitive, and pharmacologic pretreatment covariates using IP weighting to balance participant characteristics across exposure groups.

This study has limitations. Although we used an active-comparator new-user design, the risk of confounder measurement error is possible because some confounder values were measured at randomization but not at the date of treatment initiation. Because we have data on self-reported current use of an ARB or ACEI only at the baseline visit, new users during trial follow-up may include both first-time users of an ARB or ACEI and those who took these agents previously but not at the time of the baseline visit; nonetheless, this possibility is unlikely to be differential between ARB vs ACEI user. It is also possible that a participant may have started and stopped an antihypertensive medication between study visits that was not captured by the parent study or in the present analysis. Although prevalent dementia at baseline was an exclusion criterion in SPRINT, the trial did not adjudicate cognitive status at baseline. Therefore, we cannot exclude or examine the influence of prevalent MCI at the time of randomization or at the time of ARB or ACEI initiation. Given the relatively similar MoCA scores across exposure groups at baseline, we do not anticipate that this impacted the results differentially. Furthermore, the subgroup analysis among participants without cognitive impairment at baseline indicated a potential benefit of ARB vs ACEI on reducing the risk of amnestic MCI or PD (HR, 0.75; 95% CI, 0.40-1.42), although this analysis was limited in statistical power. This suggests that including participants with MCI at baseline could have biased our main results toward the null. Furthermore, the negative control analyses demonstrated a similar point estimate to the primary end point analyses when stratified by standard vs intensive treatment arm, suggesting that residual confounding may exist. The negative control outcomes were selected empirically and have not been previously validated in this study population. Consequently, the findings may also be explained by other theoretical biologic differences between ARBs and ACEIs that were not adequately accounted for in selecting the negative control outcomes.^32,33,34^

## Conclusions

In this cohort study, we did not find evidence of an appreciable lower adjusted risk of amnestic MCI or PD with ARB vs ACEI initiation in the overall study population. However, in a subgroup analysis, we did find evidence of lower risk of amnestic MCI or PD among ARB vs ACEI new users in the standard SBP treatment arm, suggesting that the benefits of intensive BP control may have diminished any potential beneficial effects of ARBs compared with ACEIs. This may indicate that, in the absence of intensive SBP control (<120 mm Hg)—a scenario affecting most US adults with hypertension—there may be a lower risk of cognitive outcomes with ARB vs ACEI use. The results suggest the need for a pragmatic RCT of ARBs vs ACEIs, with BP management reflective of community practice outside clinical trial settings of intensive BP treatment, on important cognitive outcomes.

## Notes

Supplement 1. **eMethods.** Detailed Methods **eFigure 1.** Treatment Algorithms in SPRINT **eFigure 2.** Flow Chart to Identify New Users of ARBs and ACEIs in SPRINT for the Present Analysis **eFigure 3.** Distribution of the Time in Days From Randomization to Initiation of ACEI or ARB in SPRINT **eFigure 4.** Overview of the Study Design **eFigure 5.** Timeline for Follow-up Cognitive Assessments in SPRINT **eFigure 6.** Balance of Pretreatment Characteristics Between New Users of an ARB vs ACEI Before Inverse Probability Weighting Overall and Within Subgroup **eFigure 7.** Balance of Pretreatment Characteristics Between New Users of an ARB vs ACEI After Inverse Probability Weighting Overall and Within Subgroups **eFigure 8.** Distribution of Propensity Scores Among ARB vs ACEI New Users **eFigure 9.** Follow-up Systolic Blood Pressure Among New Users of an ARB and ACEI (Unweighted) **eFigure 10.** IP-Weighted Cumulative Incidence Curves for Secondary Outcomes **eFigure 11.** Balance of Pretreatment Characteristics Between New Users of an ARB vs ACEI After Overlap Weighting Overall and Within Subgroups **eFigure 12.** Overlap-Weighted Cumulative Incidence Curves for Secondary Outcomes **eTable 1.** Components of the SPRINT Cognitive Battery **eTable 2.** Baseline Characteristics of New Users of an ARB vs ACEI Before Weighting Using All Available Observed Data **eTable 3.** IP-Weighted Serious Adverse Events Among ARB vs ACEI New Users **eTable 4.** IP-Weighted Primary Outcome Results in Subgroups Among ARB vs ACEI New Users **eTable 5.** Incidence Rates and Hazard Ratios for the Primary and Secondary Outcomes Among New-Users of an ARB vs ACEI, Unweighted and Overlap-Weighted **eTable 6.** Overlap-Weighted Serious Adverse Events Among ARB vs ACEI New Users **eTable 7.** Overlap-Weighted Primary Outcome Results in Subgroups Among ARB vs ACEI New Users **eTable 8.** IP-Weighted Primary Outcome Results Among ARB vs ACEI New-Users, Varying the Definition of the Initiation Window **eTable 9.** Negative Control Outcomes Analysis
eReferences

Supplement 2.
Trial Protocol
