# Supplementary material for: Risk of Mild Cognitive Impairment or Probable Dementia in New Users of Angiotensin II Receptor Blockers and Angiotensin-Converting Enzyme Inhibitors: A Secondary Analysis of Data From the Systolic Blood Pressure Intervention Trial (SPRINT)
Source: JAMA Netw Open. 2022 Jul 14;5(7):e2220680. doi: 10.1001/jamanetworkopen.2022.20680 (PMC9284332; doi:10.1001/jamanetworkopen.2022.20680)
Supplement: Supplement 1. — eMethods. Detailed Methods eFigure 1. Treatment Algorithms in SPRINT eFigure 2. Flow Chart to Identify New Users of ARBs and ACEIs in SPRINT for the Present Analysis eFigure 3. Distribution of the Time in Days From Randomization to Initiation of ACEI or ARB in SPRINT eFigure 4. Overview of the Study Design eFigure 5. Timeline for Follow-up Cognitive Assessments in SPRINT eFigure 6. Balance of Pretreatment Characteristics Between New Users of an ARB vs ACEI Before Inverse Probability Weighting Overall and Within Subgroup eFigure 7. Balance of Pretreatment Characteristics Between New Users of an ARB vs ACEI After Inverse Probability Weighting Overall and Within Subgroups eFigure 8. Distribution of Propensity Scores Among ARB vs ACEI New Users eFigure 9. Follow-up Systolic Blood Pressure Among New Users of an ARB and ACEI (Unweighted) eFigure 10. IP-Weighted Cumulative Incidence Curves for Secondary Outcomes eFigure 11. Balance of Pretreatment Characteristics Between New Users of an ARB vs ACEI After Overlap Weighting Overall and Within Subgroups eFigure 12. Overlap-Weighted Cumulative Incidence Curves for Secondary Outcomes eTable 1. Components of the SPRINT Cognitive Battery eTable 2. Baseline Characteristics of New Users of an ARB vs ACEI Before Weighting Using All Available Observed Data eTable 3. IP-Weighted Serious Adverse Events Among ARB vs ACEI New Users eTable 4. IP-Weighted Primary Outcome Results in Subgroups Among ARB vs ACEI New Users eTable 5. Incidence Rates and Hazard Ratios for the Primary and Secondary Outcomes Among New-Users of an ARB vs ACEI, Unweighted and Overlap-Weighted eTable 6. Overlap-Weighted Serious Adverse Events Among ARB vs ACEI New Users eTable 7. Overlap-Weighted Primary Outcome Results in Subgroups Among ARB vs ACEI New Users eTable 8. IP-Weighted Primary Outcome Results Among ARB vs ACEI New-Users, Varying the Definition of the Initiation Window eTable 9. Negative Control Outcomes Analysis eReferences [file jamanetwopen-e2220680-s001.pdf]

Supplementary Online Content

Cohen JB, Marcum ZA, Zhang C, et al; Systolic Blood Pressure Intervention Trial (SPRINT) Research Group. Risk of mild cognitive impairment or probable dementia in new users of angiotensin II receptor blockers and angiotensin-converting enzyme inhibitors: a secondary analysis of data from the Systolic Blood Pressure Intervention Trial (SPRINT). *JAMA Netw Open*. 2022;5(7):e2220680. doi:10.1001/jamanetworkopen.2022.20680

eMethods. Detailed Methods

eFigure 1. Treatment Algorithms in SPRINT

eFigure 2. Flow Chart to Identify New Users of ARBs and ACEIs in SPRINT for the Present Analysis

eFigure 3. Distribution of the Time in Days From Randomization to Initiation of ACEI or ARB in SPRINT

eFigure 4. Overview of the Study Design

eFigure 5. Timeline for Follow-up Cognitive Assessments in SPRINT

eFigure 6. Balance of Pretreatment Characteristics Between New Users of an ARB vs ACEI Before Inverse Probability Weighting Overall and Within Subgroup

eFigure 7. Balance of Pretreatment Characteristics Between New Users of an ARB vs ACEI After Inverse Probability Weighting Overall and Within Subgroups

eFigure 8. Distribution of Propensity Scores Among ARB vs ACEI New Users

eFigure 9. Follow-up Systolic Blood Pressure Among New Users of an ARB and ACEI (Unweighted)

eFigure 10. IP-Weighted Cumulative Incidence Curves for Secondary Outcomes

eFigure 11. Balance of Pretreatment Characteristics Between New Users of an ARB vs ACEI After Overlap Weighting Overall and Within Subgroups

eFigure 12. Overlap-Weighted Cumulative Incidence Curves for Secondary Outcomes

eTable 1. Components of the SPRINT Cognitive Battery

eTable 2. Baseline Characteristics of New Users of an ARB vs ACEI Before Weighting Using All Available Observed Data

eTable 3. IP-Weighted Serious Adverse Events Among ARB vs. ACEI New Users

eTable 4. IP-Weighted Primary Outcome Results in Subgroups Among ARB vs ACEI New Users

eTable 5. Incidence Rates and Hazard Ratios for the Primary and Secondary Outcomes Among New-Users of an ARB vs ACEI, Unweighted and Overlap-Weighted

eTable 6. Overlap-Weighted Serious Adverse Events Among ARB vs ACEI New Users

eTable 7. Overlap-Weighted Primary Outcome Results in Subgroups Among ARB vs ACEI New Users

eTable 8. IP-Weighted Primary Outcome Results Among ARB vs. ACEI New-Users, Varying the Definition of the Initiation Window

eTable 9. Negative Control Outcomes Analysis

eReferences

This supplementary material has been provided by the authors to give readers additional information about their work.

© 2022 Cohen JB et al. *JAMA Network Open.*

© 2022 Cohen JB et al. *JAMA Network Open.*

eMethods. Detailed Methods

Assessment and adjudication of cognitive outcomes in SPRINT

Ascertaining cognitive status involved 3 steps. First, in-person cognitive screening assessments were administered to all participants at baseline and during follow-up by centrally trained and certified examiners at each local site. Assessments included a test of global cognitive function (Montreal Cognitive Assessment [MoCA]; range, 0-30), learning and memory (Logical Memory forms I and II subtests of the Wechsler Memory Scale; ranges, 0-28 and 0-14), and processing speed (Digit Symbol Coding Test of the Wechsler Adult Intelligence Scale; range, 0-135). For white participants scoring lower than 19 (with <12 years of education) or lower than 21 (with ≥12 years of education) on the MoCA, non-white participants scoring lower than 17 (with <12 years of education) or lower than 19 (with ≥12 years of education) on the MoCA, or any participant with a decrease of 5 or more points from a previous MoCA assessment, a preidentified proxy was administered the Functional Activities Questionnaire, a 10-item measure of functional abilities (range, 0-30).

Second, participants scoring either higher than 0 on the Functional Activities Questionnaire or scoring 1 or lower on the 5 - point Delayed Recall subtest of the MoCA underwent further testing using an extended cognitive battery that measured attention/concentration, verbal and nonverbal memory, language, and executive functions. For participants who could not be assessed in persons during follow-up, a validated telephone battery was administered. For participants receiving the telephone battery, the Functional Activities Questionnaire was administered if the participant

© 2022 Cohen JB et al. *JAMA Network Open.*

scored below a preset cut point (≤31) on the Modified Telephone Interview for Cognitive Status. If a participant had died or was otherwise unable to communicate by telephone, the Dementia Questionnaire was administered to a prespecified contact. For all tests and questionnaires, validated Spanish translations were used when available. Otherwise, instruments were translated and then back-translated.

Third, in addition to cognitive test scores and proxy functional status reports, all participants were administered a standardized measure of depressive symptoms, perceived health status, quality of life, current medications, medical problems, and current health habits (smoking, alcohol use, and physical activity). Hospitalizations were also recorded as part of a standardized protocol for ascertainment of serious adverse events with all references to treatment group redacted. These data were reviewed by an expert adjudication panel that included a neurologist, neuropsychologists, geriatricians, and geropsychologists to adjudicate cognitive status. The adjudicators were masked to treatment status. Participants were classified into 1 of 3 primary categories: no cognitive impairment, MCI, or probable dementia. Unclassifiable cases were placed in a “cannot classify” category. Each case was reviewed independently by 2 adjudicators using standardized diagnostic criteria for probable dementia and MCI. Agreements bythe2adjudicators were final. Disagreements were discussed by the full panel on regularly scheduled conference calls, with the classification decision achieved by a majority vote of the panel members. No subclassification of probable dementia was made. Additional details of the adjudication process can be found in the trial protocol.

© 2022 Cohen JB et al. *JAMA Network Open.*

Data Availability and Access

The authors take full responsibility for the data, the analyses and interpretation, and the conduct of the research. We had full access to all data, and we have the right to publish any and all data, separate and apart from the guidance of any sponsor. Limited versions of the data sets may be available through the Biologic Specimen and Data Repository Information Coordinating Center from the National Heart, Lung, and Blood Institute. Our analytic code is available to interested researchers from the corresponding author upon reasonable request. This study was reviewed and approved by the University of Utah Institutional Review Board. The study protocols for SPRINT and the included cohorts were approved by the institutional review boards at each participating institution, and all participants provided written informed consent. The funders had no role in study design, analysis, preparation of the article, or the decision to submit the article for publication.

© 2022 Cohen JB et al. *JAMA Network Open.*

The SPRINT Study Population

The SPRINT design and main results for cardiovascular disease and cognitive outcomes have been previously reported.[1-3] Briefly, SPRINT was a randomized trial of intensive (target goal of <120 mm Hg) vs. standard systolic blood pressure (SBP) control (target goal of <140 mm Hg) among US adults age ≥50 years with high cardiovascular disease risk and SBP 130-180 mm Hg (depending on the number of antihypertensive medications being taken). Key exclusion criteria in SPRINT included those with diabetes, history of stroke, or heart failure. Individuals were also excluded if they were living in a nursing home, had a diagnosis of dementia, or were receiving medications primarily for dementia. A total of 9,361 participants were randomized at 102 clinical sites across the United States and Puerto Rico between November 2010 and March 2013. After randomization, participants' antihypertensive medications were adjusted to achieve their target SBP goal. The SPRINT treatment algorithm is provided in the supplement (**eFigure 1**). All major antihypertensive medication classes were included and provided at no cost to the participants. Participants were seen monthly for the first 3 months and every 3 months thereafter. Additional visits were scheduled as needed for management of adverse effects or for monitoring significant medication changes or other clinical issues. SPRINT was approved by the institutional review board at each participating site, and each participant provided written informed consent. The current analysis was approved by the Institutional Review Board at the University of Utah.

© 2022 Cohen JB et al. *JAMA Network Open.*

eFigure 1. Treatment algorithms in SPRINT.[4]

Panel A. Treatment algorithm for intensive blood pressure treatment (goal SBP <120 mm Hg).

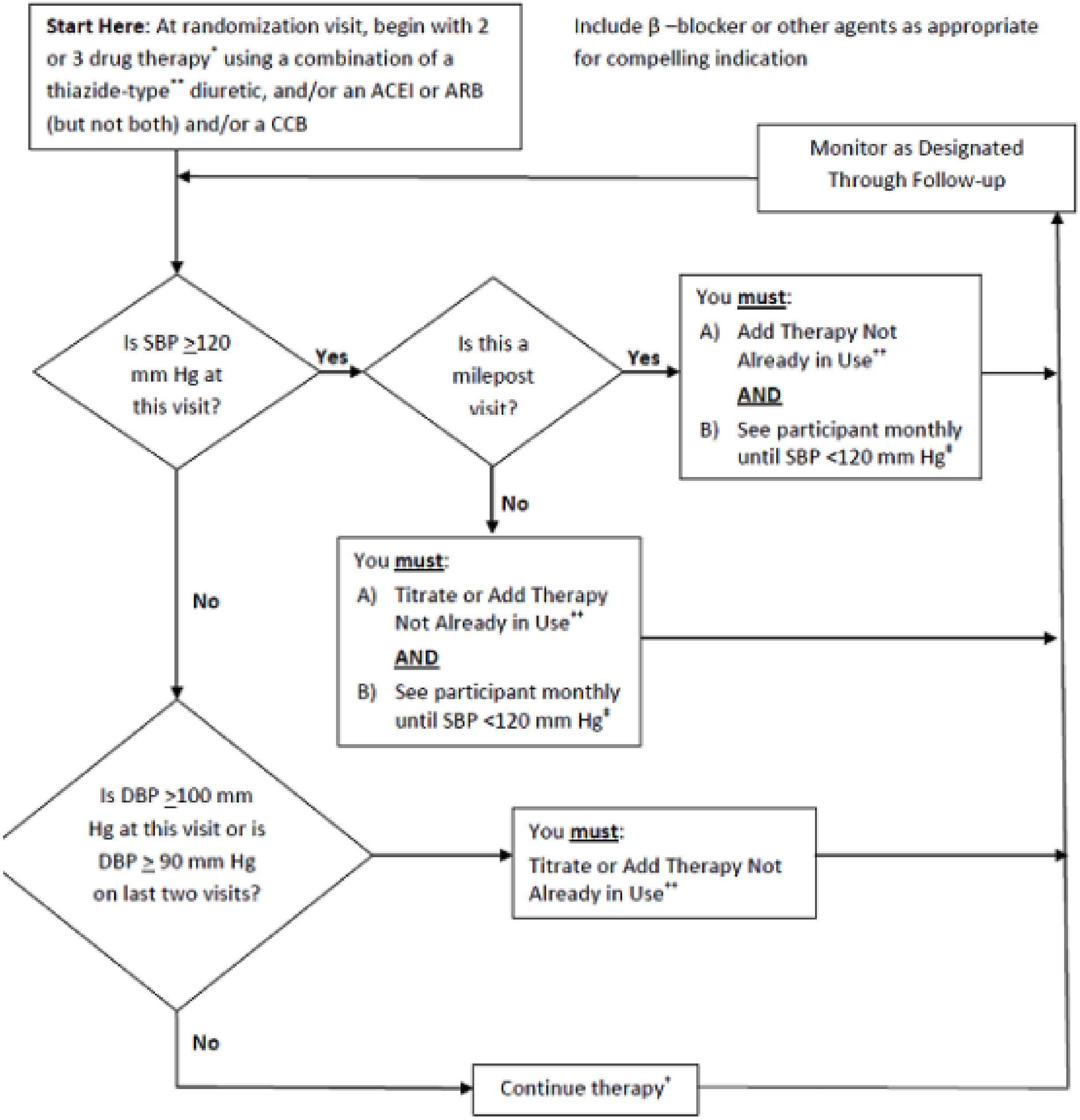

* May begin with a single agent for participants 75 years old or older with SBP < 140 on 0-1 meds at study entry. A second medication should be added at the 1 Month visit if participant is asymptomatic and SBP ≥ 130.

** May use loop diuretic for participants with advanced CKD

† Unless side effects warrant change in therapy

†† Consider consulting with the Clinical Center Network before adding a fifth anti-hypertensive medication

‡ Or until clinical decision made that therapy should not be increased further

Panel B. Treatment algorithm for standard blood pressure treatment (goal SBP <140 mm Hg).

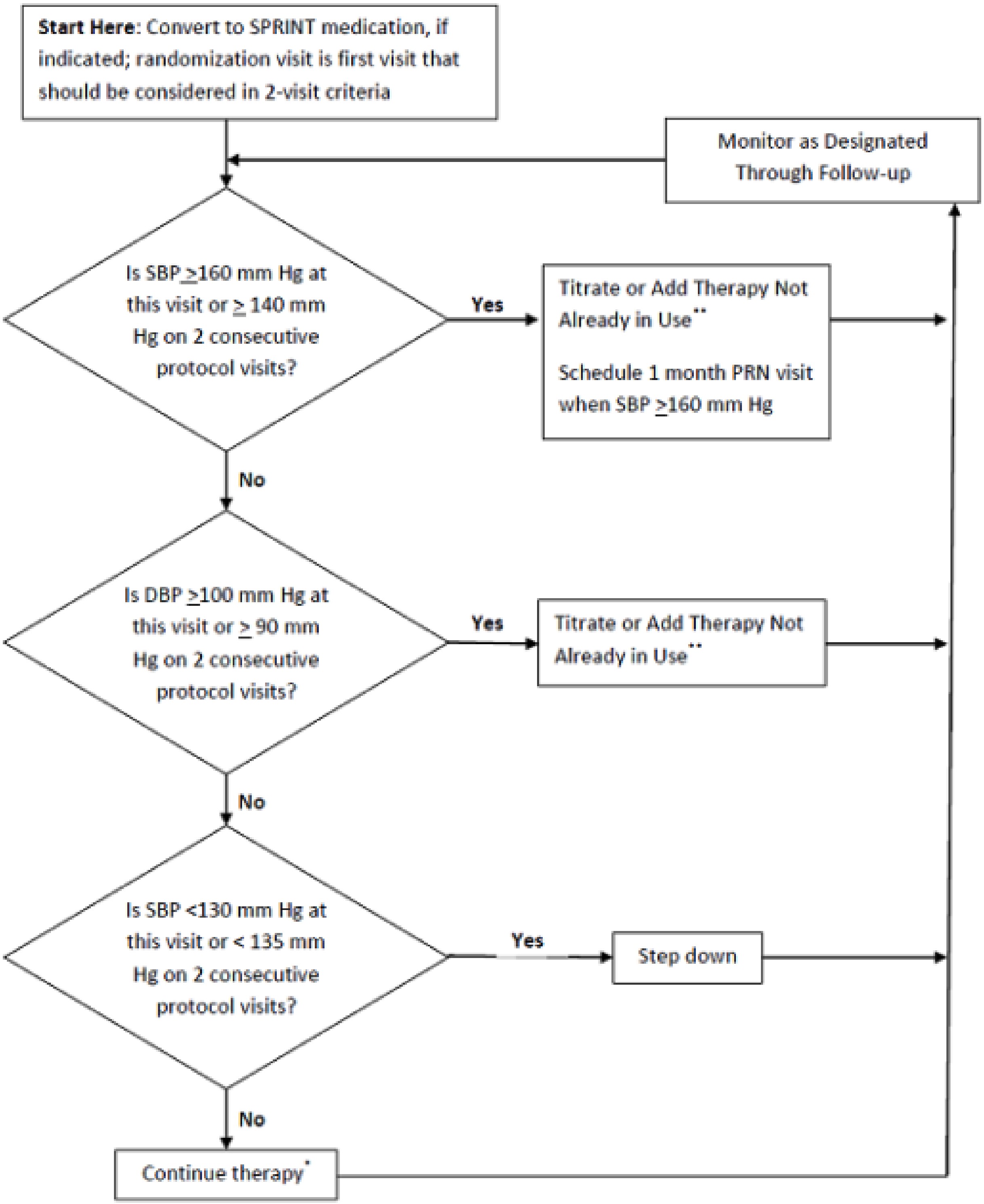

Include β –blocker or other agents as appropriate for compelling indications

* Unless side effects warrant change in therapy

** Consider consulting with the Clinical Center Network before adding a fifth anti-hypertensive medication

© 2022 Cohen JB et al. *JAMA Network Open.*

eFigure 2. Flow chart to identify new users of ARBs and ACEIs in SPRINT for the present analysis.

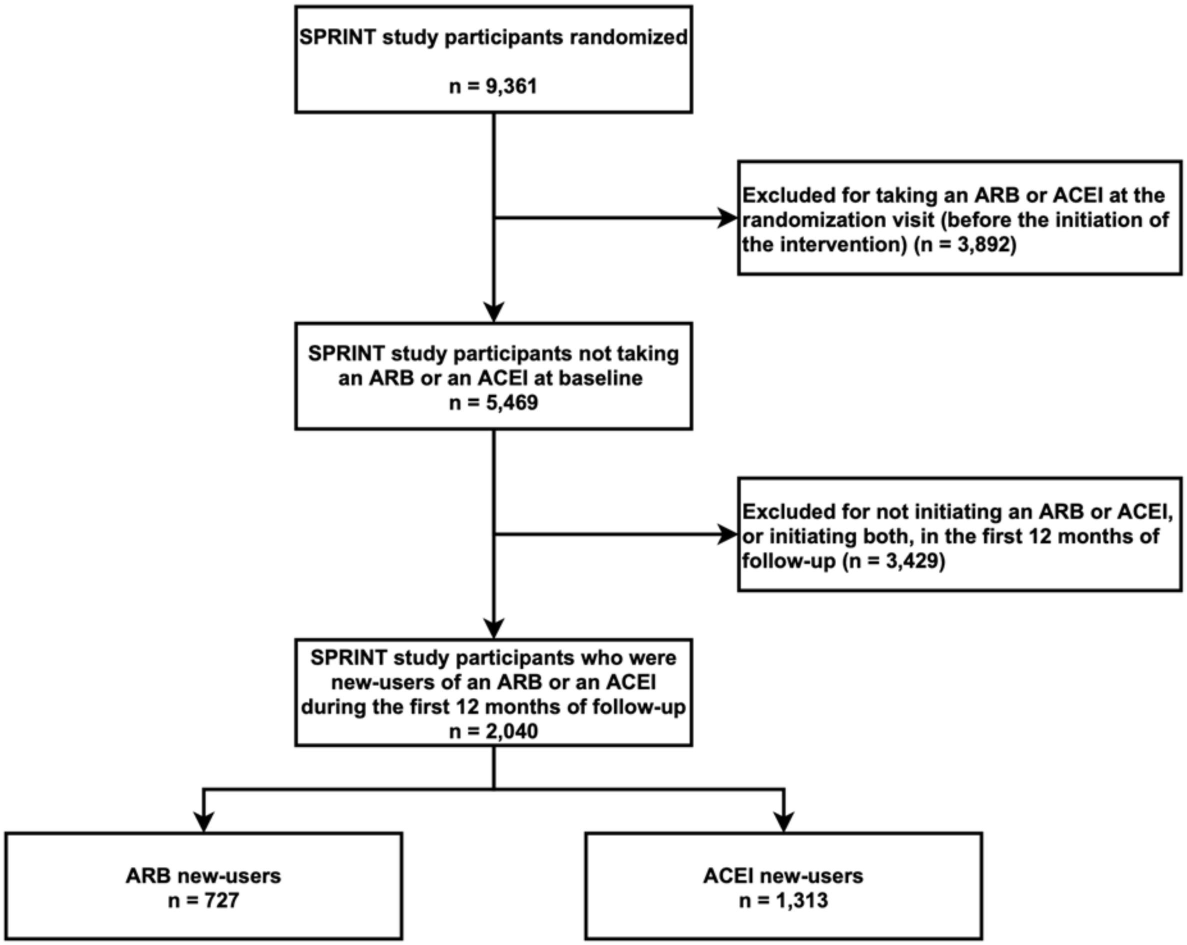

Abbreviations: ACEI: angiotensin-converting enzyme inhibitor; ARB: angiotensin II receptor blocker; SPRINT: Systolic Blood Pressure Intervention Trial

© 2022 Cohen JB et al. *JAMA Network Open.*

eFigure 3. Distribution of the time in days from randomization to initiation of ACEI or ARB in SPRINT.

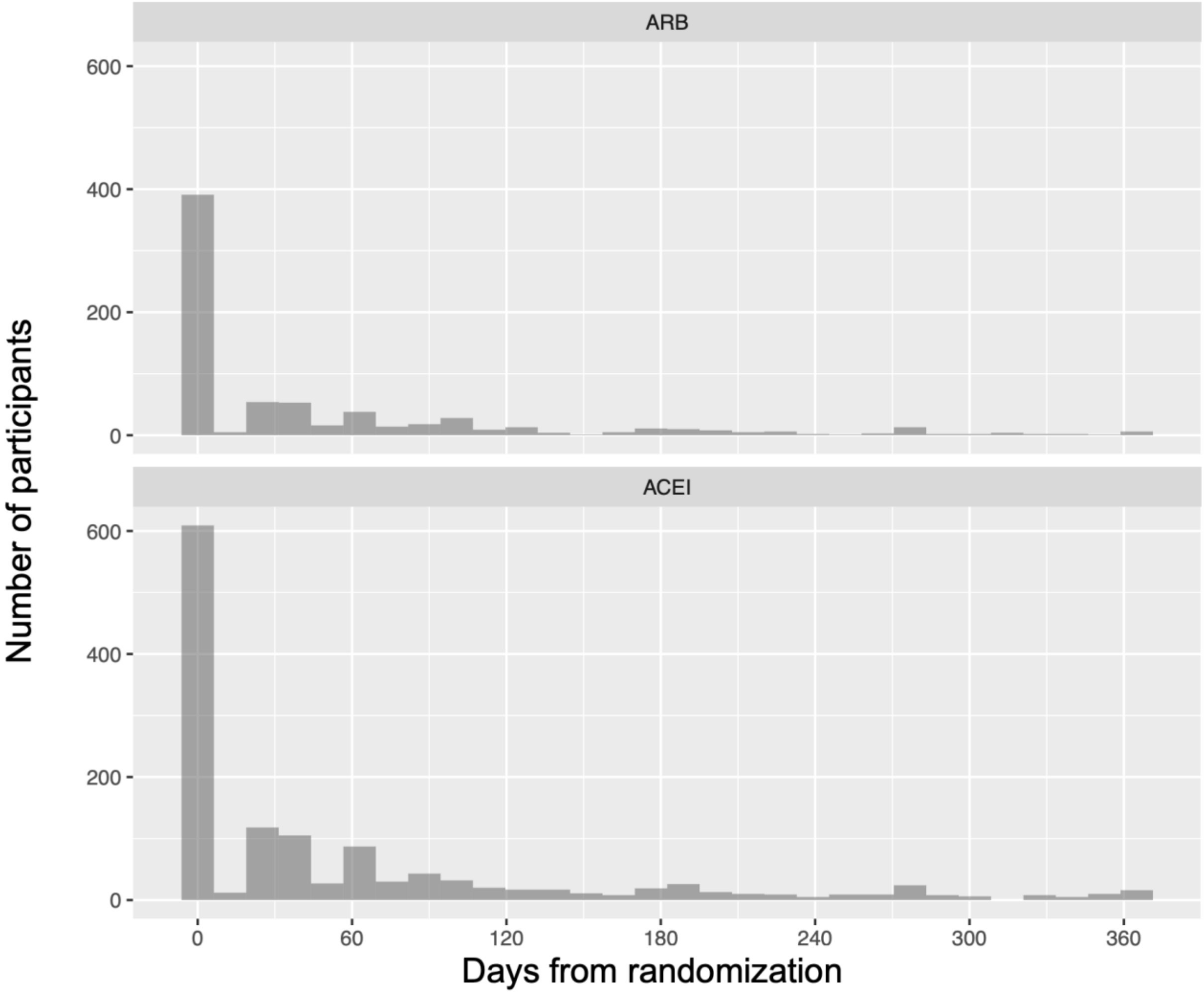

Abbreviations: ACEI: angiotensin-converting enzyme inhibitor; ARB: angiotensin II receptor blocker; SPRINT: Systolic Blood Pressure Intervention Trial

© 2022 Cohen JB et al. *JAMA Network Open.*

eFigure 4. Overview of the study design.

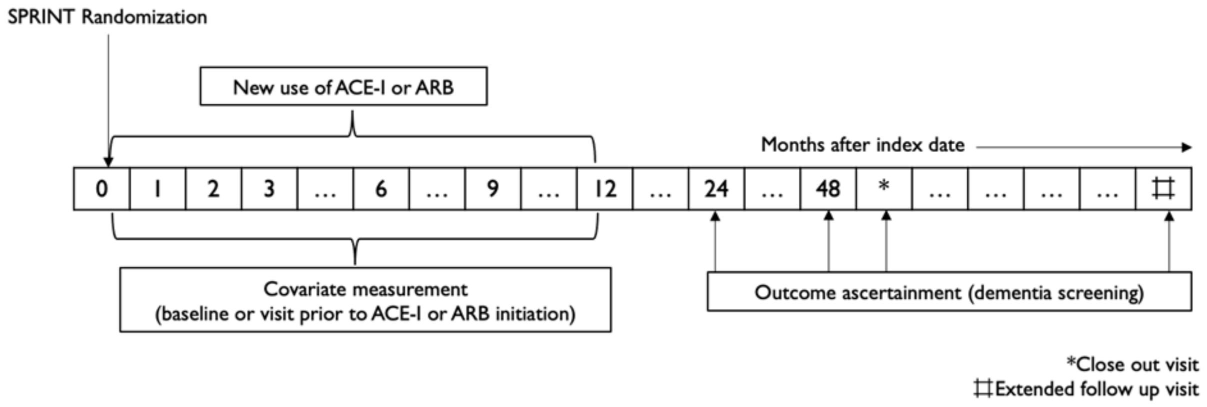

*****Participants were followed for a median of 4.9 years

Abbreviations: ACEI: angiotensin-converting enzyme inhibitor; ARB: angiotensin II receptor blocker; SPRINT: Systolic Blood Pressure Intervention Trial

© 2022 Cohen JB et al. *JAMA Network Open.*

eFigure 5. Timeline for follow-up cognitive assessments in SPRINT.

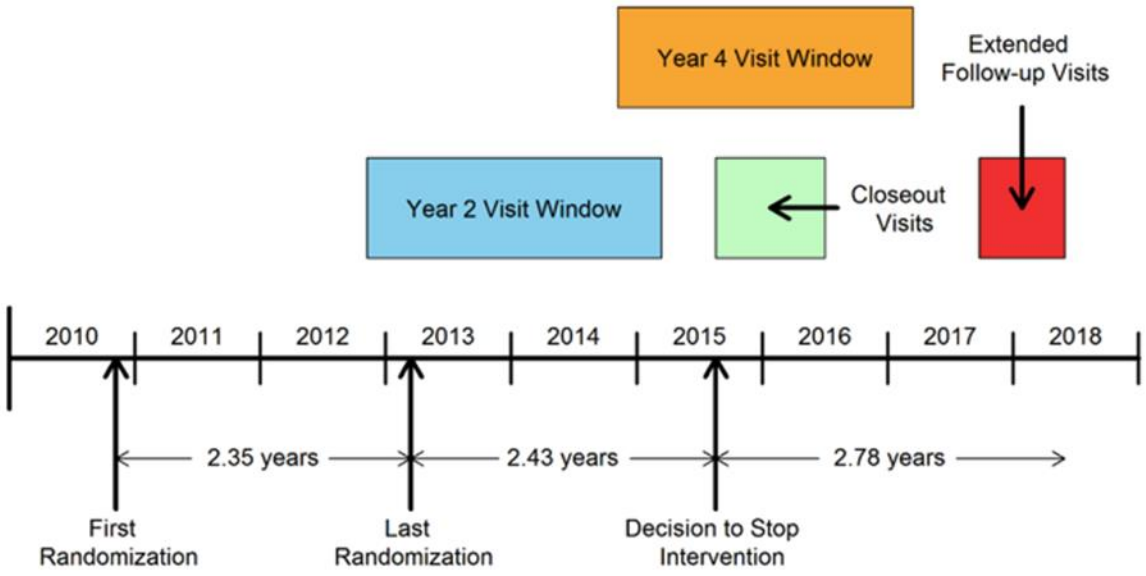

Abbreviations: SPRINT: Systolic Blood Pressure Intervention Trial

© 2022 Cohen JB et al. *JAMA Network Open.*

eFigure 6. Balance of pre-treatment characteristics between new users of an ARB vs. ACEI *before* inverse probability weighting overall and within subgroups.

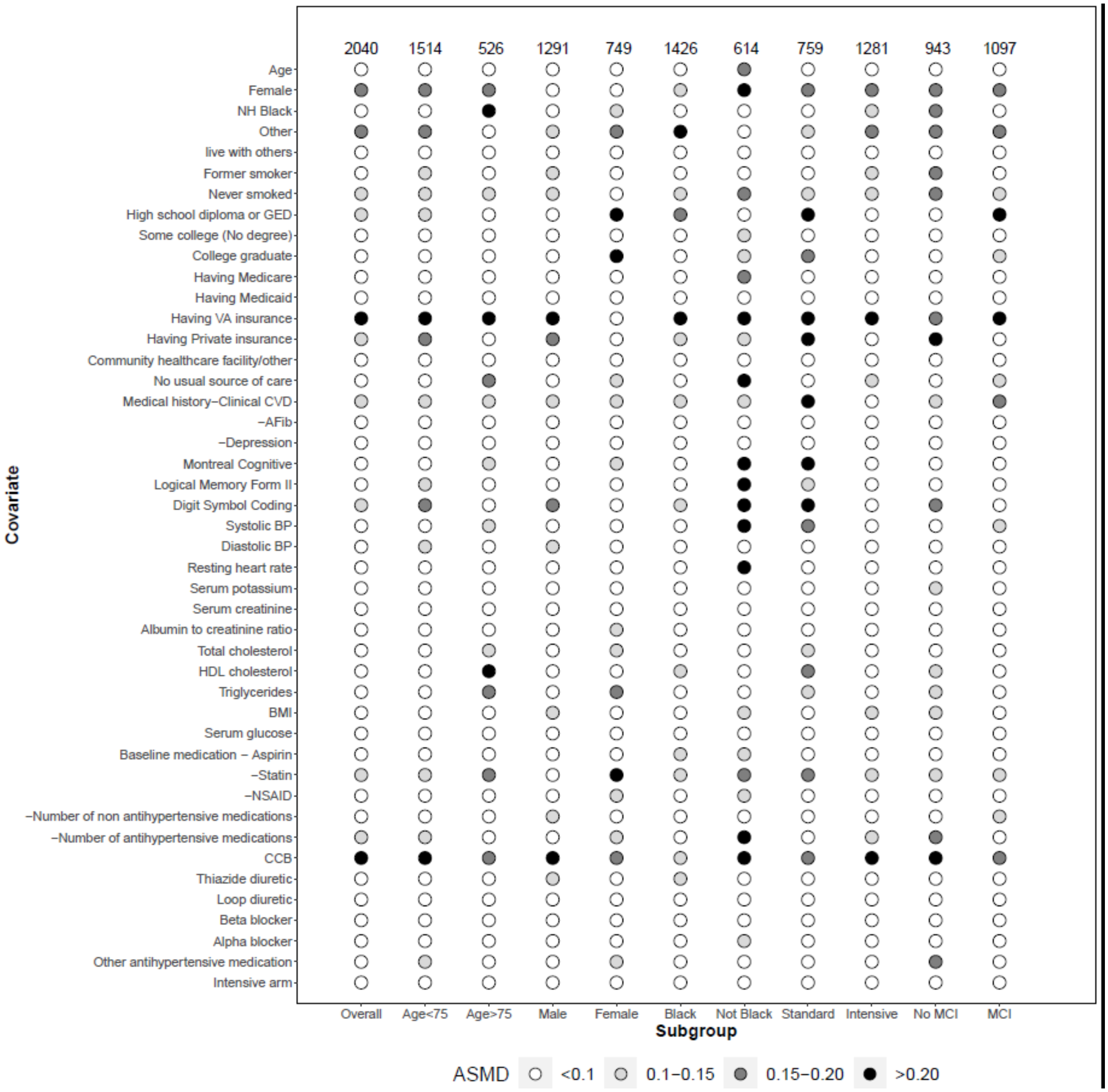

ASMDs <0.1 indicate that the weighted exposure groups have adequately small differences between measured baseline characteristics and therefore may be appropriate for inferential analysis. *Abbreviations:* AFib: atrial fibrillation; ASMD: absolute standardized mean difference; BP: blood pressure; BMI: blood pressure; CCB: calcium channel blocker; CVD: cardiovascular disease; GED: general educational development; IP: inverse probability; NH: Non-Hispanic; NSAID: non-steroidal anti-inflammatory drug

© 2022 Cohen JB et al. *JAMA Network Open.*

eFigure 7. Balance of pre-treatment characteristics between new users of an ARB vs. ACEI *after* inverse probability weighting overall and within subgroups.

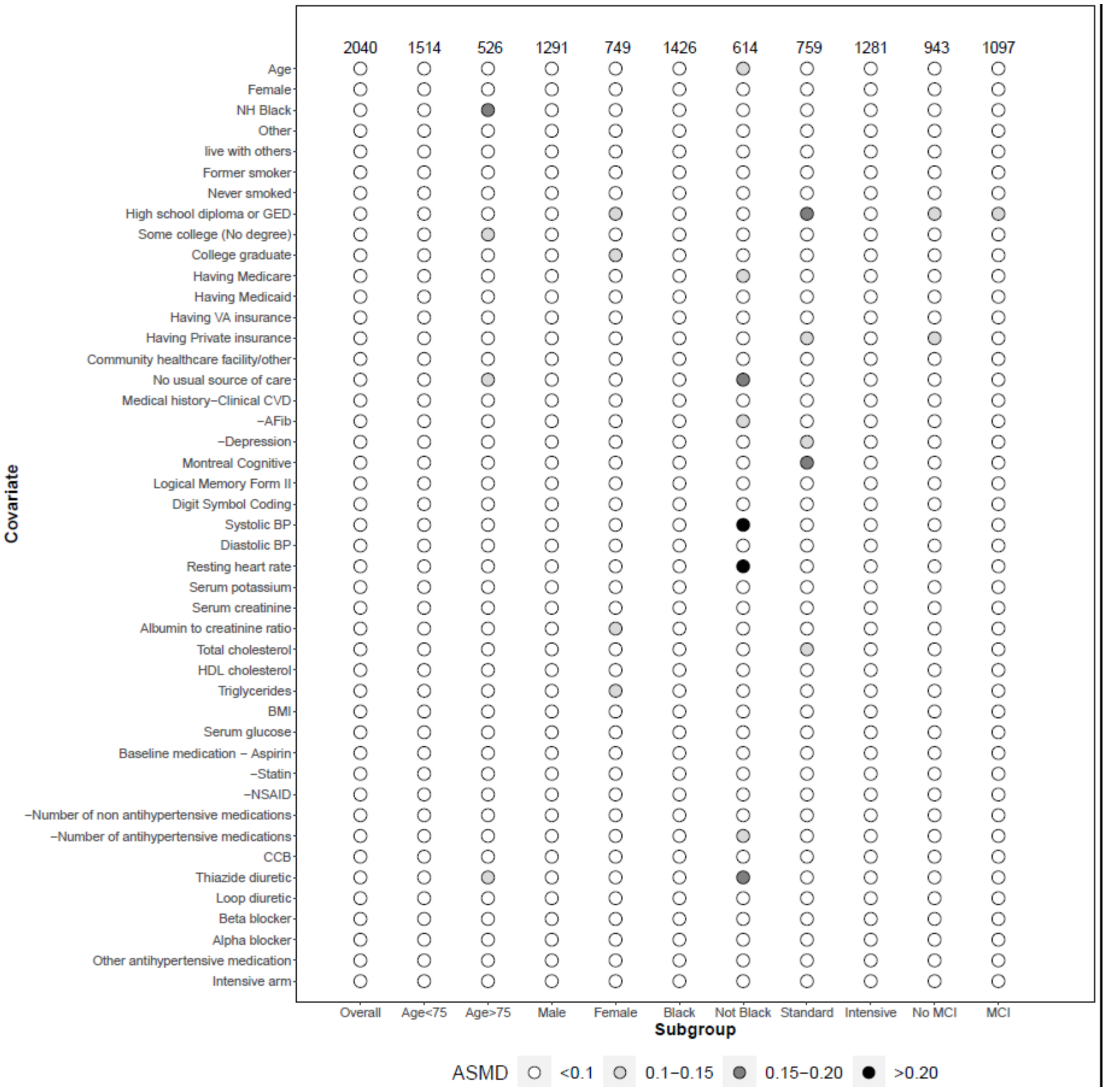

ASMDs <0.1 indicate that the weighted exposure groups have adequately small differences between measured baseline characteristics and therefore may be appropriate for inferential analysis. *Abbreviations:* AFib: atrial fibrillation; ASMD: absolute standardized mean difference; BP: blood pressure; BMI: blood pressure; CCB: calcium channel blocker; CVD: cardiovascular disease; GED: general educational development; IP: inverse probability; NH: Non-Hispanic; NSAID: non-steroidal anti-inflammatory drug

© 2022 Cohen JB et al. *JAMA Network Open.*

eFigure 8. Distribution of propensity scores among ARB vs. ACEI new users.

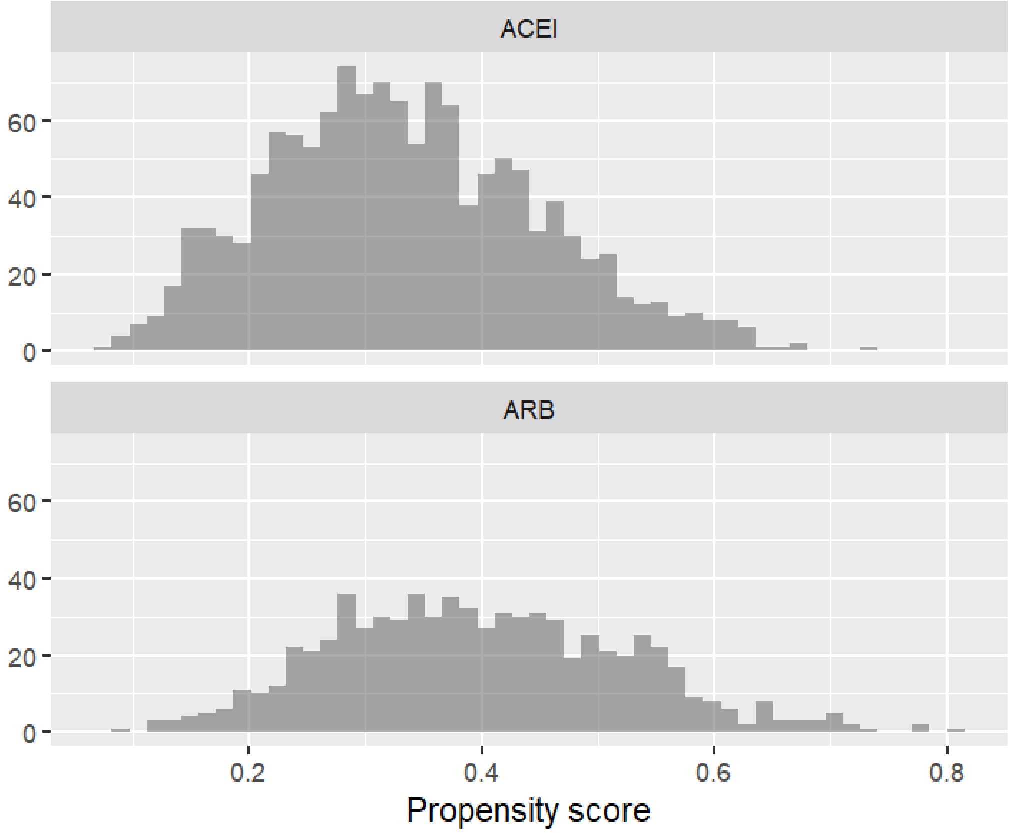

Abbreviations: ACEI: angiotensin-converting enzyme inhibitor; ARB: angiotensin II receptor blocker

© 2022 Cohen JB et al. *JAMA Network Open.*

eFigure 9. Follow-up systolic blood pressure among new users of an ARB and ACEI (unweighted).

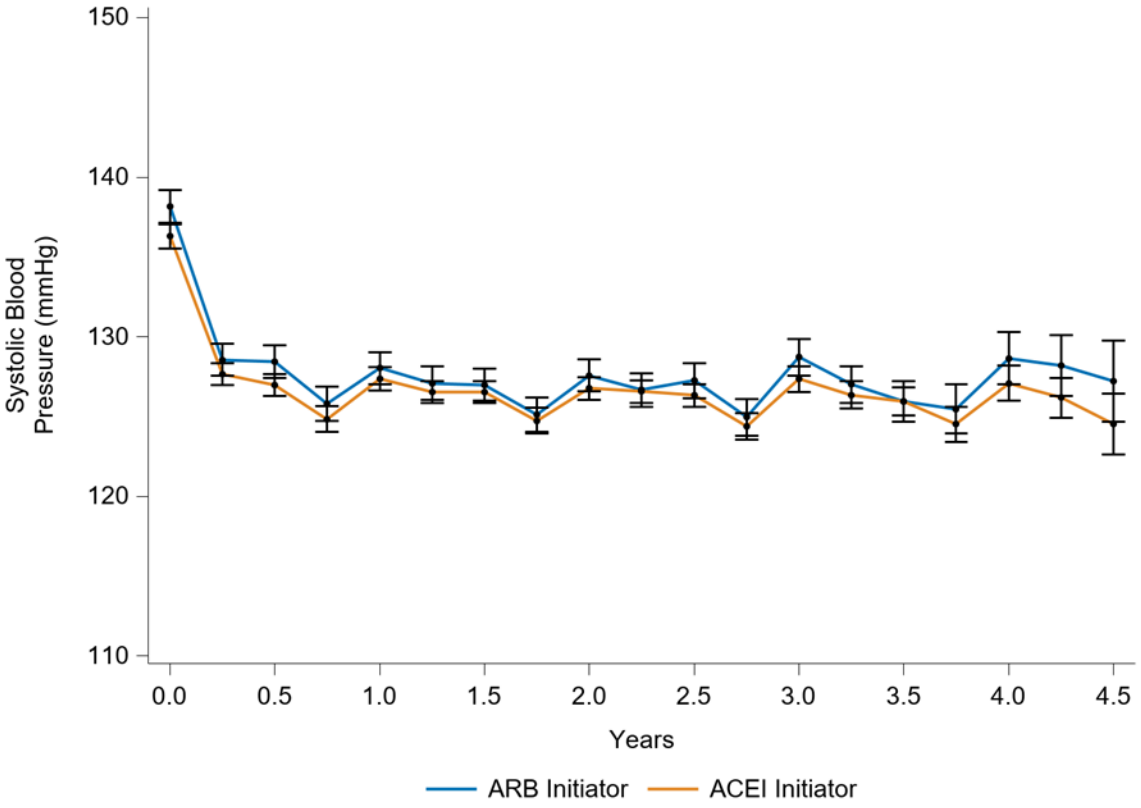

Mean No. of Medications

ARB Initiator	2.3	2.5	2.5	2.5	2.5	2.5	2.5	2.4	2.4	2.6
ACEI Initiator	2.2	2.1	2.1	2.1	2.1	2.2	2.2	2.2	2.2	2.4

Abbreviations: ACEI: angiotensin-converting enzyme inhibitor; ARB: angiotensin II receptor blocker

© 2022 Cohen JB et al. *JAMA Network Open.*

eFigure 10. IP-weighted cumulative incidence curves for secondary outcomes.

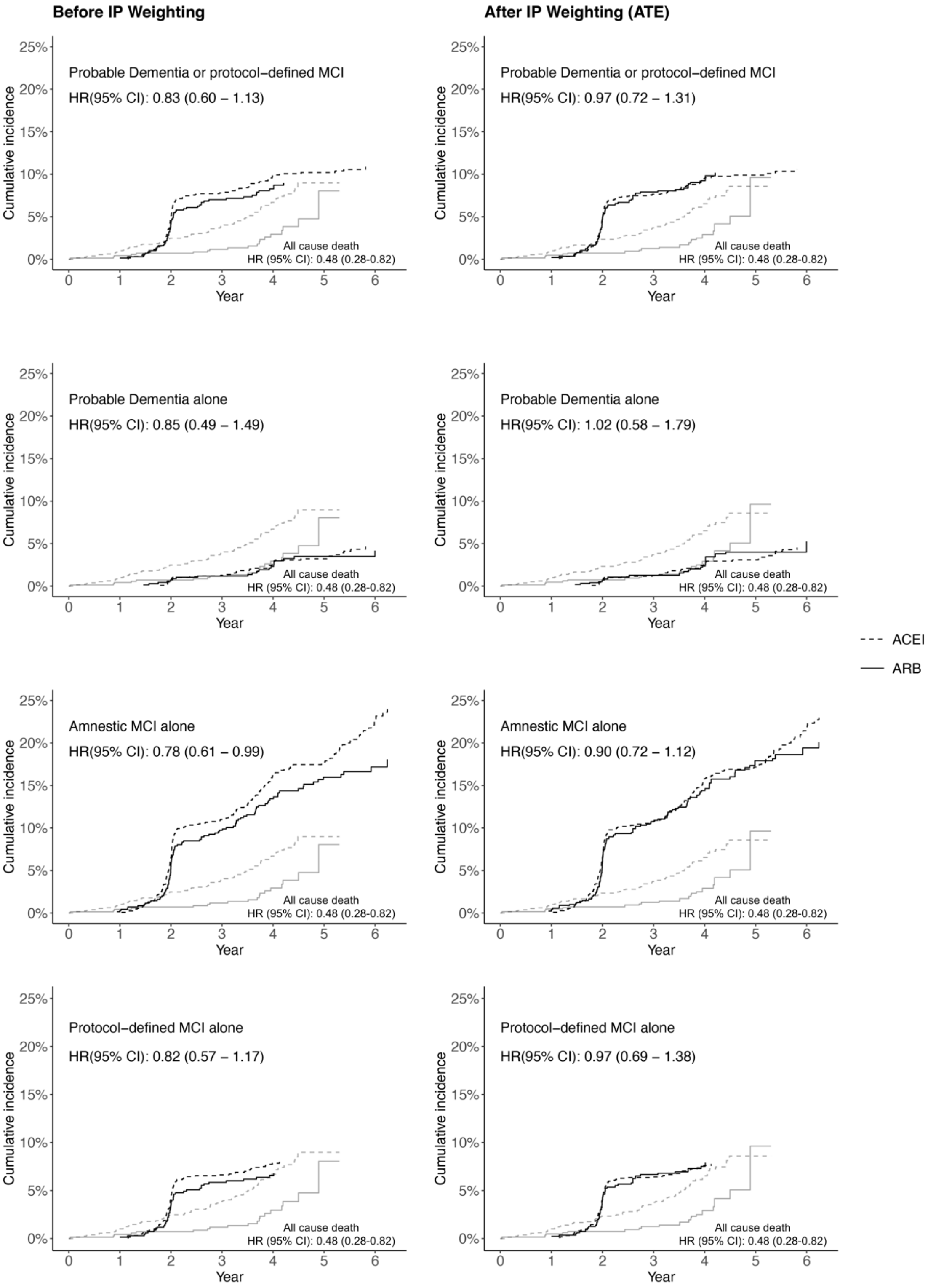

Abbreviations: ACEI: angiotensin-converting enzyme inhibitor; ARB: angiotensin II receptor blocker; CI: confidence interval; HR: hazard ratio; MCI: mild cognitive impairment

© 2022 Cohen JB et al. *JAMA Network Open.*

eFigure 11. Balance of pretreatment characteristics between new users of an ARB vs. ACEI *after* overlap weighting overall and within subgroups.

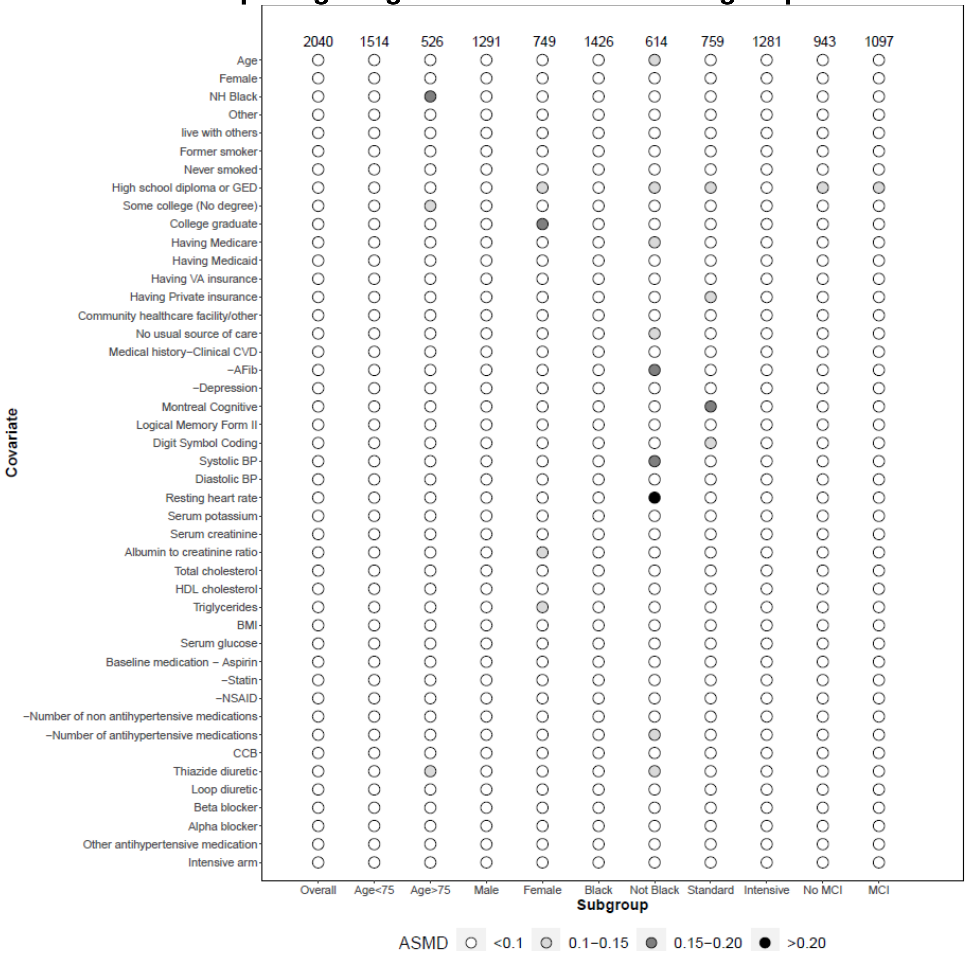

ASMDs <0.1 indicate that the weighted exposure groups have adequately small differences between measured baseline characteristics and therefore may be appropriate for inferential analysis. *Abbreviations:* AFib: atrial fibrillation; ASMD: absolute standardized mean difference; BP: blood pressure; BMI: blood pressure; CCB: calcium channel blocker; CVD: cardiovascular disease; GED: general educational development; IP: inverse probability; NH: Non-Hispanic; NSAID: non-steroidal anti-inflammatory drug

© 2022 Cohen JB et al. *JAMA Network Open.*

eFigure 12. Overlap-weighted cumulative incidence curves for secondary outcomes.

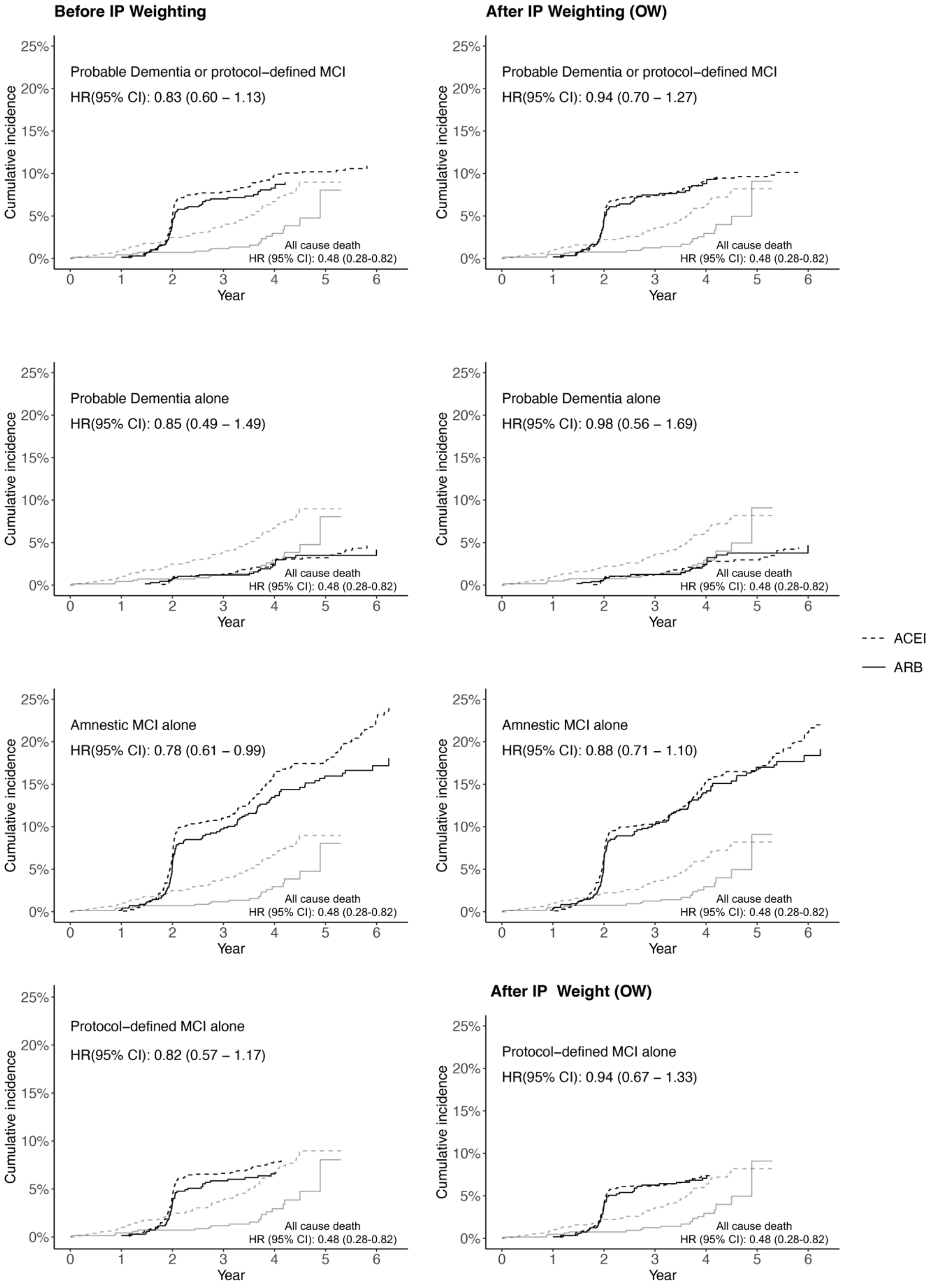

© 2022 Cohen JB et al. *JAMA Network Open.*

eTable 1. Components of the SPRINT Cognitive Battery

	Components of In-Person Cognitive Screening Battery	Components of In-Person Cognitive Extended Battery	Components of Telephone Cognitive Battery
Global Functioning	Montreal Cognitive Assessment		Modified Telephone Interview for Cognitive Status
Executive Function	Digit Symbol Coding Test		
Speed of Processing		Trail Making Test Parts A and B	Oral Trail Making Test Parts A and B
Learning and Memory	Logical Memory I	Hopkins Verbal Learning Test-Revised	
Visual-Spatial Memory		Modified Rey-Osterreith Complex Figure	
Working Memory and Attention		Digit Span Forward and Backward	
Verbal Fluency		Category Fluency-Animals	Category Fluency-Animals
Language and Naming		Boston Naming Test	

© 2022 Cohen JB et al. *JAMA Network Open.*

eTable 2. Baseline characteristics of new users of an ARB vs. ACEI before weighting using all available observed data.

Variable	N (%) with missing data	ARB New users (N=727)	ACEI New users (N =1,313)
Demographics			
Age (years), mean (SD)	0 (0%)	67.2 (9.7)	67.2 (9.4)
Female (%)	0 (0%)	308 (42.4%)	441 (33.6%)
Race/Ethnicity (%)	0 (0%)		
Non-Hispanic White		383 (52.7%)	817 (62.2%)
Non-Hispanic Black		240 (33%)	374 (28.5%)
Hispanic		89 (12.2%)	100 (7.6%)
Other		15 (2.1%)	22 (1.7%)
Social and Behavioral (%)			
Lives with others	1 (0.05%)	501 (69%)	888 (67.6%)
Current smoker	1 (0.05%)	107 (14.7%)	220 (16.8%)
Former smoker		271 (37.3%)	550 (41.9%)
Never smoker		349 (48%)	542 (41.3%)
Education (%)	0 (0%)		
Less than high school		68 (9.4%)	135 (10.3%)
High school graduate only		96 (13.2%)	231 (17.6%)
Post high school graduate		260 (35.8%)	446 (34%)
College graduate or greater		303 (41.7%)	501 (38.2%)
Health insurance status (%)	0 (0%)		
Uninsured		113 (15.5%)	193 (14.7%)
Medicare		373 (51.3%)	680 (51.8%)
Medicaid		49 (6.7%)	89 (6.8%)
VA		88 (12.1%)	270 (20.6%)
Private		340 (46.8%)	529 (40.3%)
Usual Source of Care (%)	3 (0.15%)		
Doctors' office/outpatient clinic		601 (82.9%)	1057 (80.6%)
Community healthcare facility/other		82 (11.3%)	146 (11.1%)
No usual source of care		42 (5.8%)	109 (8.3%)
Medical History (%)			
Clinical CVD	0 (0%)	77 (10.6%)	200 (15.2%)
History of atrial fibrillation	4 (0.2%)	54 (7.4%)	92 (7%)
History of depression	2 (0.1%)	138 (19%)	246 (18.8%)
Baseline cognitive assessments			
MoCA[a], median (IQR)	9 (0.4%)	24.0 (21.0, 26.0)	23.0 (20.0, 26.0)

© 2022 Cohen JB et al. *JAMA Network Open.*

Logical Memory form II[b], median (IQR)	9 (0.4%)	9.0 (6.0, 11.0)	8.0 (6.0, 11.0)
Digit Symbol Coding Test[c], median (IQR)	13 (0.6%)	53.0 (43.0, 62.0)	51.0 (41.0, 61.0)
Clinical/Laboratory Measurements			
Systolic BP, mm Hg (SD)	0 (0%)	143.5 (15.5)	142.1 (15.5)
Diastolic BP, mm Hg (SD)	0 (0%)	80.0 (12.3)	79.2 (12.4)
Resting heart rate, beats/minute (SD)	1 (0.05%)	66.9 (12.1)	67.5 (11.8)
Serum potassium, mg/dL (SD)	7 (0.3%)	4.1 (3.8, 4.4)	4.1 (3.8, 4.4)
Serum creatinine, mg/dL (SD)	4 (0.2%)	0.9 (0.8, 1.1)	1.0 (0.8, 1.1)
Albumin to creatinine ratio, mg/g (SD)	89 (4.4%)	9.7 (5.9, 21.4)	9.5 (5.8, 23.5)
Total cholesterol, mg/dL (SD)	4 (0.2%)	196.0 (170.0, 225.0)	193.0 (166.0, 223.0)
HDL cholesterol, mg/dL (SD)	4 (0.2%)	52.0 (44.0, 63.0)	51.0 (43.0, 61.0)
Triglycerides, mg/dL (SD)	4 (0.2%)	108.0 (77.0, 149.8)	108.0 (78.0, 157.0)
Body mass index, kg/m^2 (SD)	11 (0.5%)	29.1 (25.8, 32.7)	28.6 (25.4, 32.5)
Serum glucose, mg/dL (SD)	4 (0.2%)	96.0 (90.0, 105.8)	97.0 (90.0, 105.0)
Estimated glomerular filtration rate, mL/min^2 , mean (SD)	3 (0.15%)	75.5 (21.7)	75.7 (21.3)
Medication Use (%)			
Aspirin	0 (0%)	330 (45.4%)	639 (48.7%)
Statin	0 (0%)	216 (29.7%)	475 (36.2%)
NSAID	0 (0%)	244 (33.6%)	444 (33.8%)
Number of non-antihypertensive medications, mean (SD)	0 (0%)	2.0 (1.0, 5.0)	3.0 (1.0, 5.0)
Number of antihypertensive medications, mean (SD)	0 (0%)	2.0 (2.0, 3.0)	2.0 (2.0, 3.0)
CCB (%)	0 (0%)	300 (41.3%)	414 (31.5%)
Thiazide diuretic (%)	0 (0%)	337 (46.4%)	656 (50%)
Loop diuretic (%)	0 (0%)	36 (5%)	62 (4.7%)
Beta-blocker (%)	0 (0%)	237 (32.6%)	417 (31.8%)
Alpha-blocker (%)	0 (0%)	33 (4.5%)	58 (4.4%)
Other antihypertensive class (%)	0 (0%)	59 (8.1%)	77 (5.9%)
Intensive Treatment Arm (%)	0 (0%)	453 (62.3%)	828 (63.1%)

[a]Scores range from 0 to 30, with higher scores denoting better cognitive function.
[b]Subtest of the Wechsler Memory Scale. Scores range from 0 to 14, with higher scores denoting better cognitive function.
[c]Subtest of the Wechsler Adult Intelligence Scale. Scores range from 0 to 135, with higher scores denoting better cognitive function.

ACEI: angiotensin-converting enzyme inhibitor; ARB: angiotensin-II receptor blocker; ASMD: absolute standardized mean difference; BP: blood pressure; CCB: calcium channel blocker; CVD: cardiovascular disease; HDL: high-density lipoprotein; IQR: interquartile range; MoCA: Montreal Cognitive Assessment; NSAID: non-steroidal anti-inflammatory drug; SD: standard deviation; SPRINT: Systolic Blood Pressure Intervention Trial

© 2022 Cohen JB et al. *JAMA Network Open.*

eTable 3. IP-weighted serious adverse events among ARB vs. ACEI new users.

	ARB		ACEI		IP-weighted Hazard Ratio
	No. of events	IP-Weighted Event Rate*	No. of events	IP-Weighted Event Rate*	(95% CI)
**Outcome**					
Any SAE	39	1.7	87	2	0.84 (0.56,1.20)
**Conditions of interest**					
Serious adverse events only					
Hypotension	5	0.2	26	0.6	0.40 (0.09,0.94)
Syncope	9	0.4	20	0.5	0.96 (0.34,2.01)
Bradycardia	3	0.1	15	0.3	0.32 (0.00,0.85)
Electrolyte abnormality	7	0.3	24	0.5	0.53 (0.15,1.22)
AKI or ARF	12	0.5	24	0.5	0.89 (0.38,1.78)
ED visits or serious adverse events					
Hypotension	11	0.5	38	0.8	0.61 (0.26,1.14)
Syncope	11	0.5	26	0.6	0.83 (0.34,1.61)
Bradycardia	3	0.1	15	0.3	0.32 (0.00,0.85)
Electrolyte abnormality	11	0.5	29	0.7	0.75 (0.30,1.49)
AKI or ARF	12	0.5	27	0.6	0.80 (0.34,1.59)
**Monitored clinical events**					
Adverse lab measures					
Serum sodium <130 mmol/liter	27	1.1	48	1.1	1.04 (0.62,1.68)
Serum potassium <3.0 mmol/liter	20	0.8	31	0.7	1.15 (0.60,2.05)
Serum potassium >5.5 mmol/liter	30	1.3	55	1.3	1.00 (0.62,1.57)
Orthostatic hypotension					
Orthostatic Hypotension	129	5.3	262	6	0.90 (0.72,1.12)
Orthostatic Hypotension + dizziness	8	0.3	17	0.4	0.81 (0.26,1.89)

ACEI: angiotensin-converting enzyme inhibitor; AKI: acute kidney injury; ARF: acute renal failure; ARB: angiotensin-II receptor blocker; CI: confidence interval; ED: emergency department; IP: inverse probability; SAE: serious adverse events
*Per 100 person-years

© 2022 Cohen JB et al. *JAMA Network Open.*

eTable 4. IP-weighted primary outcome results in subgroups among ARB vs. ACEI new-users.

	ARB		ACEI		IP-Weighted Hazard Ratio (95% CI)	P value-interaction
**Subgroup**	**No. of events**	**IP-Weighted Event rate***	**No. of events**	**IP-Weighted Event rate***		
**Age**						
<75 years	64	3.0	142	3.1	0.98 (0.73,1.31)	0.38
≥75 years	54	8.2	118	9.8	0.81 (0.58,1.12)	
**Sex**						
Male	71	4.5	184	5.1	0.90 (0.68,1.18)	0.66
Female	47	3.9	76	3.9	0.99 (0.70,1.42)	
**Race-ethnicity**						
Black	50	5.8	79	5.2	1.11 (0.78,1.59)	0.22
Not Black	68	3.7	181	4.4	0.84 (0.65,1.10)	
**Treatment group**						
Standard arm	37	3.3	107	5.3	0.61 (0.41,0.91)	0.007
Intensive arm	81	4.8	153	4.2	1.17 (0.90,1.52)	
**Baseline MCI^**						
Absent	16	1.0	39	1.4	0.75 (0.40,1.42)	0.46
Present	102	7.0	221	7.9	0.97 (0.77,1.23)	

ACEI: angiotensin-converting enzyme inhibitor; ARB: angiotensin-II receptor blocker; CI: confidence interval; CVD: cardiovascular disease; IP: Inverse probability; MCI: mild cognitive impairment

*Per 100 person-years

^ Unadjudicated. Based on race-and education-specific Montreal Cognitive Assessment thresholds.[5]

© 2022 Cohen JB et al. *JAMA Network Open.*

eTable 5: Incidence rates and hazard ratios for the primary and secondary outcomes among new-users of an ARB vs. ACEI, unweighted and overlap-weighted

Outcomes	# Events ACEI	# Events ARB	Unweighted			Overlap weights		
			Rate ACEI (per 100 person years)[a]	Rate ARB (per 100 person years)[a]	HR (95% CI)[a]	Rate ACEI (per 100 person years)[b]	Rate ARB (per 100 person years)[b]	HR (95% CI)[b]
Probable Dementia or amnestic MCI (censoring death)	260	118	4.8	3.9	0.80 (0.64,1.01)	4.5	4.1	0.92 (0.75,1.11)
Probable Dementia or protocol-defined MCI	124	57	2.2	1.8	0.83 (0.60,1.13)	2.1	2	0.94 (0.70,1.27)
Probable Dementia alone	41	20	0.7	0.6	0.85 (0.49,1.49)	0.7	0.7	0.98 (0.56,1.69)
Amnestic MCI alone	233	103	4.4	3.5	0.78 (0.61,0.99)	4.2	3.7	0.88 (0.71,1.10)
Protocol-defined MCI alone	96	44	1.8	1.4	0.82 (0.57,1.17)	1.7	1.6	0.94 (0.67,1.33)
Probable Dementia or amnestic MCI or death	323	134	5.9	4.4	0.74 (0.60,0.91)	5.5	4.6	0.84 (0.71,1.01)
Probable Dementia or protocol-defined MCI or death	196	74	3.5	2.4	0.68 (0.52,0.89)	3.3	2.5	0.78 (0.60,1.01)
Probable Dementia or death	115	37	2	1.1	0.58 (0.39,0.84)	1.8	1.2	0.66 (0.46,0.97)
Amnestic MCI or death	298	120	5.5	3.9	0.72 (0.58,0.89)	5.1	4.1	0.82 (0.67,1.00)
Protocol-defined MCI or death	170	62	3	2	0.66 (0.49,0.89)	2.8	2.1	0.76 (0.57,1.01)
Death	78	19	1.6	0.7	0.43 (0.25,0.73)	1.5	0.8	0.49 (0.28,0.83)

ACEI: angiotensin-converting enzyme inhibitor; ARB: angiotensin-II receptor blocker; CI: confidence interval; HR: hazard ratio; MCI: mild cognitive impairment

[a]Unweighted [b]Overlap weights
95% CI were constructed using standard errors from 2500 bootstrap samples, assuming normal distribution.

© 2022 Cohen JB et al. *JAMA Network Open.*

eTable 6. Overlap-weighted serious adverse events among ARB vs. ACEI new users.

	ARB		ACEI		Overlap weighted Hazard Ratio
	No. of events	Overlap-Weighted Event Rate*	No. of events	Overlap-Weighted Event Rate*	(95% CI)
**Outcome**					
Any SAE	39	1.7	87	2	0.82 (0.56,1.18)
**Conditions of interest**					
Serious adverse events only					
Hypotension	5	0.2	26	0.5	0.42 (0.09,0.99)
Syncope	9	0.4	20	0.5	0.86 (0.32,1.89)
Bradycardia	3	0.1	15	0.4	0.34 (0.00,0.92)
Electrolyte abnormality	7	0.3	24	0.5	0.52 (0.17,1.18)
0.89 (0.38,1.78)AKI or ARF	12	0.5	24	0.5	0.90 (0.39,1.78)
ED visits or serious adverse events					
Hypotension	11	0.5	38	0.8	0.63 (0.28,1.19)
Syncope	11	0.5	26	0.6	0.76 (0.32,1.50)
Bradycardia	3	0.1	15	0.4	0.34 (0.00,0.92)
Electrolyte abnormality	11	0.5	29	0.7	0.70 (0.29,1.36)
0.80 (0.34,1.59)AKI or ARF	12	0.5	27	0.6	0.82 (0.35,1.60)
**Monitored clinical events**					
Adverse lab measures					
Serum sodium <130 mmol/liter	27	1.1	48	1.1	1.06 (0.63,1.72)
1.15 (0.60,2.05)Serum potassium <3.0 mmol/liter	20	0.8	31	0.7	1.10 (0.58,1.98)
Serum potassium >5.5 mmol/liter	30	1.3	55	1.3	0.99 (0.63,1.55)
Orthostatic hypotension					
Orthostatic Hypotension	129	5.3	262	6	0.89 (0.72,1.11)
Orthostatic Hypotension + dizziness	8	0.3	17	0.4	0.83 (0.28,1.97)

ACEI: angiotensin-converting enzyme inhibitor; AKI: acute kidney injury; ARF: acute renal failure; ARB: angiotensin-II receptor blocker; CI: confidence interval; ED: emergency department; IP: inverse probability; SAE: serious adverse events
*Per 100 person-years

© 2022 Cohen JB et al. *JAMA Network Open.*

eTable 7. Overlap-weighted primary outcome results in subgroups among ARB vs. ACEI new-users.

Subgroup	ARB		ACEI		Overlap-Weighted Hazard Ratio (95% CI)	P value-interaction
	No. of events	Overlap-Weighted Event rate*	No. of events	Overlap-Weighted Event rate*		
**Age**						
<75 years	64	2.9	142	3.0	1.00 (0.75,1.33)	0.23
≥75 years	54	7.7	118	9.7	0.77 (0.55,1.06)	
**Sex**						
Male	71	4.3	184	4.9	0.87 (0.67,1.14)	0.56
Female	47	3.8	76	3.8	1.00 (0.70,1.43)	
**Race-ethnicity**						
Black	50	5.6	79	5.0	1.11 (0.77,1.60)	0.18
Not Black	68	3.5	181	4.3	0.82 (0.63,1.06)	
**Treatment group**						
Standard arm	37	3.3	107	5.2	0.61 (0.42,0.90)	0.007
Intensive arm	81	4.6	153	4.1	1.15 (0.89,1.49)	
**Baseline MCI^**						
Absent	16	1.0	39	1.4	0.77 (0.41,1.44)	0.53
Present	102	7.4	221	7.7	0.95 (0.75,1.20)	

ACEI: angiotensin-converting enzyme inhibitor; ARB: angiotensin-II receptor blocker; CI: confidence interval; CVD: cardiovascular disease; MCI: mild cognitive impairment
*Per 100 person-years
^ Unadjudicated. Based on race-and education-specific Montreal Cognitive Assessment thresholds.[5]

© 2022 Cohen JB et al. *JAMA Network Open.*

eTable 8. IP-weighted primary outcome results among ARB vs. ACEI new-users, varying the definition of the initiation window.

	ARB			ACEI			
**Initiator identification window**	**No. of Initiators**	**No. of events**	**IP-weighted event rate***	**No. of Initiators**	**No. of events**	**IP-weighted event Rate***	**Hazard Ratio (95% CI)**
First 12 months follow-up (primary analysis)	727	118	4.3	1312	260	4.6	0.93 (0.76–1.13)
First 24 months follow-up	822	132	4.4	1468	292	4.8	0.93 (0.77–1.11)

ACEI: angiotensin-converting enzyme inhibitor; ARB: angiotensin-II receptor blocker; CI: confidence interval; IP: Inverse probability
*Per 100 person-years

© 2022 Cohen JB et al. *JAMA Network Open.*

eTable 9. Negative control outcomes analysis.

Group	#Events ACEI	Event rate ACEI	#Events ARB	Event rate ARB	Hazard ratio (95% CI)	P value
Unweighted						
Overall	132	3.1	60	2.5	0.81 (0.59,1.10)	-
Age<75	79	2.4	33	1.8	0.77 (0.51,1.17)	0.76
Age≥75	53	5.4	27	4.5	0.83 (0.51,1.35)	-
Male	86	3	33	2.4	0.80 (0.53,1.20)	0.91
Female	46	3.2	27	2.6	0.83 (0.51,1.34)	-
Black	29	2.4	11	1.4	0.57 (0.27,1.18)	0.30
Non Black	103	3.4	49	3	0.90 (0.64,1.27)	-
Standard Arm	42	2.7	16	1.8	0.64 (0.35,1.17)	0.35
Intensive Arm	90	3.3	44	2.9	0.89 (0.62,1.28)	-
No MCI	52	2.6	24	2	0.75 (0.45,1.24)	0.65
MCI	80	3.5	36	3	0.86 (0.58,1.29)	-
IP weights						
Overall	132	2.4	60	2	0.84 (0.62,1.16)	-
Age<75	79	1.8	33	1.5	0.80 (0.52,1.24)	0.74
Age≥75	53	4.3	27	3.7	0.88 (0.53,1.46)	-
Male	86	2.4	33	2	0.85 (0.55,1.30)	0.94
Female	46	2.4	27	2.1	0.87 (0.54,1.40)	-
Black	29	2.1	11	1.2	0.57 (0.26,1.23)	0.29
Non Black	103	2.5	49	2.3	0.93 (0.65,1.32)	-
Standard Arm	42	2.1	16	1.3	0.61 (0.33,1.13)	0.20
Intensive Arm	90	2.5	44	2.5	0.97 (0.67,1.40)	-
No MCI	52	2	24	1.7	0.84 (0.50,1.41)	0.99
MCI	80	2.8	36	2.4	0.84 (0.56,1.28)	-

ACEI: angiotensin-converting enzyme inhibitor; ARB: angiotensin-II receptor blocker; CI: confidence interval; IP: Inverse probability
The negative control outcome was defined as a Composite of pneumonia, urinary tract infection, sepsis, cellulitis, knee arthroplasty, spinal fusion surgery, spinal laminectomy, osteoarthritis, pulmonary embolism, deep vein thrombosis, and gastrointestinal hemorrhage.
Event rates are expressed per 100 person-years.

© 2022 Cohen JB et al. *JAMA Network Open.*

eReferences

1. Lewis CE, Fine LJ, Beddhu S, et al. Final Report of a Trial of Intensive versus Standard Blood-Pressure Control. N Engl J Med 2021;384:1921-1930.

2. Williamson JD, Pajewski NM, Auchus AP, et al. Effect of Intensive vs Standard Blood Pressure Control on Probable Dementia: A Randomized Clinical Trial. JAMA 2019;321:553-561.

3. Ambrosius WT, Sink KM, Foy CG, et al. The design and rationale of a multicenter clinical trial comparing two strategies for control of systolic blood pressure: the Systolic Blood Pressure Intervention Trial (SPRINT). Clin Trials 2014;11:532-546.

4. Systolic Blood Pressure Intervention Trial (SPRINT) Protocol Version 4.0 [online]. Available at: https://www.sprinttrial.org/public/Protocol_Current.pdf. Accessed 10/18/2015.

5. Milani SA, Marsiske M, Cottler LB, Chen X, Striley CW. Optimal cutoffs for the Montreal Cognitive Assessment vary by race and ethnicity. Alzheimers Dement (Amst) 2018;10:773-781.

© 2022 Cohen JB et al. *JAMA Network Open.*
