# Supplementary material for: Risk of Mild Cognitive Impairment or Probable Dementia in New Users of Angiotensin II Receptor Blockers and Angiotensin-Converting Enzyme Inhibitors: A Secondary Analysis of Data From the Systolic Blood Pressure Intervention Trial (SPRINT)
Source: JAMA Netw Open. 2022 Jul 14;5(7):e2220680. doi: 10.1001/jamanetworkopen.2022.20680 (PMC9284332; doi:10.1001/jamanetworkopen.2022.20680)
Supplement: Supplement 2. — Trial Protocol [file jamanetwopen-e2220680-s002.pdf]

Systolic Blood Pressure Intervention Trial (SPRINT)

Protocol Version 4.0

November 1, 2012

Table of Contents

Executive Summary 1

Chapter 1 – Introduction and Background 3

Chapter 2 – Overview of Trial Design 12

Chapter 3 – Participant Selection 15

Chapter 4 – Intervention 23

Chapter 5 – Measurements and Follow-up 31

Chapter 6 – SPRINT MIND 38

Chapter 7 – Health-Related Quality of Life and Economic Analyses 45

Chapter 8 – Safety Monitoring and Reporting 51

Chapter 9 – Clinical Outcome Measures 55

Chapter 10 – Statistical Considerations 59

Chapter 11 – Data Management 67

Chapter 12 – Quality Control 70

Chapter 13 – Study Organization 76

References – 85

Appendices:

Appendix 1: Abbreviations Used
Appendix 2: Computational Details and Sensitivity Analyses for the CVD outcomes
Appendix 3: Computational Details and Sensitivity Analyses for the MIND outcomes
Appendix 4: SPRINT Organizational Chart
Appendix 5: Membership and Charges of the Committees and Subcommittees
Appendix 6: Participating Sites

SPRINT Protocol
Executive Summary

The Systolic Blood Pressure Intervention Trial (SPRINT) is a 2-arm, multicenter, randomized clinical trial designed to test whether a treatment program aimed at reducing systolic blood pressure (SBP) to a lower goal than currently recommended will reduce cardiovascular disease (CVD) risk. About 9250 participants with SBP ≥ 130 mm Hg and at least one additional CVD risk factor will be recruited at approximately 90 clinics within 5 clinical center networks (CCNs) over a 2-year period, and will be followed for 4-6 years. Approximately 4300 participants will have chronic kidney disease (CKD), and 3250 will be aged 75 or older. The primary outcome is the first occurrence of a myocardial infarction (MI), acute coronary syndrome (ACS), stroke, heart failure (HF), or CVD death. Secondary outcomes include all-cause mortality, decline in renal function or development of end stage renal disease (ESRD), dementia, decline in cognitive function, and small vessel cerebral ischemic disease.

Design

SPRINT will randomize about 9250 participants aged ≥ 50 years with SBP ≥130 mm Hg and at least one additional CVD risk factor. The trial will compare the effects of randomization to a treatment program of an intensive SBP goal with randomization to a treatment program of a standard goal. Target SBP goals are <120 vs <140 mm Hg, respectively, to create a minimum mean difference of 10 mm Hg between the two randomized groups. The primary endpoint is incident CVD events identified over a follow-up period of up to six years. The primary hypothesis is that CVD event rates will be lower in the intensive arm. Both the number of randomizations and the length of follow-up may differ from these targets depending on how observed values of parameters differ from estimates used to design the study. Secondary hypotheses include whether the lower SBP goal reduces CVD event rates and progression of renal disease in people with CKD, whether the lower SBP goal reduces progression of CVD event rates in people aged 75 or older, the impact of treatment strategy on health-related quality of life (HRQL), and the relative cost-effectiveness of the two strategies. Investigation of relevant genetic pathways and other genetic analyses will also be conducted. The sample size of the trial will be enriched by including 4300 persons with CKD (estimated GFR 20-59 ml/min/1.73 m^2) to permit assessment of treatment effect on CVD in this subgroup, as well as on measures of progression of kidney disease. The trial will also include 3250 participants who are 75 years old or older. The SPRINT Memory and cognition IN Decreased hypertension (SPRINT MIND study) will test whether the lower SBP goal influences the rate of incident dementia and mild cognitive impairment, global and domain-specific cognitive function, and small vessel ischemic disease. The sample sizes for each of the three components of the MIND study are different. Incident dementia will be determined in all participants. The rate of non-dementia related cognitive decline in important domains of cognition will be measured in 2800 persons representative of all SPRINT participants and from these 2800 persons the magnetic resonance imaging (MRI) study will involve a sub-set of 640 participants.

Patient population

Although epidemiologic evidence strongly suggests that lowering SBP will reduce CVD risk in nearly all adults, for practical and public health reasons the hypothesis is most efficiently studied in persons with an elevated risk of CVD. Thus, the trial will recruit persons 50 years or older with SBP ≥130 mm Hg and at least one additional CVD risk factor. Three groups will be excluded – patients with diabetes, patients with polycystic kidney disease (PKD), and patients who have had a stroke – because they are the target groups of completed or ongoing trials that are testing a lower BP goal. SPRINT will focus on three high risk groups: patients with clinical CVD other than stroke, patients with chronic kidney disease (estimated glomerular filtration rate [eGFR] 20-59 mL/min/1.73 m^2), and patients who have a Framingham Risk Score (FRS) of ≥15%. A large subgroup will be participants who are 75 years old or older. This trial is expected to enroll 50% women and 40% who are members of minority groups (African Americans, Hispanics, Native Americans, and Asians)

Sample size and power

Based on the Antihypertensive and Lipid-Lowering Treatment to Prevent Heart Attack Trial (ALLHAT) event rates adjusted downward approximately 50% for temporal changes in CVD risk factors and improved therapy, a sample size of 9250 provides approximately 90% power to detect a 20% effect on the primary composite endpoint of CVD mortality and non-fatal MI, ACS, stroke, and heart failure. The annual event rate used in this calculation was 2.2%. Recruitment of a subgroup of 4300 participants with CKD provides 80% power to detect a 20% effect on the same CVD composite endpoint. The probable dementia component of the MIND study will provide 80% power to detect a 15% reduction in the incidence of dementia, 2800 SPRINT-MIND participants will provide ample power to detect a 20% reduction in the rate of decline in cognitive function between the two arms (more intensive vs. less intensive blood pressure control). In addition, MRI testing to detect differences in small vessel ischemic disease and total brain volume will provide 80% and 90% power, respectively, between the two strategy groups in SPRINT.

Other secondary outcomes

Several additional secondary outcomes will be examined, such as markers of renal function in non-CKD participants, co-morbidities, quality of life, and cost-effectiveness. Adverse events (e.g., postural hypotension, including falls) and biochemical changes will be measured and analyzed by randomized arm.

Chapter 1 – Introduction and Background

1. Background

1.1 Hypertension, public health and the need for a clinical trial testing a lower SBP target.

Elevated blood pressure (BP) is an important public health concern. It is highly prevalent, the prevalence may be increasing, and it is a risk factor for several adverse health outcomes, especially coronary heart disease, stroke, heart failure, chronic kidney disease, and decline in cognitive function. Given the high prevalence and severity of adverse outcomes, even small improvements in the treatment of elevated BP would result in widespread benefit. The benefit of lowering SBP to around 140 mm Hg is well-accepted, but patients treated to this level of BP are still at increased risk of BP-related adverse outcomes. Observational studies document a progressive increase in risk as BP rises above 115/75 mm Hg. Such epidemiologic evidence suggests there may be substantial benefit to targeting treatment to a SBP <120 mm Hg instead of <140 mm Hg. In contrast, targeting to <120 mm Hg may be harmful or unnecessarily costly and burdensome with limited expectation of benefit. Apart from the Action to Control Cardiovascular Risk in Diabetes (ACCORD) trial, which was restricted to participants with diabetes mellitus, no clinical trial has been conducted to test the hypothesis that more intensive reduction in SBP to <120 mm Hg is beneficial compared to the current recommendation of a goal SBP <140 mm Hg. At present, the results from clinical trials that have addressed related hypotheses are ambiguous. A definitive clinical trial testing whether lowering SBP below 120 mm Hg is better than lowering SBP below 140 mm Hg in non-diabetic hypertensive patients is needed, and this has been designated by an NIH Expert Panel as the most important hypothesis to test regarding the prevention of hypertension-related complications (2007).

1.1.1 Prevalence of hypertension

Approximately 1 billion people worldwide have hypertension (HTN) (Kearney and others, 2005). HTN is highly prevalent in the adult population of the US, especially among those aged ≥60 years. Two-thirds of those over age 60 have HTN, and the prevalence has increased in recent decades (Chobanian and others, 2003;Cutler and others, 2008;Hajjar and Kotchen, 2003;Ong and others, 2007;World Health Organization, 2002). By age 50 years, isolated systolic hypertension (ISH) is the most common form of HTN, and is associated with greatest risk of target organ damage and adverse health outcomes (Franklin, 1999;Franklin and others, 2001).

1.1.2 Hypertension as a cardiovascular risk factor

The importance of BP, especially SBP, as an independent risk factor for coronary events, stroke, chronic heart failure (CHF), and ESRD is well documented (Vasan and others, 2001;Collins and others, 1990;Macmahon and others, 1990;Sacco and others, 2001;Jackson, 2000;Staessen and others, 1997;Hsu and others, 2005;Chobanian and others, 2003;Gillum, 1991;Prospective Studies Collaboration, 2002;Levy and others, 1996). There is also substantial epidemiologic and clinical trial evidence supporting a role for hypertension therapy in reducing risk for age-related dementia, including vascular dementia and Alzheimer's dementia (Forette and others, 1998;Luchsinger and

Mayeux, 2004;Reitz and others, 2007;Skoog and Gustafson, 2003;Skoog and others, 2005;Skoog and Gustafson, 2006;Tzourio and others, 2003). Clinical trial data have shown reductions in CVD outcomes, including incident stroke (35% to 40%), MI (15% to 25%), and CHF (up to 50%) (Chobanian and others, 2003;Psaty and others, 1997;Neal, Macmahon, and Chapman, 2000). However, optimal targets for BP lowering are not established.

1.1.3 Support for current target

In addition to the Seventh Report of the Joint National Committee on Prevention, Detection, Evaluation, and Treatment of High Blood Pressure (JNC-7) (Chobanian and others, 2003), most recent practice guidelines recommend a target SBP <140 mm Hg in persons with established uncomplicated hypertension (Campbell and others, 2009;Mancia and others, 2007;Mancia and others, 2009;National Collaborating Centre for Chronic Conditions, 2006;National Heart Foundation of Australia (National Blood Pressure and Vascular Disease Advisory Committee), 2009;Whitworth, 2003). The benefits of lowering high BP in reducing CV morbidity and mortality are well-established (Cutler, MacMahon, and Furberg, 1989;Psaty and others, 1997). A meta-analysis evaluating the treatment efficacy of hypertension therapy in adults over age 60, from three major trials from different countries (Liu and others, 1998;SHEP, 1991;Staessen and others, 1997) found that lowering SBP significantly reduced all-cause and CVD mortality by 17% and 25% respectively, and all CVD end-points by 32% (Staessen and others, 1999;Staessen, Wang, and Thijs, 2001), though both treatment goals and the achieved SBP were >140 mm Hg.

1.1.4 Risk of SBP above normal but below current target

The World Health Organization estimates that about two-thirds of the cerebrovascular disease burden and one-half of the coronary heart disease (CHD) burden on a worldwide basis is attributable to SBP >115 mm Hg (World Health Organization, 2002). Further, SBP > 115 mm Hg has been estimated to account for 7.6 million premature deaths (13.5% of the global total), 92 million disability-adjusted life years (6.0% of the global total), 54% of stroke, and 47% of ischemic heart disease. About half of this burden is in persons with a SBP<145 mm Hg (Lawes, Vander, and Rodgers, 2008). The JNC-7 defined pre-hypertension based on the evidence that SBP values between 120 and 139 mm Hg and diastolic blood pressure (DBP) values between 80 and 89 mm Hg are associated with increased cardiovascular (CV) risk. Although the risk of a BP between 120/80 and 139/89 mm Hg is not as pronounced as that associated with a BP above 140/90 mm Hg (Chobanian and others, 2003), 36% of the adult US population had a BP within this range in the 2007-2008 National Health and Nutrition Examination Survey (Wang and Wang, 2004).

Strong evidence from large population-based longitudinal observational studies indicates that, regardless of other cardiovascular risk factors, SBP levels of about 115 mm Hg in adults over the age of 40 years are associated with lower CVD event rates, including death and slower progression of subclinical CVD (Lewington and others, 2002;Sipahi and others, 2006) compared to higher SBPs. In the Framingham Heart Study (FHS), the risk of CVD following 10 years of follow-up among persons with SBP 130-139 mm Hg and/or DBP 85-89 mm Hg and SBP 120-129 mm Hg and/or diastolic blood pressure (DBP) 80-84 mm Hg was significantly higher when compared to their counterparts with SBP <120 mm Hg and DBP <80 mm Hg (Vasan and others, 2001). Experience in the

Atherosclerosis Risk in Communities (ARIC) and Women's Health Initiative (WHI) studies also showed that individuals with SBP of 120-139 mm Hg and/or DBP of 80-89 mm Hg had an increased risk of CV events, relative to persons with SBP <120 mm Hg (Hsia and others, 2007;Kshirsagar and others, 2006). A large meta-analysis of data from 61 population-based longitudinal epidemiological studies showed a strong continuous graded relationship between SBP and CVD death risk for all age deciles between 40-89 years, independent of other CVD risk factors, beginning at SBP levels of about 115 mm Hg (Lewington and others, 2002). For those aged 40-69 years, there was an approximate doubling in the rates of death from stroke, ischemic heart disease and other vascular causes with each increase of 20 mm Hg in usual (that is, long-term average) SBP.

1.1.5 Evidence for possible benefit of lower target on CV outcomes

Clinical trial evidence of benefit from achieving SBP levels that approach the current recommended goal of <140 mm Hg with pharmacologic treatment is strong, but a trial specifically designed to test lowering the SBP treatment goal below the 140 mm Hg level, the ACCORD trial, found no clear evidence of benefit. The ACCORD trial tested the research question of whether a therapeutic strategy aimed at reducing SBP to <120 mm Hg was more effective in reducing CVD events than a strategy aimed at SBP <140 mm Hg in participants who had diabetes and were at increased risk for CVD events. ACCORD found a non-significant reduction in CV events in the intensively treated group, though a lower than expected event rate contributed to an inability to exclude a clinically meaningful effect (The ACCORD Study Group, 2010). The lack of overall benefit was generally consistent across a variety of subgroups. This is in contrast to prior experience of improved outcomes with more compared to less intensive BP reduction in the diabetic participants in the United Kingdom Prospective Diabetes Study (UKPDS) and in the diabetic subgroups in the Hypertension Optimal Treatment trial (HOT), Systolic Hypertension in the Elderly Program (SHEP) and Systolic Hypertension in Europe trial (Syst-Eur). Importantly, none of these trials tested the same level of intensity of BP reduction or the low BP goal employed in ACCORD. Consistent with previous trials, ACCORD did find a large reduction in the incidence of stroke in the intensively treated group, and though the incidence of serious adverse effects was significantly greater in the intensive treatment group, adverse events occurred with relatively low frequency overall.

Results from overall or subgroup analyses of other CV outcome trials are mixed, with some providing support for the benefit of a lower BP goal but others not providing such evidence. In addition, supportive data from other trials have generally been based on analyses of achieved BP rather than pre-defined treatment goals. For example, the Hypertension Detection and Follow-up Program (HDFP) showed reductions in mortality (17%) and CVD mortality (19%) in participants randomized to Stepped Care treatment of hypertension compared with Referred Care. Participants in the Stepped Care arm averaged 159 mm Hg at baseline and achieved SBP levels of 130 mm Hg at 4 years and 140 mm Hg at 5 years of follow-up (Abernethy and others, 1986;HDFP, 1979b;HDFP, 1979a;HDFP, 1982). In the Heart Outcomes Prevention Evaluation (HOPE) study, the use of ramipril in high-risk patients lowered SBP by 3-4 mm Hg from a baseline mean of 139 mm Hg compared to placebo and reduced the composite CVD endpoint that included CVD death (26%), MI (20%), stroke (32%), revascularization (15%), and CHF (23%) (Yusuf and others, 2000). In the European Trial on Reduction of Cardiac Events with Perindopril in Stable Coronary Artery Disease (EUROPA), use of perindopril (vs.

placebo) resulted in a 5/2 mm Hg reduction in BP (from a mean baseline value of 137/82 mm Hg) and a 20% reduction in CVD events (Fox, 2003). The perindopril protection against recurrent stroke study (PROGRESS) showed a significant reduction in stroke and major vascular events associated with a 9/4 mm Hg reduction in BP from a baseline mean of 147/86 mm Hg (PROGRESS Collaborative Group, 2001). More importantly, in a prespecified subgroup analysis, those receiving 2 drugs (perindopril plus indapamide) had greater reductions in BP (12/5 mm Hg) and risk (43%) compared with placebo versus those on perindopril alone compared with placebo (5/3 mm Hg and 5%), supporting the hypothesis that lower BP is better. There were similar reductions in the risk of stroke in hypertensive and non-hypertensive subgroups (all $p<0.01$). Finally, in the Comparisson of Amlodipine vs. Enalapril to limit Occurences of Thrombosis trial (CAMELOT), a placebo-controlled trial of patients with heart disease and DBP <100 mm Hg (mean 129/78 mm Hg), amlodipine decreased BP by 4.8/2.5 mm Hg and CVD events by 31% (hazard ratio [HR], 0.69; 95% CI, 0.54-0.88); whereas enalapril lowered BP by 4.9/2.4 mm Hg but did not decrease events (HR, 0.85; 95%CI 0.67-1.07) (Nissen and others, 2004).

Other trials have not supported the hypothesis of benefit from a lower SBP target. In the HOT study, there were no differences in CVD events between groups randomized to target DBPs of ≤90 mm Hg vs ≤85 mm Hg vs ≤80 mm Hg in the entire cohort of 18,790 hypertensive participants; the average on-treatment SBP levels were 140 mm Hg and 144 mm Hg, respectively, in the ≤80 and ≤90 mm Hg target groups (Hansson and others, 1998). Only a post hoc analysis of the diabetic subgroup (n=1,501) showed that major CVD events were reduced by 51% ($p=0.005$) in those randomized to the lower BP goal. The average on-treatment SBP levels were 140 mm Hg and 144 mm Hg in the ≤80 and ≤90 mm Hg target groups, respectively (Hansson and others, 1998). Likewise, there was no special benefit in those with an achieved SBP of 130 mm Hg vs. 134 mm Hg in the Prevention of Events with Angiotensin Converting Enzyme (PEACE) trial, which compared trandolapril treatment to placebo in persons with stable coronary artery disease (Braunwald and others, 2004). In the aggregate, these trials had only modest net reductions in SBP (4-6 mm Hg), though ACCORD and other trials have shown that a much larger reduction (14 mm Hg difference in SBP between the two arms) can be achieved.

The ACCORD BP results provide a strong rationale for testing the potential benefits of intensive BP lowering. (i) The confidence interval around ACCORD's non-significant effect does not exclude benefit in the range of 20% to 25% reduction in the rate of CV events. Effects of that magnitude would be of considerable importance to public health. (ii) Serious adverse effects were significantly more frequent in the intensive treatment group, but occurred with low frequency overall. (iii) People without diabetes, who are probably less prone to microvascular disease but were excluded from ACCORD, may benefit from more intensive BP lowering. (iv) ACCORD excluded people with serum creatinine levels >1.5 mg/dL, which are prevalent in the US population and associated with high CV risk. (v) the glycemia arm of the ACCORD trial was stopped early because of an excess in total mortality and the possibility of interaction between these two interventions is still under investigation. The safety and benefit of intensive BP reduction in patients > age 75 remain to be tested. Thus, it is imperative that the potential benefits and harms of intense SBP-lowering be examined definitively in this and other high-risk populations, e.g. those with chronic kidney disease (CKD) or underlying CVD.

1.1.6 Possible harm from treatment of SBP to <120 mm Hg

There are a number of reasons for requiring recommendations to lower SBP treatment goals be based on definitive trial evidence. Treating to lower BP levels with medications could be harmful. For example, one proposed mechanism that has some support in post hoc analyses of clinical trials (Cruickshank and others, 1987;Cruickshank, 2000;Somes, Shorr, and Pahor, 1999), known as the “J-curve” hypothesis, states that lowering DBP too much may decrease coronary artery perfusion and increase the risk of CVD events in patients with coronary artery disease (CAD). In post-hoc observational analyses of clinical trial experience, the level of DBP below which risk increased has varied by trial, sometimes being as high as <85 mm Hg (Cruickshank and others, 1987). In corresponding analyses of SHEP participants, the higher risk was reported with DBP <55-60 mm Hg during treatment (Somes and others, 1999).

Further, if treatment has little or no benefit, adding drugs is a waste of patients' and payers' resources and time. For example, in a cost-effectiveness analysis of the HOT trial, which overall did not show a significant benefit for lower DBP goals, the cost-effectiveness ratios, expressed as cost per year of life gained, were most favorable for the DBP ≤90 mm Hg target group ($4262) and for added aspirin treatment ($12,710) (HOT, 1998). In the moderately intensive treatment (DBP ≤85 mm Hg) group, the cost-effectiveness ratio escalated to $86,360; with intensive treatment (DBP ≤80 mm Hg), costs further increased to $658,370 per year of life gained. Only treatment to a DBP target of 90 mm Hg and co-administering aspirin were considered highly cost effective; intensive BP lowering down to 80 mm Hg was clearly very costly.

A third reason for not recommending lower SBP goals without definitive clinical trial evidence relates to the increased number of drugs required to achieve these goals. For example, in the African American Study of Kidney disease and hypertension (AASK) trial, the intensive BP goal (achieved SBP = 128 mm Hg) group required an average of 3.04 drug classes compared with 2.39 in the conventional BP goal group (Wright, Jr. and others, 2002a) and in the ACCORD BP trial experience >3 drug classes were required for the intensive SBP goal group to achieve a SBP average of 119 mm Hg, compared with 2 classes in the standard SBP goal group with a mean SBP achieved of 134 mm Hg (The ACCORD Study Group, 2010). In addition to being more costly and having greater potential for drug-related adverse events, even 1-2 more medications per day may contribute to reduced adherence to other evidence-based drug treatment (e.g., statins or aspirin). Patients may choose to not take medications without more evidence for safety and benefit. In addition to being more costly, burdensome, and potentially risky, a 20-mm Hg lower SBP goal (and/or a 10 mm Hg lower DBP goal) would likely mean that up to 70-80 million Americans now considered "prehypertensive" may require drug therapy for a condition that has not been proven to be benefited by treatment (Greenlund, Croft, and Mensah, 2004).

Finally, all medications carry an intrinsic risk of side effects which may adversely affect clinical outcomes and quality of life, and lead to drug interactions, especially in older persons who may need to take a variety of medications.

1.1.7 Conclusion

If the SPRINT results are positive and support a SBP goal <120 mm Hg, and this is fully applied in practice a large number of major CVD could be prevented each year, in the

U.S. alone. If the results are negative and SPRINT is sufficiently powered and well-conducted, then recommendations for SBP goal in the treatment of most hypertensive patients, including those with stage 3 CKD and pre-existing CVD, would 1) allow for a redoubled focus on achieving a SBP goal of <140 mm Hg, and 2) abrogate the need for the additional effort and cost of achieving a lower SBP goal than currently recommended for most patients with elevated BP. If none of the major outcomes show harm from lowering to <120, and if any of the outcomes are positive, SPRINT may make a substantial contribution to public health.

1.2 SPRINT's target patient population

Although epidemiologic evidence strongly suggests that lowering SBP will reduce CVD risk in nearly all adults, for practical and public health reasons the hypothesis is most efficiently studied in high-risk individuals. A high risk population stands to benefit most in the sense that a greater number of events may be prevented per treated individual. Furthermore, results in a diverse high risk population will likely generalize to lower risk populations, at least in terms of relative risk reduction. Thus, the SPRINT trial will recruit patients 50 years or older with SBP $\geq$130 mm Hg who either have or are at high risk for CVD. SPRINT will focus on three high risk groups: individuals with clinical CVD other than stroke, individuals with CKD (estimated glomerular filtration rate [eGFR] 20-59 ml/min/1.73 m^2), and individuals without clinical CVD who have high estimated CVD risk based on factors such as smoking, low levels of HDL, high levels of LDL or age. Three other groups will be excluded: patients with diabetes, patients with polycystic kidney disease (PKD), and patients who have had a stroke. Patients with diabetes have been studied in the ACCORD trial; patients with prior stroke and PKD are part of other ongoing trials.

1.2.1 Chronic Kidney Disease (CKD)

An important and under-studied high-risk group for CVD is the population with CKD (Coca and others, 2006). In the U.S., the number of persons with Stage 3 CKD (eGFR between 30 and 60 ml/min/1.73 m^2) has recently been estimated to be 7.7% of the adult population, or 15.5 million (Coresh and others, 2007). Patients with prevalent CVD have a high prevalence of CKD, with reported ranges of 30-60% (Keeley and others, 2003;Levey and others, 1998;Shlipak and others, 2002).

Individuals with CKD are at high risk for CVD events (Shlipak and others, 2009;Go and others, 2004;Rahman and others, 2006;Weiner and others, 2004;Foster and others, 2007;McCullough and others, 2007;Rashidi and others, 2008;Fried and others, 2009). A meta-analysis of reported data from prospective studies in Western populations demonstrated that people with an eGFR of <60 ml/min/1.73m^2 have a relative risk of 1.4 for CVD, compared to those with an eGFR of $\geq$60 ml/min/1.73m^2 (Di Angelantonio and others, 2007). The relative risk increases as eGFR declines (Go and others, 2004). Pooled data from the ARIC and CHS cohorts demonstrated that participants with CKD were also at increased risk for stroke (Weiner and others, 2007), and CKD was a risk factor for CVD and all-cause mortality independent of traditional CVD risk factors (Weiner and others, 2004). In ALLHAT, despite exclusion criteria designed to exclude participants with significant GFR impairment, about 18% of participants had an eGFR 30-60 ml/min/1.73m^2. In that CKD subgroup, CHD was 38% higher and combined CVD 35% higher than in those with an eGFR >90 ml/min/1.73m^2 (Rahman and others, 2006).

The effect of BP control on the development of CVD in the CKD population is far less clear (Berl and others, 2005).

A strategy of treating to a lower BP goal may reduce the progression of kidney disease. The risk of CKD increases progressively beginning with pre-HTN levels of BP through the various stages of HTN (Haroun and others, 2003). Several observational studies have suggested that achievement of lower BP is associated with lower risk of adverse kidney outcomes (Bakris and others, 2000;Klag and others, 1996;Schaeffner and others, 2008). However, two randomized clinical trials, AASK and the Modification of Diet in Renal Disease Study (MDRD) that examined lower-than-usual BP goals failed to show an overall significant beneficial long-term effect of lower BP on decline in kidney function (Klahr and others, 1994;Wright, Jr. and others, 2002b) Both studies enrolled participants with non-diabetic CKD and randomized them to a mean arterial pressure (MAP) goal of <92 mm Hg (corresponding to <125/75 mm Hg) or a MAP goal of <107 mm Hg (corresponding to <140/90 mm Hg). The AASK trial compared two BP goals based on MAP (102-107 vs. <92 mm Hg) in 1094 African Americans with hypertensive kidney disease; the achieved difference of 128/78 vs. 141/85 did not reduce the progression of CKD (Wright, Jr. and others, 2002b). However, subgroup analyses of long-term (up to 10 years) post trial follow-up suggested the possibility of benefit in participants with baseline urinary protein excretion equivalent to >300 mg/day who were randomized to the lower goal (Appel and others, 2008). Among 585 non-diabetic participants with Stage 3/4 CKD in MDRD, 24% had PKD and only 53 were African American (Klahr and others, 1994). Mean baseline proteinuria was 2.2 g/d, and a beneficial effect of the lower BP goal on GFR was observed in the subgroup with urinary protein > 1 g/d (Peterson and others, 1995;Sarnak and others, 2005). In addition to the inherent problems associated with subgroup analysis, major caveats of these results from the MDRD Study were that the number of patients in the heavy proteinuric subgroups was small and the results were confounded by the use of angiotensin converting enzyme (ACE) inhibitors. Together, these studies fail to show convincing renoprotective effects for the lower BP goal; however their results have led to clinical recommendations that patients with high levels of proteinuria should have blood pressure goals below 140/90 mm Hg. They were not adequately powered to consider CVD outcomes. Nonetheless, they successfully demonstrated the feasibility of achieving significant separation in BP in large cohorts with advanced CKD. Given the rapid increase in the prevalence of CKD, the effects of aggressively lowering BP on the risks of CVD and CKD progression need to be clarified in a sample that appropriately mirrors the U.S. population with CKD (Sarnak and others, 2003).

1.2.2 SENIOR participants and SPRINT-MIND

Including a large subgroup of participants aged 75+ will provide data on whether intensive BP treatment will reduce CVD and renal events in the elderly. Both the Treatment of Hypertension in Patients over 80 Years of Age (HYVET) (Beckett and others, 2008) and the SHEP (SHEP, 1991) trials found that a SBP delta of 15 and 11 mm Hg, respectively, between treated and placebo groups resulted in >30% reduction in stroke, HF, and overall CVD events in the treated groups. Unlike HYVET and SHEP, which had SBP levels of about 150 and 143 mm Hg at the end of the trials, SPRINT will have a substantially lower SBP target of <120 mm Hg in the intensive treatment group, a goal which has never been tested in the elderly. No previous large scale trial has examined the impact of treating SBP in the elderly to <120 mm Hg versus <140 mm Hg.

Importantly, the elderly pose an additional question as to the safety of intensive SBP lowering in a population with known wider pulse pressures and a risk of excessively low DBP with intensive SBP treatment. In addition to concerns about hypotension, syncope, and falls, there may be a point of maximal benefit beyond which lowering BP could be detrimental in the elderly. This is a specific concern related to very low DBP, which could compromise coronary blood flow. The SPRINT-Senior cohort will allow us to more precisely assess the safety of the lower SBP goal.

The SPRINT Senior cohort also provides a critically important the main body of participants for SPRINT-MIND. Dementia is a leading cause of placement into nursing homes and assisted living facilities (guero-Torres and others, 2001;Guralnik and others, 1997;Magsi and Malloy, 2005;National Institute on Aging, 2000). Dementia affects 24 million individuals globally and 4.5 million persons in the US, a number that is expected to double by 2040 (Ferri and others, 2005;Plassman and others, 2007). Both dementia and a precursor, mild cognitive impairment (MCI), are highly prevalent among adults over age 70, with estimates running between 15-20% and 40-50% respectively in persons over age 80. In addition, there is evidence that MCI is also highly prevalent in persons above age 60 with CKD. Notably, approximately 15% of persons with MCI progress to dementia each year (Petersen, 2000), accruing substantial negative societal impact, and threatening the quality of life of its victims, their families and other caregivers. Proven strategies for prevention and delay of cognitive decline and dementia are lacking, and there is a clear need for clinical trials testing promising preventive interventions. Even a moderately effective strategy could have tremendous benefits, with a 5-year delay in onset of dementia estimated to decrease the number of cases of incident dementia by about 50% after several decades (Brookmeyer and others, 2002).

Cognitive impairment can have multiple etiologies and vascular risk factors are implicated in a large proportion of dementias including neurodegenerative dementias like Alzheimer's type (Qiu, Winblad, and Fratiglioni, 2005c). With this strong link to CVD risk plus several observational studies suggesting that the ideal SBP to lower CVD risk may be below 120 mm Hg (Chobanian and others, 2003) it is possible that targeting intensive blood pressure control intensive blood pressure control may have substantial implications for preserving brain function.

Substantial epidemiologic evidence identifies hypertension as a risk factor for dementia. Longitudinal observational studies have yielded mixed results, depending on the age at which blood pressure is measured, the impact and duration of treatment, duration of hypertension, and level of BP control (Birns and others, 2006;Qiu, Winblad, and Fratiglioni, 2005). Midlife hypertension appears to increase the risk of all-cause dementia in large prospective cohort studies (Freitag and others, 2006;Kivipelto and others, 2001b). However, lower SBP in older adults has been associated with subsequent development of dementia (Nilsson and others, 2007). Clinical trials of antihypertensive treatment have also provided conflicting experience regarding the impact of treatment of hypertension on the risk of cognitive impairment and dementia in older people (Guo and others, 1999;Hajjar and others, 2005; Veld and others, 2001). Four large randomized, placebo-controlled studies have investigated the effects of antihypertensive agents on the incidence of dementia. The Syst-Eur (Staessen and others, 1997) and the Perindopril Protection Against Recurrent Stroke Study (PROGRESS) studies (Tzourio and others, 2003) found that more aggressive antihypertensive treatment reduced the rate of small vessel ischemic disease (also the

primary outcome of SPRINT MIND MRI), a risk factor for dementia (Dufouil and others, 2009), as well as reducing dementia incidence by 50% compared to placebo. In contrast, the Study on Cognition and Prognosis in the Elderly (SCOPE) and SHEP trials (SHEP, 1991) found no significant difference in incidence of dementia between the active treatment and placebo groups, although differential missing data for the placebo vs. treatment groups may explain the SHEP findings (Di Bari and others, 2001). More recently, the HYVET-COG, a BP lowering trial in people age ≥80, was powered to detect a 33% reduction in adjudicated incident dementia (Peters 2008). The trial was stopped prior to its planned date of completion due to significant reductions in stroke and all-cause mortality in the intervention group. It yielded a 14% non-significant reduction in incident dementia. One reason for the non-significant result was a loss of power due to the unexpectedly early conclusion of follow-up, resulting in a relatively short, two-year period of follow-up. One possible explanation for the ambiguous relationships described between hypertension, hypertension treatment and preservation of cognitive function is that the cognitive measures included in most of these trials have not been sensitive enough to detect early, but clinically important, cognitive changes in a cohort with intact general cognitive function at baseline. Studies using more sensitive neuropsychological tests, such as the testing proposed for SPRINT-MIND, have shown the strongest relationships (Elias and others, 1993;Kivipelto and others, 2001a;Kivipelto and others, 2001c;Knopman and others, 2001).

Hypertension is the primary risk factor for small vessel ischemic disease and cortical white matter abnormalities (Basile and others, 2006;Kuller and others, 2010;Liao and others, 1996;Longstreth, Jr. and others, 1996). Chronic kidney disease is also associated with white matter abnormalities (Ikram and others, 2008), thus the SPRINT population is at high risk for significant white matter changes. Longitudinal studies document that hypertension-associated white matter abnormalities are an independent risk factor for cognitive decline and dementia (Verdelho and others, 2007;Vermeer and others, 2003), lower extremity functional abnormalities (Rosano and others, 2005), and clinical stroke (Debette and others, 2010). However, there is limited evidence that better control of BP slows the progression of white matter lesions in the brain (Dufouil and others, 2005). Recently reported results from the Women's Health Initiative Memory Study (WHIMS) indicate that white matter volume (detected by MRI) is associated with baseline BP, even after adjustment for treatment, other CVD risk factors, and age (Coker L.H. and others, 2008). Although the beneficial effects of treating hypertension on CVD, such as stroke have been shown (Collins and others, 1990), it is not known whether intensive lowering of SBP as proposed in SPRINT will provide reduction in the risk for developing white matter disease and brain volume loss.

SUMMARY

Higher than optimal BP is the leading cause of disability adjusted life-years lost on a global basis, and more intensive control of SBP than is currently recommended may contribute to reductions in stroke, heart failure, coronary heart disease, chronic kidney disease, and dementia. This potential benefit must be weighed against potential risks, including complications resulting from low coronary, cerebral, and renal perfusion pressure and the medications themselves. Definitive evidence from a well designed and conducted trial should form the foundation for pertinent recommendations and healthcare policies.

Chapter 2 – Overview of Trial Design

The SPRINT randomized controlled clinical trial will examine the effect of a high BP treatment strategy aimed at reducing SBP to a lower goal than is currently recommended. The primary objective is to determine whether randomization to this intensive strategy is more effective than a standard strategy in reducing the incidence of serious cardiovascular disease events. Other important study objectives are to assess the impact of more intensive SBP reduction on renal function, incidence of probable dementia, quality of life, cost-effectiveness, cognitive function and small vessel ischemic disease.

The study cohort will include approximately 9250 people aged ≥ 50 years with SBP ≥130 mm Hg. SPRINT will focus on three high risk groups: patients with clinical CVD other than stroke, patients with chronic kidney disease (estimated glomerular filtration rate (eGFR) 20 -59 mL/min/1.73 m^2), and patients who have a Framingham Risk Score (FRS) of ≥15%. Participants will be recruited over a 2-year period at approximately 80 to 100 clinics in 5 clinical center networks (CCNs) and will be followed for up to 6 years. Both the number of randomizations and the length of follow-up may differ from these targets depending on how observed values of parameters differ from estimates used to design the study. Approximately 4300 SPRINT participants will have CKD, and 3250 will be age 75 or older. Chapter 3 presents the eligibility criteria for the trial.

Participants will be stratified by clinic and randomly assigned to either the intensive or standard SBP lowering strategy. Chapter 4 and 5 provides a general description of the intervention.

The primary outcome will be a composite end-point consisting of the first occurrence of a myocardial infarction (MI, by electrocardiogram (ECG) or hospitalization), stroke, heart failure, non-MI acute coronary syndrome, or CVD death. Study outcomes are described in Chapters 6, 7 and 9.

The sample size for SPRINT is estimated to provide 90% power to detect a 20% relative decrease in the rate of the composite primary outcome in participants randomized to the more intensive SBP lowering strategy. Sample size estimation is described further in Chapter 10.

The major objectives of the SPRINT trial are as follows:

2.1 Primary Hypothesis

In people aged ≥ 50 years with SBP ≥130 mm Hg and either a history of CVD, eGFR between 20 and 59, or a Framingham Risk Score (FRS) indicating 10-year CVD risk of ≥15%, does a therapeutic strategy that targets a SBP of < 120 mm Hg reduce the rate of CVD events compared to a strategy that targets a SBP of < 140 mm Hg? This hypothesis will be tested using a composite outcome including

- cardiovascular death,
- myocardial infarction,
- stroke,
- heart failure, and
- non-MI acute coronary syndrome

ascertained over a follow-up period of up to 6 years. Interim monitoring for overall trial efficacy will be based on the accrued rate of this primary outcome. The anticipated event rate for this outcome is 2.2%/year.

2.2 Subgroup Hypotheses

SPRINT will examine intervention effects in a number of subgroups; these are presented in greater detail in Chapter 10. Two subgroups are of particular interest due their connection to possible biological mechanisms affecting the primary outcome:

1. participants with and without CKD (eGFR <60 ml/min/1.73m^2) at baseline,
2. participants < or ≥ 75 years at baseline.

Consistency of the effects for the intervention on the primary outcome will also be examined in subgroups defined by gender, race/ethnicity (black vs. non-black), presence of clinical CVD at baseline (i.e., primary and secondary prevention participants) and tertiles of baseline systolic BP.

Subgroup analyses for secondary outcomes are described in Chapter 10.

2.3 Secondary Hypotheses

SPRINT prespecifies two types of secondary hypotheses. The first type will address secondary outcomes in analyses designed to support and confirm the primary analysis. These will include components of the primary composite outcome, total mortality, and a composite of the primary composite with total mortality (CVD-free survival). The other type addresses two areas of non-cardiovascular clinical effects: renal and cognitive outcomes.

2.3.1 Objectives for renal outcomes and the CKD subgroup

1. For the CKD subgroup, we will determine whether the intensive intervention arm experiences a lower rate of a composite of renal outcomes composed of:
    - ESRD or
    - A 50% decline from baseline eGFR

2. For the non-CKD subgroup, we will determine whether the intensive intervention arm experiences a lower rate of progression to CKD, defined as
    - ESRD or
    - 30% decrease from baseline eGFR and an end value of <60 ml/min/1.73M^2

2.3.2 SPRINT MIND Hypotheses

1. All-cause Dementia. The incidence of all-cause dementia will be lower in SPRINT participants assigned to the intensive SBP treatment arm compared to their counterparts assigned to the standard SBP treatment arm.

2. Cognitive Decline. The combined rate of decline in all domains of cognition will be slower in the intensive SBP treatment arm compared to the standard SBP treatment

arm. This hypothesis will be tested in a randomly selected subset of 2800 participants enrolled in SPRINT.

3. MRI Brain Changes. The volume small vessel ischemic disease (SVID) will be lower in SPRINT participants assigned to the intensive SBP treatment arm compared to their counterparts assigned to the standard SBP treatment arm. A sub-hypothesis is that total brain volume will also be greater (thus less atrophy) in the intensively treated group. The MRI sub-study will be conducted in 640 participants chosen from the 2800 selected to receive regular extensive cognitive assessment.

Chapter 3 – Participant Selection

3.1 Eligibility Criteria

The objective of setting inclusion/exclusion criteria is to identify a trial population that will ensure adequate event rates for statistical power, provide maximum generalizability, and maximize safety. Inclusion/exclusion criteria were made as simple as possible to ensure standard implementation across all SPRINT study sites. Specifically, the SPRINT eligibility criteria were developed to facilitate the identification and inclusion of a trial population at high risk for the major trial endpoints, including CVD, CKD, cognitive decline, and dementia. Hence, the trial population is comprised of individuals in three major classes: those with existing CVD, existing CKD, or an elevated estimated risk for CVD disease based on age and other risk factors.

Implementation of these inclusion and exclusion criteria and related recruitment strategies will be accomplished to meet several goals with respect to composition of the study population. The overall goal for recruitment is 9,250 participants, although the final number of randomizations may be between 8,500 and 10,000. For the target of 9,250 participants, we will strive to include approximately 4300 (46%) with chronic kidney disease (eGFR 20 -59 ml/min/1.73m^2), expected to be divided approximately evenly below and above 45 ml/min/1.73m^2, and approximately 3250 (35%) who are at least 75 years old. In addition, we will strive to include 50% women, 40% minorities, and 40% with clinical or subclinical cardiovascular disease. Among these goals there is an implicit hierarchy based on study hypotheses and design considerations: first, attain the overall sample size, to preserve power for the main hypothesis of SPRINT; second, reach the required sample sizes for formal sub-group hypotheses among participants with CKD and among seniors; and third, ensure a sufficiently diverse study population so that results are broadly applicable to the affected U.S. population. We will monitor these goals on an ongoing basis and the Recruitment, Retention, and Adherence Subcommittee and the Steering Committee will evaluate recruitment strategies and implement corrective actions.

a) Inclusion Criteria

1. At least 50 years old

2. Systolic blood pressure
   SBP: 130 – 180 mm Hg on 0 or 1 medication
   SBP: 130 – 170 mm Hg on up to 2 medications
   SBP: 130 – 160 mm Hg on up to 3 medications
   SBP: 130 – 150 mm Hg on up to 4 medications

3. There are no diastolic blood pressure (DBP) inclusion criteria, since risk is more related to SBP than DBP in the age and risk population anticipated for SPRINT. If a screenee is otherwise eligible for SPRINT but presents with a treated BP and/or number of medications that fall outside the SPRINT inclusion criteria, BP-lowering medications may be adjusted prior to the randomization visit to determine whether, with such adjustments, the screenee will meet eligibility criteria for SPRINT. A screenee who presents on no BP medications should have documentation of SBP

≥130 mm Hg on 2 visits within 3 months prior to the randomization visit in order to be eligible for the trial.

4. Risk (one or more of the following):
   a) Presence of clinical[*] or subclinical[**] cardiovascular disease other than stroke
   b) CKD, defined as eGFR   20 – 59 ml/min/1.73m^2 based on the 4-variable Modification of Diet in Renal Disease (MDRD) equation and latest lab value, within the past 6 months. (If the serum creatinine is unstable within the last 6 months, enrollment into SPRINT could be delayed until the serum creatinine has been stabilized and the eGFR is still within the allowed range.)
   c) Framingham Risk Score for 10-year CVD risk ≥ 15% based on laboratory work done within the past 12 months for lipids
   d) Age ≥ 75 years.

5. Clinical CVD (other than stroke)
   a) Previous myocardial infarction (MI), percutaneous coronary intervention (PCI), coronary artery bypass grafting (CABG), carotid endarterectomy (CE), carotid stenting
   b) Peripheral artery disease (PAD) with revascularization
   c) Acute coronary syndrome with or without resting ECG change, ECG changes on a graded exercise test (GXT), or positive cardiac imaging study
   d) At least a 50% diameter stenosis of a coronary, carotid, or lower extremity artery
   e) Abdominal aortic aneurysm (AAA) ≥5 cm with or without repair

6. Subclinical CVD
   a) Coronary artery calcium score ≥ 400 Agatston units within the past 2 years.
   b) Ankle brachial index (ABI) ≤0.90 within the past 2 years.
   c) Left ventricular hypertrophy (LVH) by ECG (based on computer reading), echocardiogram report, or other cardiac imaging procedure report within the past 2 years.

b) Exclusion Criteria

1. An indication for a specific BP lowering medication (e.g., beta-blocker following acute myocardial infarction) that the person is not taking and the person has not been documented to be intolerant of the medication class. (If a screenee has a non-hypertension indication for a BP-lowering medication (e.g., beta-blocker post-MI, renin angiotensin system (RAS) blocker for CVD prevention, or alpha blocker for benign prostatic hypertrophy (BPH)), the screenee should be on the appropriate dose of such medication before assessing whether he/she meets the SPRINT inclusion criteria. If the investigator believes that a potential participant has such an indication but is not receiving appropriate treatment, he/she should encourage the potential participant's primary care provider to consider placing the patient on the appropriate therapy prior to proceeding with the screening process.)
2. Known secondary cause of hypertension that causes concern regarding safety of the protocol.
3. One minute standing SBP < 110 mm Hg. Not applicable if unable to stand due to wheelchair use.

4. Proteinuria in the following ranges (based on a measurement within the past 6 months)
    (a) 24 hour urinary protein excretion ≥1 g/day, or
    (b) If measurement (a) is not available, then 24 hour urinary albumin excretion ≥ 600 mg/day, or
    (c) If measurements (a) or (b) are not available, then spot urine protein/creatinine ratio ≥ 1 g/g creatinine, or
    (d) If measurements (a), (b), or (c) are not available, then spot urine albumin/creatinine ratio ≥ 600 mg/g creatinine, or
    (e) If measurements (a), (b), (c), or (d) are not available, then urine dipstick ≥ 2+ protein
5. Arm circumference too large or small to allow accurate blood pressure measurement with available devices
6. Diabetes mellitus. Participants taking medications for diabetes at any time in the last 12 months are excluded. Participants are also excluded if there is documentation of: FPG at or above 126 mg/dL, A1C ≥6.5 percent, a two-hour value in an OGTT (2-h PG) at or above 200 mg/dL or a random plasma glucose concentration ≥200 mg/dL. The diagnosis of diabetes must be confirmed on a subsequent day by repeat measurement, repeating the same test for confirmation. However, if two different tests (eg, FPG and A1C) are available and are concordant for the diagnosis of diabetes, additional testing is not needed. If two different tests are discordant, the test that is diagnostic of diabetes should be repeated to confirm the diagnosis.
7. History of stroke (not CE or stenting)
8. Diagnosis of polycystic kidney disease
9. Glomerulonephritis treated with or likely to be treated with immunosuppressive therapy
10. eGFR < 20 ml/min /1.73m^2 or end-stage renal disease (ESRD)
11. Cardiovascular event or procedure (as defined above as clinical CVD for study entry) or hospitalization for unstable angina within last 3 months
12. Symptomatic heart failure within the past 6 months or left ventricular ejection fraction (by any method) < 35%
13. A medical condition likely to limit survival to less than 3 years, or a cancer diagnosed and treated within the past two years that, in the judgment of clinical study staff, would compromise a participant's ability to comply with the protocol and complete the trial. Exceptions to the exclusion for diagnosed cancer would include, for example, non-melanoma skin cancer, early-stage prostate cancer, localized breast cancer.
14. Any factors judged by the clinic team to be likely to limit adherence to interventions. For example,
    (a) Active alcohol or substance abuse within the last 12 months
    (b) Plans to move outside the clinic catchment area in the next 2 years without the ability to transfer to another SPRINT site, or plans to be out of the study area for more than 3 months in the year following enrollment.
    (c) Significant history of poor compliance with medications or attendance at clinic visits
    (d) Significant concerns about participation in the study from spouse, significant other, or family members
    (e) Lack of support from primary health care provider

(f) Residence too far from the study clinic site such that transportation is a barrier including persons who require transportation assistance provided by the SPRINT clinic funds for screening or randomization visits
(g) Residence in a nursing home. Persons residing in an assisted living or retirement community are eligible if they meet the other criteria.
(h) Clinical diagnosis of dementia, treatment with medications for dementia, or in the judgment of the clinician cognitively unable to follow the protocol
(i) Other medical, psychiatric, or behavioral factors that in the judgment of the Principal Investigator may interfere with study participation or the ability to follow the intervention protocol

15. Failure to obtain informed consent from participant
16. Currently participating in another clinical trial (intervention study). Note: Patient must wait until the completion of his/her activities or the completion of the other trial before being screened for SPRINT.
17. Living in the same household as an already randomized SPRINT participant
18. Any organ transplant
19. Unintentional weight loss > 10% in last 6 months
20. Pregnancy, currently trying to become pregnant, or of child-bearing potential and not using birth control

c) Additional Criteria

I. SENIOR

Whereas there are no eligibility criteria specific to the SENIOR subgroup other than age, the general eligibility criteria were influenced by consideration of factors of importance to the inclusion of older participants in SPRINT, including cognitive status, orthostasis, transportation, and site of residence (e.g., nursing home). The goal is to assemble a representative population of older patients for whom intensive BP lowering is reasonable to consider from a medical perspective. This goal is motivated by the perspective that there may be some older persons with advanced frailty and/or multiple comorbid conditions whose health is so poor that it would not be reasonable to attempt to treat SBP as intensively as needed to control SBP to less than 120 mm Hg.

II. Participants with CKD

For the purposes of SPRINT, qualifying CKD is defined by eGFR, determined during the 6 months prior to randomization, between 20 and 59 ml/min/1.73m^2, inclusive, based on the 4-variable MDRD equation. Patients with significant proteinuria, defined as a 24-hour urine protein excretion exceeding 1 gram, or rough equivalents thereof (see Exclusion Criterion 4 above), will be excluded from SPRINT based on evidence from previous trials suggesting that intensive BP lowering therapy may be beneficial with respect to slowing the progression of CKD. The vast majority of participants with CKD so defined will likely be at high risk for CVD. An estimated 82.3% of those who qualify with eGFR between 45 and 59 ml/min/1.73m^2 will have a Framingham Risk Score for CVD exceeding 15% over 10 years, and an estimated 71.2% have a Framingham Risk Score for CVD exceeding 20% over 10 years; hence, these participants will contribute substantially to the overall event rate and provide the basis for informative subgroup analyses.

III. MIND

Dementia Screening - All individuals will receive dementia screening at baseline and every 2 years following baseline. Individuals who have been previously diagnosed with dementia by their physicians are excluded from SPRINT and SPRINT MIND.

Comprehensive Cognitive Assessment substudy – A subset of 2800 participants enrolled in SPRINT will also be assigned to undergo more extensive cognitive assessment to evaluate the impact of the intervention on decline in overall and domain-specific cognitive function that does not meet criteria for dementia. With limited exceptions, all clinics will enroll participants into this 2800 subset, and this subgroup is expected to be representative of all randomized participants, including the important CKD and SENIOR participants.

IV. MIND MRI

Individuals who enroll in the Comprehensive Cognitive Assessment substudy at a clinic within sufficient proximity to a SPRINT MIND MRI center, generally defined as within a 2 to 3 hour driving radius, are eligible to enroll in the MIND MRI Study. The MIND MRI Study will have a recruitment goal of approximately 640 participants. Standard safety-related exclusions pertaining to the ability to have a magnetic resonance imaging procedure performed will be applied.

Recruitment and risk implications of inclusion and exclusion criteria

As shown in Table 3.1, according to analyses of the National Health and Nutrition Examination Survey (NHANES) data for 1999-2004, approximately 6% of the US population meets the basic eligibility criteria related to age and SBP, and are free of diabetes and previous stroke. Among that group, approximately 70% meet the risk criteria described above. The vast majority of these individuals have an estimated 10-year risk of CVD exceeding 20% and the population average 10-year risk for CVD is approximately 28%. (Note that the use of the FRS in this manner likely underestimates the risk of those individuals with existing CHD and stage 3 CKD.) This analysis provides evidence that the recruitment pool will be large enough to enable us to recruit successfully and to generalize our ultimate results to a reasonably large proportion of the US population.

Table 3.1. Distribution of 10-year risk of CVD in NHANES participants who met basic SPRINT eligibility criteria

Criteria	% of US Population meeting basic eligibility criteria (age, SBP, no DM or stroke)	% of those meeting basic eligibility requirements who meet risk criteria	10-year CVD Risk Distribution (%)				Mean 10-yr CVD risk (%)
			5-10%	10-15%	15-20%	20+%	
CHD or Stage 3 CKD or FR≥15%	6.7	70.3	1.3	3.2	24.3	71.1	28.6

In additional analyses of the NHANES potentially eligible pool, 16.3% had stage 3 CKD (3.6% had eGFR < 45ml/min/1.73m^2), 15.6% had a history of CVD, 34.6% were 75 years old or older, 8.1% were African Americans, and 49.8% were women. Stage 3a CKD, defined as eGFR 45-59 ml/min/1.73m^2, but a urine albumin-to-creatinine (ACR) ≤

10 mg/g, comprised 6.1% of the eligible pool.. These analyses, shown in Table 3.2, provide evidence to support our recruitment targets for participants with CKD, in the SENIOR population, minorities and women.

Table 3.2. Characteristics of SPRINT eligible sample based on NHANES data. Eligibility requirements include age≥50, SBP≥130, eGFR> 20, ACR<600 mg/g and no history of stroke or diabetes.

Characteristic	Proportion (%)
% Prior CVD	15.6
% CKD	16.3
% Stage 3b CKD	3.6
% Stage 3a + ACR > 10 mg/g	6.6
% Stage 3a + ACR ≤ 10 mg/g	6.1
% Senior (age≥75)	34.6
% Female	49.8
% Black	8.1
% Hispanic	7.4
% SBP 130-139 on no BP lowering medications	15.2
% with FRS < 15% per 10 yrs	4.5

3.2 Recruitment: Informed Consent, Screening, Baseline

Recruitment

The SPRINT recruitment goals are described above. Specific community resources will be used to target women and minority/under-served populations to ensure adequate representation of these groups in SPRINT. Recruitment strategies that have worked well in other trials related to hypertension and CKD will be used. Centralized training for CCN and Clinical Site staffs regarding recruitment issues will be provided before recruitment begins.

The goal of participant recruitment is to create a trial population that will ensure adequate event rates for statistical power while maximizing participant safety and generalizability to the population for which the intervention is intended. A multifaceted approach to screening and enrollment is essential to achieve the recruitment goal. For this multicenter trial, recruitment strategies targeting both existing populations within the clinical practice of the research sites as well as individuals from outside these practice settings will be used to identify potentially eligible participants.

The Recruitment, Retention and Adherence Subcommittee will play a significant role in monitoring the progress of study-wide recruitment and provide a forum for advising the CCNs and clinical sites on problem identification, goal setting, strategy deployment and evaluation in their efforts to achieve site and study-wide recruitment goals. This may include guidance for enhancing the recruitment of ethnic groups, women and the elderly. The Subcommittee will also contribute to the development of the recruitment tools including culture-, gender- and age-specific materials to promote enrollment among these important subgroups.

3.2.1 Regulatory and Ethical Considerations, including the Informed Consent Process

The study will be conducted in accordance with Good Clinical Practice (GCP), all applicable subject privacy requirements, and the guiding principles of Helsinki, including but not limited to:

1. Local Institute Review Board (IRB)/Central IRB review and approval of study protocol and any subsequent amendments.
2. Subject informed consent for main trial, SPRINT MIND, genetic testing, and post trial contact, and any ancillary studies. The study consent will contain the six essential elements from GCP guidelines that include:
    - Research statement, reasonably foreseeable risks or discomforts, reasonably expected benefits to subjects or others, appropriate alternatives, extent of confidentiality, compensation or treatment for injury.
    - Additional elements where appropriate such as unforeseeable risks to subjects, embryos, or fetuses, investigator-initiated termination of participation, additional costs, significant new findings, authorization for release of protected health Information for research purposes.
3. Investigator reporting requirements.

Written informed consent and Health Insurance Portability and Accountability Act (HIPAA) authorization must be obtained from each person prior to enrollment into SPRINT. In collaboration with the CCNs, the SPRINT Coordinating Center will provide full details and template documents for the above procedures in the Manual of Procedures and provide training to the investigators and clinical staff on regulatory and ethical considerations. All study personnel will be responsible for completing and remaining current with all applicable human subjects' protection, good clinical practice and data security and privacy training requirements

3.2.2 Existing Populations in the Clinical Site Practices
Methods for identifying potentially eligible participants within the clinical practice of the research settings may include: a targeted review of medical records or databases for those meeting the trial's inclusion criteria, referrals from providers/employees within the practice and/or from practice participants themselves. Additional approaches may also include written materials such as direct mailing and/or advertisement on such items as appointment reminders.

3.3. Screening Visits/ Baseline Visits

Screening Activity Considerations
Each SPRINT clinical center should consult their local IRB regarding approval requirements to access internal medical record searches for potential SPRINT patients. Depending upon the institution, prior approvals for data transfer agreements may be needed to obtain de-identified patient information. Pursuant to such agreements investigators may be required to sign a privacy agreement to protect the patient's protected health information (PHI) as well as comply with other policies and procedures as defined by the institution's designated privacy, security and compliance services.

SPRINT clinical centers will work with the respective CCNs to complete Health Insurance Portability and Accountability Act (HIPAA) Privacy rule documents, preparatory to research waivers and training prior to patient medical record searches.

Once local regulatory requirements have been approved, investigator plans to identify potential study patients may be implemented. Large scale data base searches, stratified by key specified inclusion criteria may also yield a global assessment of the potentially eligible study population. Other study parameters (e.g. age, race, gender CKD status, etc.) can be added to further specify the eligible population.

Prior to conducting prescreening and screening activities, it may also be necessary to request additional approvals beyond the IRB (e.g. physician approval or consultation for a screening referral to the SPRINT clinic). Participant informed consent must also be obtained prior to performing any procedures related to the trial.

Screening Visits/Baseline Visit
The following are key elements of the screening and baseline visits and are outlined in the study assessments and procedures below:

Screening Visit(s)
1. Verify participant's interest in study.
2. Obtain in person study consent and HIPAA authorization for main trial, and if applicable, SPRINT MIND, genetic testing and any ancillary studies
3. Continue collection of screening information, including such items as contact information, additional eligibility information including BP measurement, concomitant medications, and medical history.

Baseline visit (Randomization Visit)
1. Confirmation that all inclusion/exclusion criteria satisfied
2. Verification of participant consent and HIPAA authorization.
3. Verification of participant contact information
4. Obtain a Release of Information, as permitted by local policy, to collect event and serious adverse event (SAE) documentation
5. Completion of the study randomization procedure and baseline data collection, including obtaining BP, ECG, and blood and urine samples for analysis and storage at the central lab

Data obtained from the screening, and randomization visits must be supported in the patient's source documentation. Visit data will be entered into the SPRINT database within a specified time frame determined by the SPRINT Coordinating Center.

Chapter 4 – Intervention

Blood Pressure Goals

Participants eligible for the trial will be randomized to one of two goals: SBP <120 mm Hg for the more intensive goal (Intensive Group) and SBP <140 mm Hg for the less intensive goal (Standard Group). Figures 4.1 and 4.2 describe the treatment algorithms for the two treatment groups. Although there are no diastolic blood pressure (DBP) inclusion criteria, participants in both groups with DBP ≥90 mm Hg will be treated to a DBP goal of <90 mm Hg if needed after meeting the SBP goal, because of the many trials documenting the CVD benefits in treating to a DBP goal <90 mm Hg.

Antihypertensive Classes (Agents)

Use of once-daily preparations of antihypertensive agents will be encouraged unless alternative dosing frequency (e.g., BID) is indicated/necessary. One or more medications from the following classes of agents will be provided by the study and intended for use in managing participants in both randomization groups to achieve study goals:

- Angiotension converting enzyme (ACE)-inhibitors
- Angiotension receptor blockers (ARBs)
- Direct vasodilators
- Thiazide-type diuretics
- Loop diuretics
- Potassium-sparing diuretics
- Beta-blockers
- Sustained-release calcium channel blockers (CCBs)
- Alpha1-receptor blockers
- Sympatholytics

Combination products will be available, depending on cost, utility, or donations from pharmaceutical companies

Selection of Antihypertensive Medications

The SPRINT trial is testing a treatment strategy question regarding different SBP goals and not testing specific medications. The SPRINT BP treatment protocol is flexible in terms of the choice and doses of antihypertensive medications, but there should be preferences among the drug classes, based on CVD outcome trials results and current guidelines. NHLBI is updating various guidelines. The update of hypertension recommendations, JNC-8, should be available early in the recruitment phase of SPRINT. These updates, along with any new scientific developments, will be considered during and following SPRINT protocol development and throughout the trial.

The investigator may select among the available SPRINT antihypertensive medications for initiation of therapy. Other drugs not supplied by the trial may also be used as the investigator determines appropriate. However, all antihypertensive regimens should include one or more drug classes with strong CVD outcome data from large randomized controlled hypertension trials, i.e., a thiazide-type diuretic, calcium channel blocker, ACE inhibitor or ARB. Current evidence, the most recent JNC guidelines and over 40 years

of clinical trial experience in hypertension support the inclusion of a thiazide-type diuretic as one of the agents for patients without compelling reasons for another medication, or contraindication or intolerance to a thiazide-type diuretic. (ALLHAT, 2002;Beckett and others, 2008;Chobanian and others, 2003;Psaty and others, 1997;SHEP, 1991) Other classes associated with substantial reductions in CVD outcomes in hypertension trials, e.g. ACE inhibitors, ARBs, and calcium channel blockers, combine effectively with thiazides for lowering BP (Julius and others, 2004). ACE inhibitors and ARBs also combine well with CCBs; if three drugs are needed, a thiazide-type diuretic, a RAS blocker (ACE inhibitor or ARB, but usually not both), and CCB make a very effective and usually well-tolerated regimen (Calhoun and others, 2009). The preference for the order in which these agents are selected is left to the investigator as long as the SBP goals are achieved. A loop diuretic may be needed in addition to or in place of a thiazide-type diuretic for participants with advanced CKD.

Beta-adrenergic blockers, which were recommended in JNC-7 among the 4 preferred classes after diuretics, are now considered to be less effective in preventing CVD events as primary treatment of hypertension compared with thiazide-type diuretics, CCBs, and RAS blockers (Lindholm, Carlberg, and Samuelsson, 2005) However, there are patients for whom beta-blockers should be part of the initial therapy, namely those with coronary artery disease, including chronic stable angina or previous MI (Rosendorff and others, 2007).

Finally, although renoprotective benefits have been demonstrated in CKD patients with proteinuria, ACE inhibitors (and likely other RAS blockers) are less effective than other classes in lowering BP and in preventing CVD events in African American and elderly hypertensive patients unless combined with a diuretic or CCB (Julius and others, 2004;Mancia and others, 2007;National Collaborating Centre for Chronic Conditions, 2006;Wright and others, 2005;Wright and others, 2008).

Since more than three drugs will be necessary in many participants to reach the intensive SBP goal, other classes will also be available in SPRINT. These include the potassium-sparing diuretics, spironolactone and/or amiloride, which are very effective as add-on agents for BP-lowering in “resistant hypertension” (Calhoun and others, 2008). However, they should be used with careful monitoring in participants with CKD or any tendency to hyperkalemia. Alpha-blockers have been used effectively as add-on therapy in the AASK, ACCORD and Anglo-Scandinavian Cardiac Outcomes (ASCOT) trials. However, alpha-blockers should be used only in combination with one or more other agents proven to reduce CVD events in hypertensive patients (ALLHAT, 2003). Sympatholytics, direct vasodilators, and/or loop diuretics may also be added for BP control in combination with agents proven to reduce CVD events.

Among thiazide-type diuretics, the most consistent and robust CVD outcome data have been seen with chlorthalidone (ALLHAT, 2002;SHEP, 1991). Chlorthalidone 12.5-25 mg/d has been shown to be more effective in lowering BP over 24 hours than hydrochlorothiazide 25-50 mg/d (Ernst and others, 2006). Among CCBs, amlodipine has been used in far more hypertension CVD outcome trials than any other agent and has more robust CVD outcome data. Amlodipine should be considered first when a CCB is to be used. In the presence of significant proteinuria, amlodipine should probably be used in conjunction with a RAS blocker. If a non-dihydropyridine CCB (e.g., diltiazem) is to be used, it should not be combined with a beta-blocker.

The ACCORD experience (The ACCORD Study Group, 2010) has shown that a treatment strategy that includes a variety of classes, can produce a 14 mm Hg delta in SBP between the two randomized groups. The average number of antihypertensive drugs used to produce this difference was 3.4 and 2.1 in the Intensive and Standard Groups, respectively. It is anticipated that the study participants in the CKD subgroup of SPRINT will require a greater number of antihypertensive drugs to reach the lower BP goal (Cushman and others, 2008)

Visit Frequency

For both randomized groups, routine visit frequency will be monthly for the first three months after randomization, then every three months for the duration of the trial. "Monthly visits will continue in the Intensive Group until SBP <120 mm Hg (or no more titration planned) and in the Standard group whenever SBP ≥ 160 mm Hg." Additional visits will be scheduled as needed for management of adverse effects or for monitoring significant medication changes or other clinical issues.

Intensive BP Goal Group (Figure 4.1)

The SBP goal for the Intensive Group, <120 mm Hg, should be achievable in the majority of participants within 8-12 months of follow-up based on the ACCORD experience (The ACCORD Study Group, 2010). For most participants in the Intensive Group, a two- or three-drug regimen of a diuretic and either an ACE inhibitor or ARB and/or a CCB should be initiated at randomization. If a diuretic is contraindicated or not tolerated, an ACE inhibitor or ARB plus a CCB should be initiated. A beta-blocker should be included in the initial regimen, usually in combination with a diuretic, if there is a compelling indication for a beta-blocker. Drug doses should be increased and/or additional antihypertensive medications should be added at each visit in the Intensive Group, usually at monthly intervals, until the participant's goal of <120 mm Hg has been reached or the investigator decides no further antihypertensive medications may be added.

SPRINT provides a unique opportunity to determine both the efficacy and safety of intensive BP control in elderly populations. However, based on limited data, there is a concern that this population may be less tolerant of aggressive BP lowering. Therefore, in participants ≥75 years of age randomized to the intensive BP goal who are on 0-1 antihypertensive medications and have baseline SBP <140 mm Hg, antihypertensive therapy may be initiated with a single agent at the discretion of the investigator with a return visit scheduled in one month. If the participant is asymptomatic at the first post-randomization visit and SBP ≥130 mm Hg, a second agent will be added and titration continued as indicated in above.

Milepost Visits

"Clinical inertia" in hypertension management, where clinicians fail to intensify therapy despite patients not being at goal BP, has been observed in both clinical practice (Berlowitz and others, 1998) and clinical trial settings (Cushman and others, 2002). For this reason, "Milepost Visits" were used in the intensive BP group in the ACCORD trial to assist in reaching goal SBP (Cushman and others, 2007). For SPRINT participants in the Intensive Group, Milepost Visits will be every 6 months throughout follow-up,

beginning at the 6-month visit. If the SBP is not <120 mm Hg at a Milepost Visit, then an antihypertensive drug from a class different from what is being taken should be added, unless there are compelling reasons to wait. A "Milepost Exemption Form" will be completed whenever a new drug is not added at a Milepost Visit in which the participant's BP is not <120 mm Hg to document the reason for not adding a drug and to outline a plan for making progress toward goal in that participant. Milepost Visit procedures do not apply to the Standard Group. Once the Intensive Group participant has been prescribed 5 drugs at maximally tolerated doses, if the BP remains above goal at subsequent Milepost Visits, it will be permitted to substitute a different class into the regimen instead of adding another drug or increasing the dose of a drug. However, additional (more than 5) drugs may be needed to achieve goal SBP in some participants. Medication adherence will be assessed routinely in SPRINT and should be evaluated especially carefully for participants not at goal on 4 or more medications. Strategies to enhance adherence are described in brief in Chapter 5 and in detail in the Manual of Procedures and Adherence Binder.

Standard BP Goal Group (Figure 4.2)

The SBP goal for the Standard Group, <140 mm Hg, should be achievable in the majority of participants within 3-6 months, based on the ACCORD experience (The ACCORD Study Group, 2010). The standard BP protocol is designed to achieve a SBP of 135-139 mm Hg in as many participants as possible. Participants in this group may or may not be on treatment with one or more antihypertensive medications. If antihypertensive medication(s) is indicated per protocol, consideration should be given to including a thiazide-type diuretic as initial therapy or as part of the regimen, unless there is a compelling indication for another drug class or intolerance to a thiazide.

At the randomization visit, Standard Group participants on previous antihypertensive drug therapy should be converted to SPRINT medications or no medications, depending on what the investigator believes is most likely to achieve a SBP level between 135-139 mm Hg. Because we expect a decrease in average SBP within the Standard Group following randomization due to improved adherence, lifestyle counseling, and intra-individual variation, sometimes described as "regression to the mean", treatment should not be intensified at the randomization visit for Standard Group participants unless SBP ≥160 mm Hg or there is a compelling reason to add medication, e.g., management of fluid balance in participants with CKD. Following the randomization visit, medication dose titration or addition of another drug is indicated if SBP is ≥160 mm Hg at a single visit or is ≥140 mm Hg at two successive visits.

Because it is not known if lowering SBP to the more intensive SPRINT goal of <120 mm Hg, compared with the standard goal of <140 mm Hg, is beneficial, neutral, or harmful in patients such as those entered into the SPRINT trial, careful step-down (a reduction of the dose or number of antihypertensive drugs) is allowed for participants in the Standard Group. Down-titration was not permitted in the HOT Trial if DBP was well below the goal for a participant (Hansson and others, 1998) – this likely contributed to the small differences in achieved BP between the three randomized groups and limited the study's ability to detect differences in outcomes. Therefore, down-titration was included in the ACCORD and AASK standard BP protocols and was successful in generating the planned differences in BP between treatment arms. Down titration should be carried out if the SBP is <130 mm Hg at a single visit or <135 mm Hg at two consecutive visits (Figure 4.2).

Diastolic Blood Pressure Treatment

Once the SBP goal has been achieved in any participant, the antihypertensive regimen should be intensified if DBP remains ≥100 mm Hg at a single visit or ≥90 mm Hg at two successive visits to achieve DBP <90 mm Hg. The visit intervals and decisions for titration (other than the BP levels) will be similar to those used for the SBP goal. Since beta-blockers reduce DBP more than SBP relative to other antihypertensive medications, a beta-blocker could be considered for such participants (Cushman and others, 2001).

Use of Home BP Devices

Home BP devices will not be provided to all participants by the trial. Since virtually all BP outcome trials have used office BP determinations and home readings are subject to more bias and error, in SPRINT titration of medications to goal should be based on office readings rather than home BP determinations.

Assessment of Orthostatic Hypotension (OH), Measurement of Standing Blood Pressure

Standing BP will be measured at screening, baseline, 1 month, 6 months, 12 months, and annually thereafter using the same BP device that is used to measure seated BP. After seated determinations, participants will be asked to stand. Beginning when their feet touch the floor, BP will be taken one minute later in the same arm used for the seated measurements, using the BP device. Participants will be asked after the standing determination if they had any symptoms of orthostatic hypotension during the standing BP measurement. The Coordinating Center will calculate BP change using the standing measurements minus the mean of the seated measurements.

Participants with standing SBP <110 mm Hg will not be eligible for randomization (may be rescreened if corrected). However, the detection of asymptomatic orthostatic hypotension, i.e., orthostatic hypotension unaccompanied by orthostatic symptoms of dizziness, presyncope or syncope, will not influence the antihypertensive drug treatment algorithm. Symptomatic orthostatic hypotension will be managed as described in "Management of Symptomatic Orthostatic Hypotension" (see Manual of Procedures).

4.1 Lifestyle Recommendations and Background Therapy

The purpose of including lifestyle recommendations and background therapy in SPRINT is twofold. First, it fosters high quality general medical care in all SPRINT participants in accordance with current practice guidelines. Second, it is intended that background therapies will be utilized equally across both study arms in order to minimize the differences in the effects of non-study strategies on the SBP or CV outcomes between arms. The background therapy recommendations will be provided to the participants and their physicians. Background therapy is considered part of usual recommended care for patients at risk of CVD and, as such, is not covered by research study costs. The delivery of these background therapies will be left up to the participants' own clinicians.

The Lifestyle and Background Therapy Working Group will coordinate the provision of the most current and relevant participant educational materials to be made available for study-wide use. These will include the topics of medical nutrition therapy, weight management, physical activity, smoking cessation, and anti-thrombotic therapy, and will complement educational materials related to the BP interventions that are part of the trial. Unlike most educational materials for BP, the SPRINT materials will not include specific goals for BP as these will depend on the participants' randomized treatment assignment. Specific recommendations will include: a) weight loss in those who are overweight or obese; b) adoption of a diet rich in fruits, vegetables and low-fat dairy products (the DASH diet) with appropriate modifications for participants with CKD; c) reduction in sodium intake to recommended levels; d) reduction of alcohol consumption to recommended levels; and e) participation in regular aerobic exercise. SPRINT participants will be encouraged to stop smoking (if a current smoker) and to follow current guidelines for testing for and treatment of dyslipidemia and the use of antithrombotic therapy.

Figure 4.1 Treatment Algorithm for Intensive Group (Goal SBP < 120 mm Hg)

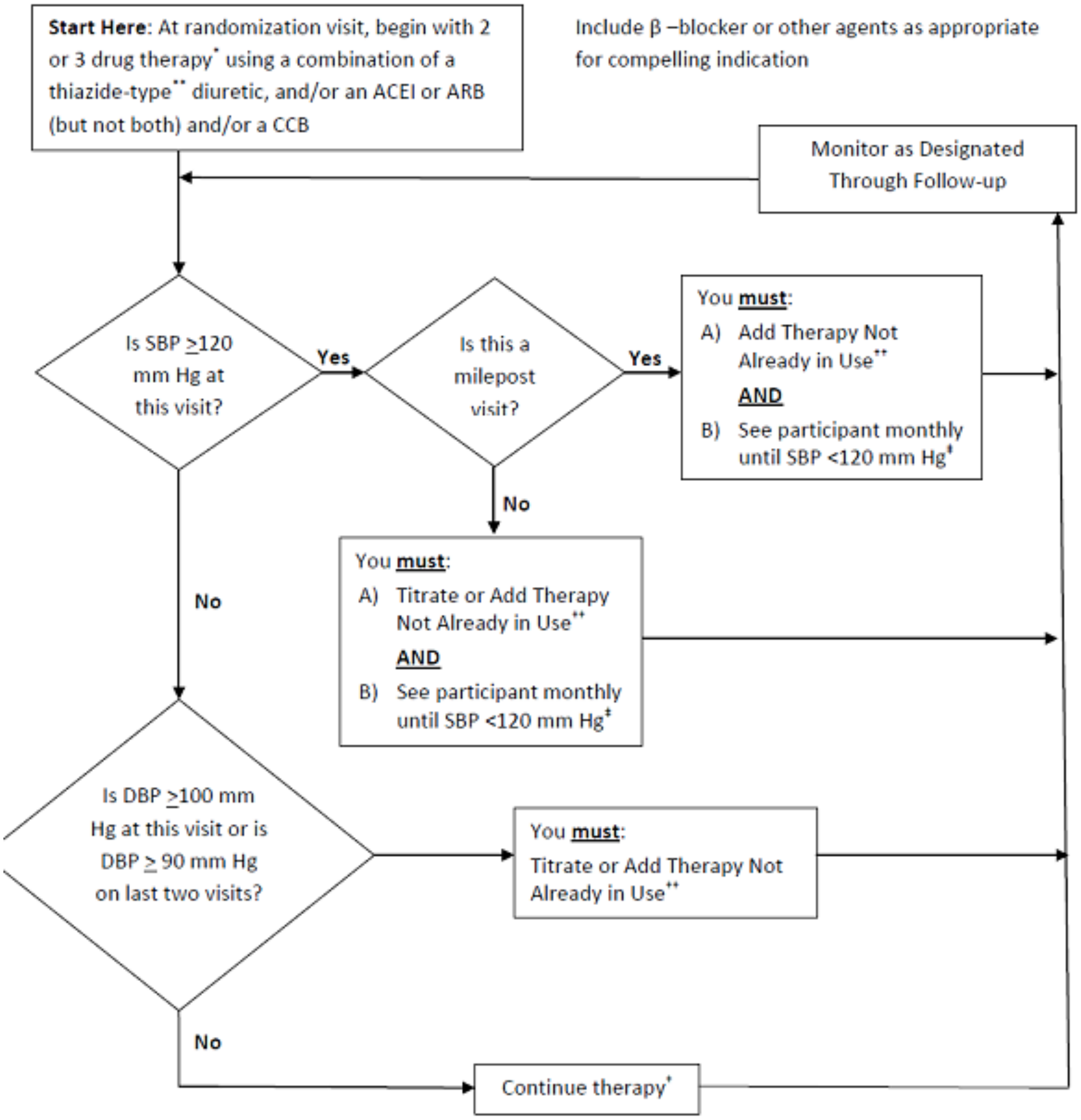

* May begin with a single agent for participants 75 years old or older with SBP < 140 on 0-1 meds at study entry. A second medication should be added at the 1 Month visit if participant is asymptomatic and SBP ≥ 130.

** May use loop diuretic for participants with advanced CKD

† Unless side effects warrant change in therapy

†† Consider consulting with the Clinical Center Network before adding a fifth anti-hypertensive medication

‡ Or until clinical decision made that therapy should not be increased further

Figure 4.2 Treatment Algorithm for Standard Group (Goal SBP < 140 mm Hg)

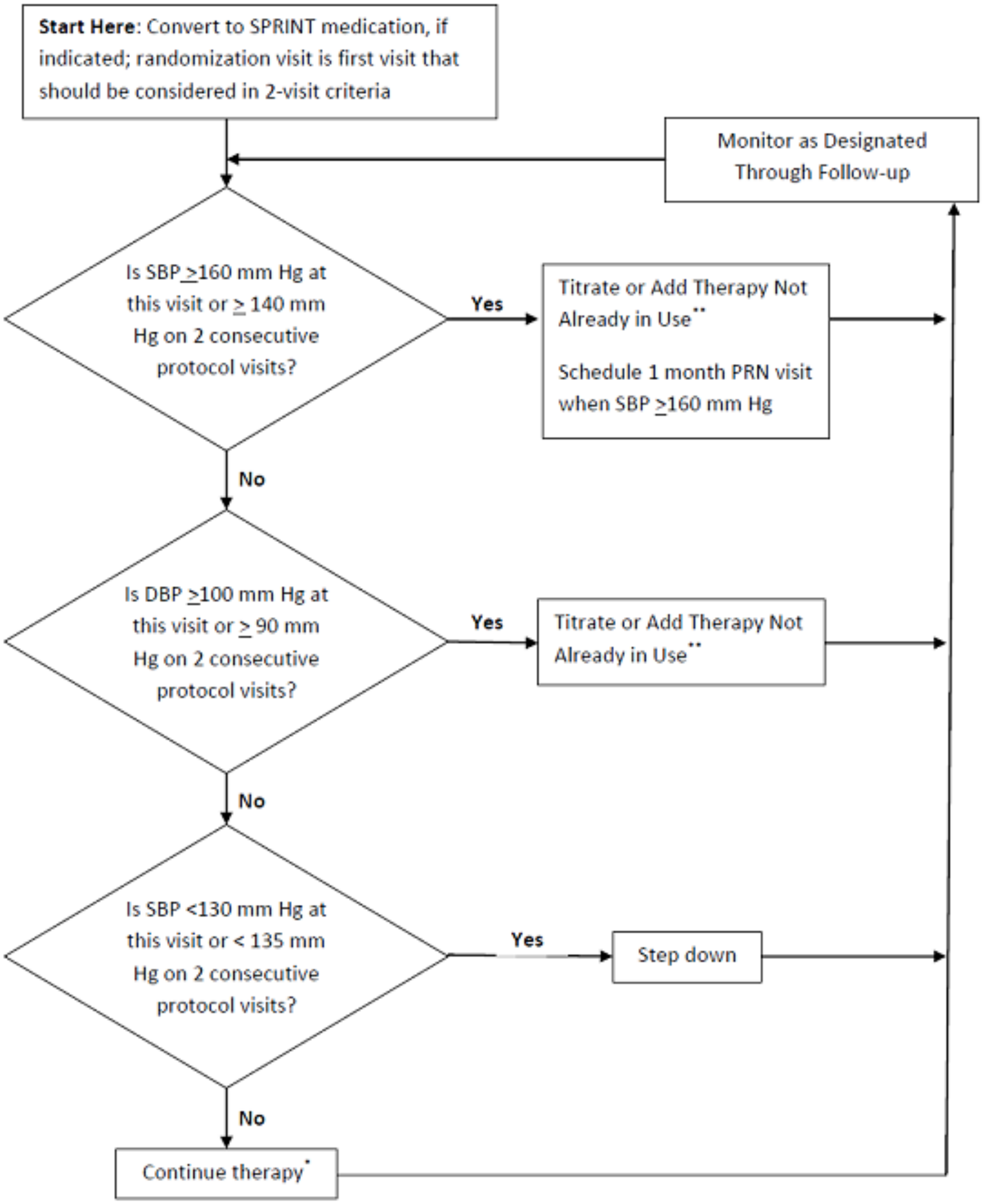

Include β –blocker or other agents as appropriate for compelling indications

* Unless side effects warrant change in therapy

** Consider consulting with the Clinical Center Network before adding a fifth anti-hypertensive medication

Chapter 5 – Measurements and Follow-up

5.1.1 Schedule of Follow-Up Visits

Post-randomization follow-up visit schedules for data collection do not differ by treatment group assignment. However, the visit schedule for treatment, that is achieving the BP goals, may differ by group while blood pressure goals are being met because of PRN visits not shown on Table 5.1. Additional information on treatment schedules is contained in Chapter 4 describing the SPRINT BP intervention. For data collection in both randomized groups, all participants will have post-randomization visits at Months 1, 2, 3, 6, and every 3 months thereafter. For the purpose of event ascertainment, all participants in both treatment groups will be queried regarding the occurrence of a possible event on the same schedule, specifically every 3 months.

5.1.2 Procedures by Visit

Scheduled examination components are shown by visit in Table 5.1. Assessments performed at the various visits include blood and urine collection, physical measures, and questionnaires. Assessments will be performed on the same schedule for both randomization groups. Baseline characteristics to define the patient population include sociodemographics, anthropometrics, BP, pulse, current and past medical history, concomitant medications, laboratory, dementia screening, cognitive function (subset), MRI (subset) and quality of life measurements. A physical examination is included for safety but is not standardized, and left to the discretion of the investigator.

5.2. Blood and urine collection and laboratory assays

Specific laboratory assessments (e.g. serum creatinine, fasting serum glucose, etc) are important for determining eligibility status. During follow-up, laboratory results will be used to monitor and adjust therapy in efforts to maintain blood pressure goals, assess safety (e.g. serum potassium concentrations), and to assess for study-related outcomes (e.g. deterioration of estimated glomerular filtration rate or increased protein excretion).

Serum, plasma, and urine samples will be stored for future measurements of other less traditional CV risk factors. White blood cells will be collected at baseline for DNA extraction for future genetic studies. It may prove possible to identify subgroups, defined by specific genes or genetic markers, which respond differentially to the various blood pressure treatment strategies.

5.3. Physical Examination Measures

5.3.1 Seated Blood Pressure and Pulse

Seated blood pressure and pulse are measured at each clinic visit after a rest period using an automated device or manual devises if necessary. The preferred method is the automated device as it offers reduced potential for observer biases and decreased demand on staff in terms of training and effort in data collection.

Table 5.1. Measures and Frequency

	Screening /RZ	1 mo	2 mo	3 mo	6 mo	9 mo	1 yr	Q 3 mo	Q 6 mo	2 yr	3 yr	4 yr	5 yr or Close out
Fasting blood collection													
Chemistry profile	X	X		X	X		X		X	X	X	X	X
Fasting glucose	X									X		X	
Lipid profile	X						X			X			
Serum and plasma storage	X						X			X		X	
Genomic material	X												
Urine collection													
Albumin, creatinine	X				X		X			X	X	X	X
Urine storage	X						X			X		X	
Physical measures													
Seated blood pressure, pulse, & medication adjustment	X	X	X	X	X	X	X	X		X	X	X	X
Standing blood pressure	X	X			X		X			X	X	X	X
Weight	X						X			X	X	X	X
Height	X												
ECG	X									X		X	X
Physical examination	X						X			X	X	X	X
4 meter walk (≥ 75 ONLY)	X						X			X	X	X	X
Questionnaires													
Medical history	X												
Sociodemographics	X												
Smoking/alcohol use	X						X			X	X	X	X
Concomitant medications	X						X			X	X	X	X
Adherence & Adverse Events		X	X	X	X	X	X	X		X	X	X	X
Outcomes Ascertainment				X	X	X	X	X		X	X	X	X
**Health related quality of life**													
EQ-5D	X						X			X	X	X	X
Veterans Rand 12	X						X			X	X	X	X
PHQ-9 Depression	X						X			X	X	X	X
Patient satisfaction	X						X					X	
**Health related quality of life (subsets)**													
Falls Efficacy (FESI-I)	X				X		X			X	X	X	X
Sexual Function (FSFI/IEFF)	X				X		X			X	X	X	X

MIND Questionnaires/Tests	Screening or RZ	2 yr	4 yr	Close-out*
**Dementia Screening**				
MoCA	X	X	X	(X)
Digits Symbol Coding Test	X	X	X	(X)
Logical Memory Test Story A	X	X	X	(X)
**Cognitive Battery (subset)**				
Hopkins Verbal Learning Test	X	X	X	
Trail Making Tests A and B	X	X	X	
Digit Span	X	X	X	
Boston Naming Test	X	X	X	
Modified Rey-Osterrieth Figure	X	X	X	
Verbal Fluency Animals	X	X	X	

*Dementia screen will be obtained at study close-out if there has been more than a year since the prior screen.

5.3.2 Standing (Orthostatic) Blood Pressure

Standing BP will be measured at screening, baseline, 1 month, 6 months, 12 months, and annually thereafter using the same BP device that is used to measure seated BP. After seated determinations, participants will be asked to stand. Beginning when their feet touch the floor, BP will be taken one minute later in the same arm used for the seated measurements, using the BP device. Participants will be asked after the standing determination if they had any symptoms of orthostatic hypotension during the standing BP measurement. The Coordinating Center will calculate BP change using the standing measurements minus the mean of the seated measurements.

5.3.3 Anthropometric Measurements (Weight and Height)

Body fat is a significant predictor for subclinical and clinically manifested cardiovascular disease. In addition, exercise and dietary modification with the goal of reducing total body fat may facilitate blood pressure control. Anthropometric measures gathered for SPRINT include height and weight for the calculation of body mass index.

5.3.4 Electrocardiography

A 12-lead ECG is obtained at baseline and at the 2 and 4 year follow-up visits and close-out visits to ascertain the occurrence of silent (unrecognized) MI, primarily, as well as atrial fibrillation and left ventricular hypertrophy. The baseline ECG is used to identify previous (including silent) MIs, and to identify evidence of left ventricular hypertrophy.

5.3.5 Physical Examination

The physical examination includes components of a systems-based examination deemed necessary for safety by the SPRINT site investigator. Elements of the examination to be completed may vary depending upon the health status and any symptoms reported by the participant, the time and type of visit (initial, interval, annual, final). The physical examination will not be standardized or data entered, but will be available in the source documents for each participant.

5.3.6 Four meter walk

Participants who are 75 years old or older at baseline will be asked to complete a timed 4 meter walk to assess physical function. This will be done at baseline and annually.

5.4. Questionnaires

5.4.1 Medical History

A detailed history of cardiovascular disease is collected at screening. The presence of CVD and CKD prior to entry into the study serves as an eligibility and stratification factor. Data regarding the duration of chronic kidney disease and the presence of complications are important for descriptive purposes, subgroup analyses, and prognostic analyses.

5.4.2 Sociodemographics

Information is collected during screening/baseline regarding age, race and ethnicity, gender, level of education, marital status, persons living with participants and United States (zip) postal code. These data will be used to identify eligible participants and to characterize the final study population.

5.4.3 Smoking/alcohol use

Consumption of alcohol and tobacco have important implications on cardiovascular risk, and adherence to medication regimens. Participants will be assessed at baseline for lifetime tobacco exposure, alcohol intake and binge drinking. At annual assessments, current smoking will be assessed.

5.4.4 Concomitant Medications

Information regarding the participants' concomitant non-study medication therapy is collected and documented at baseline and then reviewed and revised at annual follow-up visits. Appropriate sources for obtaining this information include participant (or significant other) report, current pharmacy action profiles, and verification of medications documented in the medical record. Although data are collected on all current therapies, emphasis is placed on concurrent antihypertensive, cardiovascular, chronic kidney disease and dementia medications as well as background risk reduction therapy such as aspirin and lipid-lowering drugs.

5.4.5 Monitoring Adherence

Adherence to antihypertensive medications will be assessed as follows:

First, an adherence scale will be administered to all participants at the baseline, 12 month and 48 month visits in order to identify low adherence.

Secondly, at every medication management visit, participants will be administered a single item to screen for low adherence. If the participant's response to this item indicates a possible problem with adherence, or if the participant is not at the appropriate blood pressure target, study personnel will address the specific issues and barriers for each study participant that may be preventing optimal adherence. In such instances, administration of the Adherence Scale (to identify reasons for nonadherence) is recommended, as is use of the materials and procedures described in the adherence binder. Details regarding the adherence monitoring procedure, scoring algorithm for the Adherence Scale and the procedures to follow when low adherence is identified, are provided in the MOP.

5.4.6 Adverse events

Adverse event ascertainment and reporting is described in chapter 8.

5.4.7 Study-related outcomes

Both randomized groups will be assessed for study related outcomes in the same way and on the same schedule. After randomization, participants will be assessed every 3 months for cardiovascular and renal outcomes. Medical records will be collected for adjudication of study outcomes as described in Chapter 9. Clinical center staff will use available resources and contact information to assess vital status annually on participants not attending study visits.

5.4.8 Health-Related Quality of Life

All participants will be assessed for the effect of interventions on health-related quality of life (see Chapter 7). HRQL data will be collected at Baseline, 12 months and annually thereafter. Depression using the PHQ-9 scale will be assessed at baseline and annually thereafter. A modified TSQM General Satisfaction subscale will be administered at baseline and at 1 and 4 years. A subset of participants will undergo additional data collection related to fall self-efficacy and sexual functioning at baseline, 6 months and annually thereafter.

5.4.9 MIND Battery: Dementia Screening

All participants will undergo a dementia screening at baseline and every 2 years thereafter. The tests will include the Montreal Cognitive Assessment (MoCA), Digit Symbol Coding test, and Logical Memory test. A subset of 2800 participants will undergo an additional comprehensive battery of neurocognitive tests conducted at baseline, Month 24, and Month 48. In addition to the neurocognitive tests, a subsample of 640 MIND participants will have a Baseline and Month 48 MRI examination.

5.5. Medications and Adherence

Adherence

As part of a central pretrial training session, all investigators and clinical coordinators will receive instruction on adherence issues. Additionally, study staff will periodically have refresher and retraining instruction in the overall adherence program throughout the trial. Also critical to maintaining good adherence is the routine discussion of participants who show problems with adherence and brain-storming about problem-solving strategies during clinic team meetings and Study Coordinator meetings and conference calls. Of particular importance is the involvement of all members of the clinic team, including clinic leadership, in adherence-related monitoring and problem-solving.

Drug Dispensing, Ordering, Storage, and Disposal

Drug Dispensing

The complexity created by the large number of medications and multiple treatment strategies employed by SPRINT requires substantial attention to the process of medication dispensing. All study medications dispensed to the participants will be labeled and identified with the study name, participant's name, medication name, strength and quantity, directions for use, and authorized prescriber's name. An emergency study-related phone number for study drug information will also appear on the label. All participants are to be verbally counseled on medication administration. Written instructions will also be provided.

Participants receive medication supplies at regularly scheduled visits in sufficient quantity to last until the next scheduled visit. Medication dispensing may occur in the intervening periods between visits in case of emergency, loss, or schedule changes. A tracking mechanism is maintained for all dispensing actions. It is recommended that authorized dispensing personnel be limited in number to assure proper adherence with established accountability and dispensing procedures.

Drug Supply Ordering

Each Clinical Site, upon completion of procedures for study initiation, will receive a standard initial shipment (determined by the Coordinating Center and prepared by the Drug Distribution Center (DDC)) of study drug supplies for the trial. It is expected that this initial shipment will suffice for a specified number of visits for a given number of randomized participants. Subsequent ordering of inventory will be managed by the site, primarily through the web-based inventory system. Sites are responsible for appropriately managing their inventory and are able to customize their medication quantities to suit the prescribing practices of their site.

The DDC in consultation with each Clinical Site sets inventory levels for each item. When an item reaches the reorder point, additional stock is automatically shipped from the DDC.

Drug Receipt and Storage

Drug shipments are sent to the Clinical Site in care of a designated staff member. The shipment is inspected for damage and its contents reconciled with the accompanying

SPRINT Shipping Notice. The inventory is logged using the established tracking mechanism. Packing slips are filed in a secure location. Any damage or discrepancies in the shipment are to be reported promptly to the DDC for corrective action. Each Clinical Site is responsible for storing the study drug supplies in a locked, secure area with limited access. Manufacturer recommendations and local policies for drug storage are followed.

Drug Disposal

Clinical Sites are authorized to destroy SPRINT stock locally, complying with any local policies and procedures. Destruction will be documented on the SPRINT Local Destruction Form, with a copy sent to the DDC. All study drugs are labeled with an expiration date. Prior to expiration, the DDC will automatically ship replacement stock based on the current electronic inventory profile. Once replacement stock is received the clinical site will destroy expired stock and document destruction as described above.

Chapter 6 – SPRINT MIND

6.1 SPRINT-MIND Overview

SPRINT-MIND is an integral part of the overall SPRINT study and all SPRINT participants will participate in one or more components of SPRINT-MIND. There are three objectives of SPRINT-MIND. The primary objective is to determine whether a strategy of intensive blood pressure lowering to target systolic blood pressure (SBP) <120 mm Hg versus a standard treatment target of 140 mm Hg will produce a greater reduction in the incidence of all-cause dementia. The second objective is to determine whether global cognitive function measured in key specific domains of cognition will decline less in persons randomized to a SBP goal of <120 mm Hg versus a standard treatment goal of 140 mm Hg in a representative sub-sample of approximately 2800 SPRINT participants. The third objective is to assess whether MRI-derived changes in brain structure differ by treatment assignment in a subset (approximately 640) of the 2800 participants.

6.2 Study Hypotheses and Aims

6.2.1 All-cause Dementia

Primary hypothesis: Over an average of 60 months, the incidence of all-cause dementia will be lower in SPRINT participants assigned to the intensive SBP treatment arm compared to their counterparts assigned to the standard SBP treatment arm. This hypothesis will be tested in all SPRINT participants.

6.2.2 Cognitive Decline

Secondary hypothesis: Over an average of 48 months, the rate of global decline in cognition measured across key domains of cognition will be lower in the intensive SBP treatment arm compared to the standard SBP treatment arm. This hypothesis will be tested in a representative subset of approximately 2800 participants enrolled in SPRINT.

6.2.3 MRI Brain Changes

The Primary brain MRI hypothesis is that over an average of 48 months, the volume small vessel ischemic disease (SVID) will be lower in SPRINT participants assigned to the intensive SBP treatment arm compared to their counterparts assigned to the standard SBP treatment arm. An additional hypothesis is that total brain volume will also be greater (thus less atrophy) in the intensively treated group. The MRI sub-study will be conducted in approximately 640 participants chosen from the 2800 subset of participants selected in 6.2.2.

6.3 Study Design

6.3.1 Study Population

We will ascertain incident all-cause dementia in all participants enrolled in SPRINT. In addition, approximately 2800 participants will be selected to receive additional cognitive assessments at baseline, 24 months, and 48 months in order to examine changes in

global and domain-specific cognition. Participants participating in the MRI substudy will, at baseline, generally be required to reside within 1.5 hours travel distance to a designated study MRI Scanner. The components of the two cognitive batteries selected to assess dementia incidence and decline in cognition are listed in Table 5.1 of Chapter 5.

6.4 Procedures for Identifying Incident All-Cause Dementia in SPRINT (see Figure 6.1).

6.4.1 Overview

A 3-step process will be used to ascertain incident cases of all-cause dementia. First, to identify possible cases of dementia a brief Cognition Screening Battery will be administered to all participants. Participants who score below the pre-designated screening cut-point for possible cognitive impairment during follow-up will be administered a more comprehensive and detailed neurocognitive test battery (the Extended Cognitive Assessment Battery) plus the Functional Assessment Questionnaire which assesses impairments in daily living skills as a result of cognitive impairments. Last, all the above available tests and questionnaire data will be submitted to a centralized, web-based system for adjudication by a panel of dementia experts who will assign final study classifications of probable dementia (PD), mild cognitive impairment (MCI) or no impairment (NI).

6.4.2 Cognition Screening Battery

A brief screening battery consisting of 3 well-validated neurocognitive tests will be administered to all participants at study randomization and repeated at years 2, 4 and at closeout (unless the participant has undergone the screening battery in the past year prior to closeout). This battery requires 15 minutes or less to administer.

Tests included in the SPRINT-MIND Cognition Screening Battery were selected because they are sensitive to detecting dementia, easy to administer and brief. They are:

1. The Montreal Cognitive Assessment (MoCA) The MoCA (Nasreddine et al., 2005) is part of the NIH Toolbox and is a reliable and valid brief screening instrument for characterizing global cognitive functioning. It has been used previously to screen for dementia and MCI with sensitivity of >85%. The MoCA has several sub-scales that can be used to characterize more specific cognitive functions.

2. Digit Symbol Coding test (DSC) The DSC ((Wechsler, 1996b; Wechsler D., 1981) is a sub-test of the Wechsler Adult Intelligence Scale-IV. It measures psychomotor speed and working memory. The DSC and its predecessor the Digit Symbol Substitution test have been extensively used and normed.

3. Logical Memory test (LM): The LM test is a sub-test of the Wechsler Memory Scale-IV(Wechsler, 1996a; Wechsler, 1996a). It measures episodic verbal memory and has extensive normative data. Episodic verbal memory is an especially sensitive predictor of early Alzheimer’s dementia and amnestic MCI.

The sensitivity and specificity of the Cognition Screening Battery to detect participants with poorer cognitive function will be evaluated on an ongoing basis during the trial by using available baseline cognition data from SPRINT. We estimate 20-25% of participants will trip the battery (see SPRINT MOP for specific battery cut-points) and receive a brief assessment of the impact of their cognitive function on daily life (the 10 item Functional Assessment Questionnaire). At the years 2 and 4 follow-up, participants who trip the screening battery will also be administered the SPRINT Extended Cognitive Assessment Battery for adjudication of incident dementia. In order to achieve the 20-25% target, various cut-points for the Cognition Screening Battery will be compared and adjustments will be made to maximize study efficiency and economy during the trial.

6.4.3 SPRINT Extended Cognitive Assessment Battery

The Extended Cognitive Assessment Battery will provide a more comprehensive and detailed assessment of specific major cognitive functions (memory, language, visuospatial skills, executive function) that are necessary for classification of dementia and for detecting domain-specific changes. During follow-up years 2 and 4, participants scoring in the impaired range on the Cognition Screening Battery will be administered the Extended Cognitive Assessment Battery at their next scheduled visit (typically a blood pressure assessment and medication distribution visit). This entire battery requires less than 40 minutes including scoring and data entry and less than 30 minutes in persons without significant memory impairment.

The neurocognitive tests comprising the Extended Cognitive Assessment Battery are:

1) The Hopkins Verbal Learning Test (HVLT) (Brandt and Benedict, 2001)**:** A measure of episodic verbal learning and memory, this test is a 12-item list learning and memory task with immediate recall, delayed recall and recognition components.

2) The Trail Making Test: Parts A and B (Reitan R.M., 1958)**:** The Trail Making Test (TMT) is a two-part test measuring speed of processing and executive function. The times to complete Part A and Part B are the primary measures of interest.

3) Digit Span test (Wechsler D., 1981): The Digit Span test (DST), a subtest of the Wechsler Adult Intelligence Scale-IV, requires the participant to recite gradually increasing series of digits forward and backward. The DST measures concentration and working memory.

4) The Boston Naming Test (Kaplan E et al., 1983) The Boston Naming Test (BNT) is used to assess language function. The participant is asked to name familiar objects from simple drawings. The number of correctly identified objects is the variable of interest. We will use a validated short form that includes 15 items.

5) The Modified Rey-Osterrieth Complex Figure (mRey-O). (Saxon, 2003) The mRey-O measures of visuospatial and visuomotor function and non-verbal memory by having participants copy and reproduce from memory a multi-component figure. For ease of use and scoring reliability, the mRey-O figure will

be faxed to the CC and scored centrally.

6) Category Fluency-Animals. The animal fluency task requires the participant to spontaneously name as many animals as possible in 60 seconds. It provides an assessment of semantic fluency.

6.4.4 Additional measures

Functional Assessment Questionnaire (FAQ). Since impairment of daily functioning is required for a classification of dementia, we also will administer, either locally (by certified SPRINT clinic staff) or centrally (by certified SPRINT staff from the coordinating center), the FAQ, a 10-item, validated questionnaire assessing functional status (Pfeffer and others, 1982), to a person previously designated by the participant who is familiar with his/her current abilities. Administration of the FAQ will be limited to participants whose Cognition Screening Battery indicates possible impairment. Items assess functions like managing money and remembering names of familiar persons.

6.4.5 Alternative cognitive assessment.

If participants cannot come to the clinic for their follow-up exams or if they reside in nursing homes, study personnel will complete either a home or nursing home visit. Technicians conducting the home visit must be MIND certified. The Screening Battery will be administered and if the participant scores below the cut-point, the Extended Battery will also be administered.

Telephone assessment of general cognitive function is now standard practice in many large trials assessing for dementia outcomes. For SPRINT participants unable to receive a face-to-face cognitive assessment by certified SPRINT staff at their local clinic, a telephone assessment of cognition status to assess for incident dementia will be performed either locally or centrally by SPRINT certified staff. The components of the **phone interview** are:

Modified Telephone Interview for Cognitive Status (TICS-M), a validated instrument requiring <10 minutes (Welsh, 1993)
Oral Category Fluency-Animals
Oral Trail Making Test (Ricker et al., 1996)
FAQ to a contact

For participants unable to be interviewed in-person or by phone, a previously identified contact will be administered:

The Dementia Questionnaire (DQ). The DQ (Ellis , 1998;Kawaset al, 1994) is a semi-structured interview designed for a knowledgeable proxy to provide information regarding the participant's cognitive and behavioral functioning and other health information needed to make a diagnosis of dementia and MCI and to identify causes of cognitive impairment. Again, it will only be administered in the absence of an in-person or phone assessment and may be performed either by local or central staff who are SPRINT certified.

.

6.5 Adjudication of Dementia, MCI or No Impairment

A primary goal of SPRINT MIND will be to determine the incidence of all-cause dementia in SPRINT and its relation to the treatment assignment. Final classification (Dementia, MCI or No Impairment) will be made by a panel of experts consisting of neurologists, geriatricians, psychiatrists and neuropsychologists with recognized expertise in dementia blinded to study assignment and blood pressure data. Data used in the adjudication will include all available cognitive test data (SPRINT Cognition Screening Battery, SPRINT Extended Cognitive Battery), functional status assessments (FAQ or DQ) and additional data including demographic information and medical historyEach suspected case identified by our scoring criteria (see 6.4) will be randomly assigned to two members of the Adjudication Committee for review. Adjudicators will independently review all the available data via a web-based system before recording their classification-Dementia, MCI or No Impairment. Each adjudicator will be masked to the other's classification and to the participant's treatment assignment. If the two adjudicators' classifications agree, then the classification will become final. Disagreements will be resolved at periodic face-to-face meetings or by phone conferences between adjudicators and additional members of the Adjudication Committee until consensus is achieved. These procedures have been successfully used by our team in other large clinical trials including the Gingko Evaluation of Memory Study (GEMS) (DeKosky et al, 2008) and the Women's Health Initiative Memory Study (WHIMS) (Shumaker et al, 2004).

Participants classified as having dementia will no longer be assessed for cognitive function. Those not classified as having dementia will continue to receive regularly scheduled cognitive assessments with the screening and extended cognitive batteries when indicated.

6.5.1 Diagnostic Criteria for Dementia

Criteria used for identifying dementia will be those described in the Diagnostic and Statistical Manual of the American Psychiatric Association-Fourth Edition (DSM-IV). These are:

- Significant decline in memory and at least one additional cognitive domain; and
- Significant functional impairment due to cognitive problems; and
- Cognitive deficits are not due to obvious reversible causes such as acute illness, metabolic disturbances, infections, mood disorders or substance-induced conditions; and cognitive deficits do not occur exclusively during the course of delirium.

No attempt to classify dementia subtype will be made.

6.5.2 Diagnostic Criteria for MCI

While not a primary or secondary outcome, MCI syndrome is important because of its relevance to dementia. MCI represents a transitional state between no cognitive impairment and dementia and specific subtypes of MCI are highly predictive of subsequent dementia. Thus, identifying MCI will provide valuable information about pre-dementia cognitive impairment related to the SPRINT intervention. Criteria to be used for identifying mild cognitive impairment syndrome are those described by Winblad et al., which are:

- Observation by participant or proxy of cognitive decline; and
- Deficit in performance in one or more cognitive domains; and
- Absence of significant functional impairment attributable to cognition; and
- No diagnosed dementia

MCI will be further sub-classified into 4 categories using criteria adapted from Winblad, et Al. (Winblad et al, 2004) as follows:

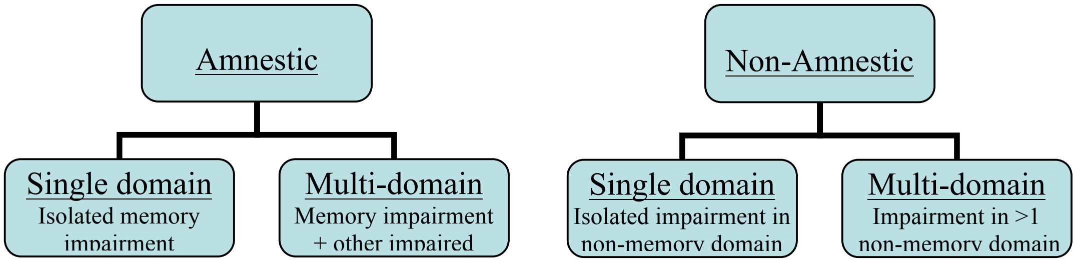

Specific cognitive tests in the Cognition Screening Battery and the Extensive Cognitive Assessment Battery will be used to subtype adjudicated cases of MCI.

6.6 Baseline classification of cognitive status:

Rare cases of dementia, where the participant or their personal physician are unaware of the diagnosis, may be identified during baseline cognitive testing. In participants scoring below the cut-point on the Screening Battery, we will administer the FAQ to a contact in order to determine the presence of impaired daily function related to cognition (see 6.4.2).

6.7 Definition of Cognitive Change Over Time Outcome (Extended Cognitive Assessment Battery Sub Sample).

Each test score from the Cognition Screening Battery and the Extended Cognitive Assessment Battery will be used to measure decline in cognitive function. The primary outcomes will be composite scores for two domains: 1) Memory, consisting of the Hopkins Verbal Learning Test, Logical Memory and the Modified Rey Osterrieth Figure, and 2) Processing Speed, consisting of Trails Making Tests and Digit Symbol Coding Test. Prior to analysis of this outcome, we will review the science related to summary scores for cognitive function and may make modifications which will be specified prior to initiation of the analysis.

6.8 Quality Control and Training

At each clinical site, at least one person will be identified to serve as the trained and certified cognitive technician. Technicians will be trained during a central, intensive training session held in conjunction with the overall SPRINT training. Training will include review of the MIND protocol and procedures for administration of the test batteries, demonstrations of each component of the SPRINT MIND test batteries, and opportunities to practice with feedback from trainers. When a level of competence is attained, technicians will receive certification and approval to administer the test batteries to SPRINT participants. During the course of the study as additional staff is

needed, certified technicians will train new technicians and submit materials to the MIND Coordinating Center for review. Technicians will be recertified throughout the course of the trial by: 1) review of video or audio taped administrations; 2) observing web-based administrations and responding to specific questions. Technicians will be encouraged to communicate questions or problems to the SPRINT MIND Coordinating Center.

Figure 6.1.

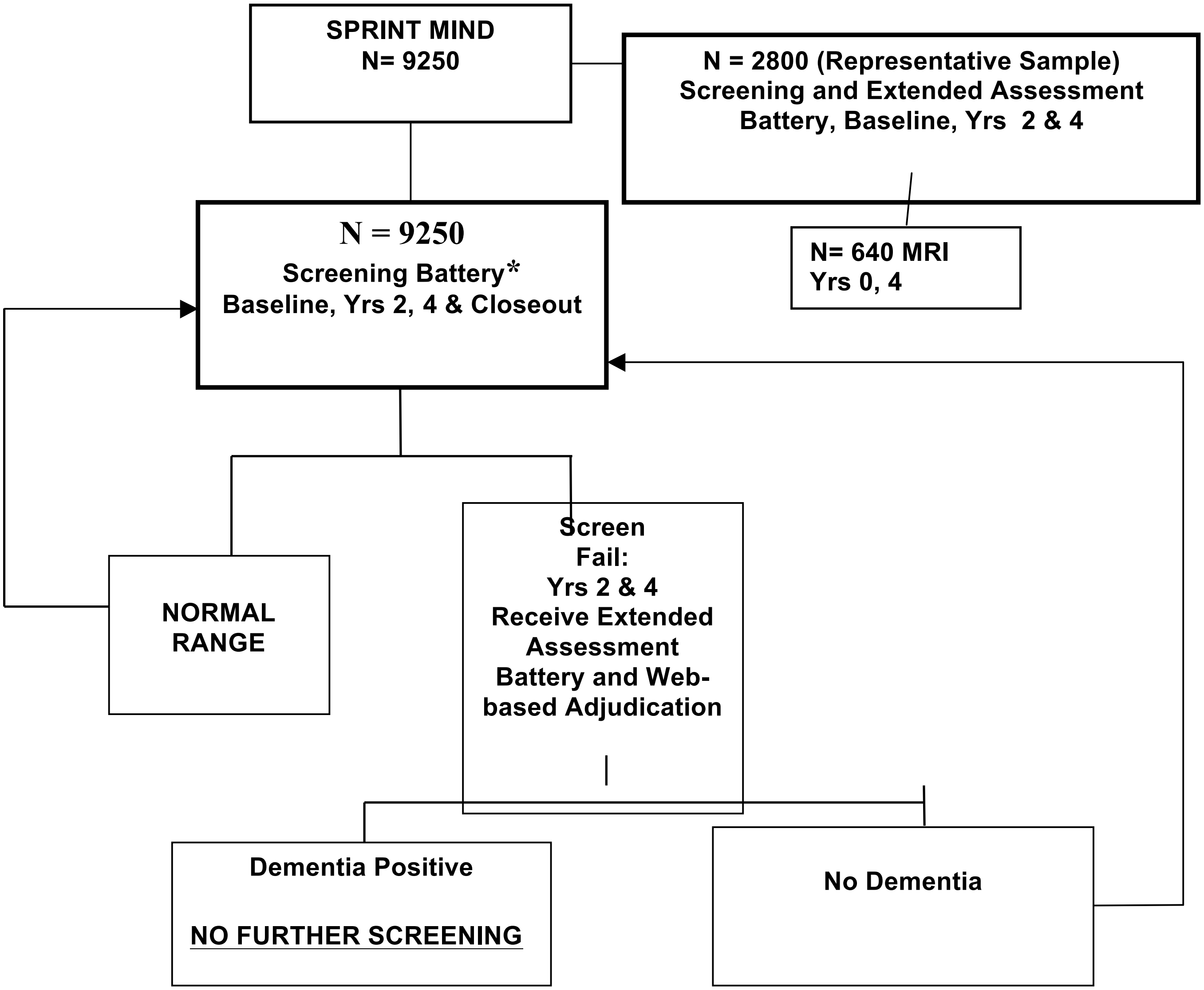

*At baseline, participants scoring below cutoffs specified during trial will also receive the FAQ.

Chapter 7 – Health-Related Quality of Life and Economic Analyses

7.1. Introduction

In addition to the cardiovascular, renal and cognitive outcomes, SPRINT is well poised to examine differences in health-related quality of life (HRQL) as a result of its blood pressure interventions. Differences in HRQL may affect adherence, and thus the effectiveness of the two interventions. It is also reasonable to anticipate that in some cases, the intensive arm may result in diminished HRQL relative to the standard arm due to a number of factors:

- side effects of specific medications or increased numbers and/or doses of medications required to achieve the <120 mm Hg goal,
- increased occurrence of hypotensive symptoms, which may not only result in higher rates of falls and fractures, but also an increased fear of falling which may limit the participant's perceived ability to engage in activities of daily living, and/or
- reduced perfusion pressures and medication side effects which may contribute to erectile dysfunction in men, and possible sexual dysfunctions in women.

On the other hand, the intensive arm may result in improved general HRQL versus the standard arm due to reduced number of medical events and more favorable physical and cognitive function. The effects of the two interventions upon HRQL are further nuanced by the possibility that some participants in either treatment arm may adjust to decrements in health status by changing their internal perception of favorable HRQL, known as "response shift".

There may also be potential health cost tradeoffs of the intensive versus standard treatment. While the intensive arm is anticipated to result in higher short-term costs due to more frequent office visits and greater medication use, this arm may also result in lower long-term costs from event-related hospitalizations and other medical costs if the treatment approach is efficacious in reducing these medical events. Assuming the primary outcomes are as hypothesized, examining the HRQL and cost-effectiveness of the intensive and standard treatment arms will be important determinants of the potential adoption of the intensive BP control in clinical practice, and will be informative in identifying subgroups of patients for whom intensive or standard BP control is most appropriate.

7.2. Hypotheses

7.2.1 HRQL Hypotheses

The hypotheses generated for the HRQL measures are:

- Overall HRQL (Entire sample, Veterans RAND-12) Intensive control of blood pressure compared to standard control will result in worse HRQL at the 1-year assessment, but better HRQL at the 5-year assessment. The effect will be greater in those with lower baseline HRQL and greater number of comorbid conditions at baseline.

- Falls Self-efficacy (Subsample, Falls Self Efficacy Scale) Intensive control of blood pressure compared to standard control will result in less favorable fall-related self-efficacy at the 1-year assessment. The effect will be the greater in older participants, those with lower baseline HRQL, and those with a greater number of baseline comorbid conditions. By Year 5, intensive control of blood pressure will result in more favorable fall-related self-efficacy compared to standard control.

- Sexual function (Subsample, Modified Female Sexual Function Assessment /International Index of Erectile Function) Intensive control of blood pressure compared to standard control will decrease sexual function among men and women participants at one year. By year 5, the intensive treatment participants will report more favorable sexual function compared to participants in the standard treatment.

7.2.2 Cost-Effectiveness Hypotheses

The primary hypotheses generated for the economic and cost-effectiveness analyses are:

- Intensive control of blood pressure compared to standard control will result in higher healthcare costs and utilization in the first year due to the greater number of office visits, medications, and lab tests likely required to achieve the intensive control targets.

- Intensive control of blood pressure compared to standard control will result in lower healthcare costs and utilization over the study period due to decreased events and related health costs among intensive control participants.

- The incremental cost-effectiveness ratio will be $\leq$\$100,000/Quality Adjusted Life Years (QALY) gained when compared to the standard intervention.

7.3. Health-Related Quality of Life Measures

7.3.1 Rationale for Selection

The SPRINT HRQL instruments were selected based upon the following criteria: (1) inclusion of the major dimensions shown in the literature to be affected by hypertension and its treatment; (2) brevity; (3) responsiveness to treatment-related changes, and (4) appropriateness for the age range, racial/ethnic diversity, and anticipated medical conditions of the participants in SPRINT.

To reduce participant burden, some HRQL instruments will be administered to the entire SPRINT sample, while others will be administered only in a subsample of participants. All HRQL instruments will be self-administered unless participants require assistance due to sensory, motor, or cognitive deficits in which case the instruments will be administered by clinic staff or family/friends accompanying the participant to the clinic visit. For Spanish-speaking participants, Spanish versions of all HRQL instruments will be administered to participants at all assessment points who indicate at baseline that

they do not have sufficient written English fluency to complete the instruments in English.

7.3.2 Health-Related Quality of Life (HRQL) Measures

Veterans RAND 12-item (VR-12) questionnaire. The VR-12 is a shorter version of the VR-36 (which is derived from the SF-36). Changes of the VR-12 relative to the SF-12 have lowered the floor and ceiling, improved the distributional properties, increased reliability, and improved discriminant validity of the physical and mental health summary scores. Validated conversion formulas allow for direct comparisons to prior studies using the SF-36 or SF-12. The VR-12 will be administered to all SPRINT participants at baseline and at annual visits thereafter.

Fall Self-Efficacy Scale International (FES-I) The FES-I, shortened version, consists of seven items which the respondent answers on a 1-4 scale, indicating level of concern for falling. The activities are getting dressed or undressed, taking a bath or shower, getting in or out of a chair, going up or down stairs, reaching for something above your head or on the ground, walking up or down a slope, and getting out to a social event. An evaluation of the Short FES-I found good internal and 4-week test-retest reliability. The correlation between the Short FES-I and the FES-I was 0.97. The Short FES-I will be administered among a subsample of SPRINT participants.

International Index of Erectile Function (IIEF) The IIEF-5 is the 5-item short form of the original 15-item IIEF, and was developed specifically for use in clinical settings to supplement physical examination and patient history. IIEF-5 scores can be classified into the following categories; severe erectile dysfunction (ED), moderate ED, mild to moderate ED, mild or no ED. Scores less than 21 have 98% sensitivity and 88% specificity for the presence of ED. The IIEF-5 will be administered in a male subsample of SPRINT participants.

Female Sexual Function Assessment (FSFI) The FSFI is a 19-item survey that assesses female sexual function over the past four weeks in 6 domains (desire, arousal, lubrication, orgasm, satisfaction, and pain). Utilizing recently proposed modifications to the FSFI, participants not sexually active over the past four weeks would complete only 4 items, substantially reducing respondent burden. The FSFI has high internal consistency (Cronbach alpha > 0.8). This assessment will be administered in a female subsample of SPRINT participants.

Patient Satisfaction (Bharmal and others, 2009) A modified Treatment Satisfaction Questionnaire for Medication (TSQM) General Satisfaction subscale will be administered at baseline (based on current blood pressure medications being taken, if any) and at 1 and 4 years. This corresponds with the administration of the Morisky Adherence scale, which will allow for analyses of the relationship between satisfaction and adherence at these time points.

Patient Health Questionnaire-9 (PHQ-9) The PHQ-9 is a self-report measure of depression that has been recommended by the AHA Advisory Panel on Depression and Coronary Heart Disease, has a low response burden (9 items; 2-3 minutes to complete), excellent reliability, and good sensitivity and specificity with depression diagnoses. This assessment will be done at baseline and annually on all participants.

7.3.3 Health State Utility Measures

EQ-5D is a self-administered 5-item instrument including mobility, self-care, usual activities, pain/discomfort and depression. There are three responses to each question (no, moderate, or severe limitations). This commonly used measure of health utilities will be used to convert quality of life and health status into quality adjusted life-years (QALYs) for cost-effectiveness analysis. The EQ-5D will be administered to all participants at baseline and annually thereafter.

7.4. Cost-Effectiveness Assessment

7.4.1 Rationale

It is expected that the intensive therapy for hypertension will not only reduce cardiovascular events but will be more cost-effective over the long-term. The two primary measures of cost-effectiveness are the incremental cost per QALY and life-year gained. The primary cost-effectiveness hypothesis is that the intensive blood pressure treatment will be cost-effective as compared to the standard treatment. This question will be addressed by conducting incremental cost-effective analyses in which the net costs and net effectiveness of intensive therapy defined by the main trial to standard therapy will be calculated and expressed as a series of ratios.

For QALYs, the cost-effectiveness hypothesis is that the ratio of costs per QALY (as measured by the EQ-5D) will be significantly less (i.e., more favorable cost-effectiveness) for the intensive intervention than for the standard intervention. Costs will be discounted to weigh future costs less heavily than present ones.

7.4.2 Effectiveness

The primary endpoints defined by the main trial are considered as primary outcome measures for this economic evaluation. The primary effectiveness measures will be life-years gained and QALY gained. The measure of life-year gained is determined by the difference in number of life–years between intensive therapy and standard therapy. QALYs adjust life-years gained by the quality of the participant’s overall HRQL during these life-years gained.

7.4.3 Costs

All direct medical costs associated with treatment of hypertension and its complications and costs for treating adverse effects of the therapy will be considered. These costs will include costs of inpatient care, outpatient care, medications, medical equipment, supplies, laboratory tests, and professional services. The participant's costs such as waiting time, transportation, lodging, and informal care arising from the disease will not be included. Likewise, opportunity costs of premature death, productivity loss, and long-term disability will not be considered in this study.

7.4.3.1 Cost Data Collection

Hospitalizations are the primary cost drivers in most cost-effectiveness analyses, and SPRINT has proposed obtaining hospitalization events via multiple sources. Patient report of hospitalizations, along with emergency department (ED) visits, stays in rehabilitation facilities, and day-surgery admissions, are obtained every 3 months during scheduled SPRINT study visits. Discharge summaries and other pertinent records (including reason for hospitalization and length of stay) will be obtained from hospitalizations, Emergency Department visits, rehabilitation stays, and day-surgery admissions related to outcome events and potential adverse events (including cardiovascular, renal, and cerebrovascular disorders; dementia; falls) which will constitute many of the admissions that might be expected to differ by arm. Because of the large proportion of VA and Medicare patients in SPRINT, we also will be able to determine hospitalizations, dates of admission, length of stay, and reason for admission via Medicare and VA databases for those hospitalizations for which we do not have discharge summaries. For the limited number of remaining patient reports for which we have neither discharge summaries nor database information, we will perform regression analyses of reported vs. actual length of stay and costs for all those with such data to estimate the costs of the undocumented hospitalizations. Cost estimates for hospitalizations will be based on DRG-specific Medicare cost weights. For professional costs associated with hospitalizations we plan to obtain these costs from Medicare and VA databases as available in a subsample and use these data to estimate professional costs for the entire sample based on these subsample analyses. We will also explore whether these databases allow us to obtain costs associated with ED visits, stays in rehabilitation facilities, and day surgery admissions.

7.4.3.2 Intensive and Standard Therapy Non-Research Costs

For medications, we plan to use study medication logs to obtain the medications prescribed by the study. This log also includes blood pressure lowering medications prescribed by other healthcare providers. Medication costs will be estimated using median wholesale price. We will obtain information on non-study prescribed medications (concomitant medications) from participants annually and will estimate costs for these medications based on the most commonly used doses in clinical practice. We will not obtain cost data on non-study related labs, as this source of utilization is not expected to differ by group. To estimate non-research related costs for the SPRINT office visits, we plan to obtain estimated CPT codes (minus research-specific activities) from clinic staff for a random subset of these visits to estimate costs via Medicare payment rates. Non-study outpatient visits will not be obtained but will be estimated with non-study medication costs by age using national health care expenditure data.

7.4.3.3 Data Analysis for Cost-Effectiveness

Two methods of cost-effectiveness analysis (CEA) and cost-utility analysis (CUA) will be used in the economic evaluation. The ratios of cost to outcome derived from CEA/CUA are used to compare cost-effectiveness among treatment strategies. An incremental cost-effectiveness ratio (ICER) will be calculated, which provides a summary of the cost-effectiveness of one intervention relative to the other.

The basic formula to calculate incremental CEA ratio and CUA ratio of a specific treatment A relative to the reference treatment B is presented as following:

$$ICER_{CEA} = \frac{(\text{Mean Cost}_{\text{treatment A}} - \text{Mean Cost}_{\text{treatment B}})}{(\text{Mean Effect}_{\text{treatment A}} - \text{Mean Effect}_{\text{treatment B}})}$$

$$ICER_{CUA} = \frac{(\text{Mean Cost}_{\text{treatment A}} - \text{Mean Cost}_{\text{treatment B}})}{(\text{Mean QALY}_{\text{treatment A}} - \text{Mean QALY}_{\text{treatment B}})}$$

The ratio of incremental cost to incremental effectiveness represents cost-effectiveness of the intensive BP treatment. Bootstrap methods will be used to calculate confidence intervals for cost-effectiveness ratios. All costs will be adjusted to the baseline year using the medical component of the Consumer Price Index. Future costs and outcomes will be discounted by 3%. Estimates of utilization over time will be adjusted for the presence of censored data with variable follow-up. Sensitivity analysis will explore the effect of correlations between costs and outcomes, which will also be empirically examined in the cost and outcome data.

QALYs will be calculated by summing the area under each individual's QALY curve (constructed by plotting the EQ-5D scores for each interview during follow-up). The estimates of mean differences in costs and outcomes – which will be used to create net health benefits and the cost per QALY ratios -- will be derived from multivariable regression analyses. For the evaluation of the difference in costs, the dependent variable in the regression will either be costs or the natural log of costs (determination of the form of the dependent variable will be based on statistical tests of its distribution). If the dependent variable used in the analysis is the log of costs, a smearing retransformation will be used to estimate the absolute difference in costs between the treatment groups.

Chapter 8 – Safety Monitoring and Reporting

8.1 Introduction

The SPRINT trial is testing whether lowering SBP to a goal of <120 mm Hg results in better outcomes than a goal of <140 mm Hg in patients at risk for CVD events. SPRINT is not a study of specific anti-hypertensive agents. All antihypertensive agents provided by the trial or recommended by SPRINT have been approved by the Food and Drug Administration (FDA) and are routinely prescribed for lowering blood pressure.

Patient safety will be carefully monitored in SPRINT. Each participating investigator has primary responsibility for the safety of the individual participants under his/her care. In addition, an independent Data and Safety Monitoring Board (DSMB) will have primary responsibility for monitoring the accumulating study data for signs of adverse trends in morbidity/mortality and treatment-related serious adverse events.

8.2 Participant population

Participants enrolled in SPRINT have elevated risk for CVD outcomes. Inclusion and exclusion criteria for SPRINT were set in order to maximize safety while facilitating inclusion of a trial population at risk for the major trial outcomes. Exclusions are outlined in Section 3.1.

Potentially Vulnerable populations: The SPRINT population includes a significant proportion of older adults (>75 years), some of whom may become cognitively impaired during the course of the trial. Thus, participants are asked to identify a contact person at the time of enrollment that can provide information about the participant as it relates to the study. In addition, participants with CKD may need care coordination or referral to a nephrologist during the study. Various management issues in patients with eGFR values lower than 30 ml/min/1.73m^2 may arise including dietary issues and the effects of CKD on pharmacokinetics, pharmacodynamics and side-effects of various drugs. All participants, including those with CKD, will be managed according to current national guidelines. If patients with this level of renal impairment are not already followed by a nephrologist and the investigator feels it is needed, he/she will coordinate with the participant's primary care physician regarding the recommendation for renal follow-up.

8.3 Safety Monitoring

Several types of safety issues and serious adverse events may occur in SPRINT and participants will be monitored for these regularly throughout the study.

8.3.1 Expected Events:

The potential adverse effects of the blood pressure drugs used in SPRINT have been well documented. For example, electrolyte abnormalities (hyponatremia or hypokalemia are known to be associated with diuretics; hyperkalemia and short-term decline in GFR with RAAS blockers, hyperkalemia with potassium-sparing drugs; as well as bradycardia with beta blockers and calcium channel blockers). Participants will be monitored

routinely with interviews, vital signs, targeted physical examination and laboratory tests to ensure safety (Chapter 5, Table 5.1). In addition, site clinicians may also obtain local labs and ECG's if safety is a concern at non-scheduled intervals. Clinical alerts are generated when safety parameters are exceeded. (Table 8.1). Expected events are not considered serious adverse events (SAEs) unless they meet criteria for an SAE (see 8.3.2).

Table 8.1 Clinical Safety Alerts

**Measure**	**Alert Value**
Serum sodium	< =132 or >150 mEq/L
Serum potassium	<3.0 or >5.5 mEq/L
Serum creatinine	Increase by at least 50% to a value >=1.5 mg/dL since the last study lab (usually 6 months apart).
Heart rate	<40
ECG	acute MI, complete heart block, or bradycardia <40 bmp
PHQ-9 (depression screen)	Positive response to question on suicidal ideation
Dementia Assessment	Adjudicated dementia

8.3.2 Adverse Events and Serious Adverse Events

An adverse event (AE) is defined as any untoward or unfavorable medical occurrence in a human subject, including any clinically significant abnormal sign (for example, abnormal physical exam or laboratory finding), symptom, or disease, temporally associated with the subject's participation in the research, whether or not considered related to the subject's participation in the research. The burden of collecting and reporting data on every possible AE in SPRINT is excessive and side effects from the drugs to be used in SPRINT have been well defined in previous studies. Therefore, in SPRINT, sites will report all serious adverse events and selected AEs to the Coordinating Center.

Consistent with NHLBI guidelines and OHRP policy, SAEs are adverse events that meet any of the following criteria:

- fatal or life-threatening,
- result in significant or persistent disability,
- require or prolong hospitalization,
- result in a congenital anomaly/birth defect, or
- are important medical events that investigators judge to represent significant hazards or harm to research participants and may require medical or surgical

intervention to prevent one of the other outcomes listed in this definition (e.g. hospitalization, death, persistent disability).

Any adverse event that meets any of these criteria will be documented and reported as a serious adverse event. In addition, a select list of other important events (see manual of procedures for details and definitions), regardless of whether they resulted in hospitalization, will also be considered SAEs in SPRINT, including:

- Injurious falls
- Syncope
- Unexpected events for which the investigator believes that the SPRINT intervention caused the event or contributed to the immediate cause of the event

Participants will be queried for SAEs and selected AEs at quarterly clinic visits.

8.3.3 Modification of treatment in response to safety concerns

SPRINT is testing two different SBP treatment goals. The study physician may add, increase or reduce the dose, stop, or change antihypertensive drugs in the interest of participant safety. Depending on the situation, the change may be temporary or permanent. Situations that may require temporary reduction or elimination of a study medication include: side effects, worsening congestive heart failure, acute kidney injury, symptomatic hypotensive episodes, and other illnesses. Orthostatic hypotension is usually related to specific drug classes and not BP level per se and thus should NOT usually alter target blood pressure goals. The MOP contains a section on management of symptomatic orthostatic hypotension.

8.4 Safety Reporting

8.4.1 Clinical Safety Alerts

Clinical Safety Alerts (section 8.3.1. and Table 8.1) are provided to the site clinician for his/her action. When any laboratory measurement attains a defined alert level, the Central Laboratory will immediately notify the clinical site and the CCN. Site clinicians may also obtain local labs if safety is a concern at non-scheduled intervals. Site clinicians are responsible for timely review of all labs drawn locally and when central lab results become available. ECGs will be done at specified visits and read by the ECG reading center. However, if a participant has one of a short list of abnormalities (reported on the ECG by the machine), such as acute MI, complete heart block, or bradycardia <40 beats/minute, the ECG will be reviewed by the site clinician immediately (see ECG section of the SPRINT MOP).

8.4.2 Serious Adverse Events

At each quarterly visit, SPRINT staff will specifically query participants for serious adverse events. In addition, information on serious adverse events may also be reported to study staff spontaneously by participants through telephone calls or emails between study visits. In addition to local reporting requirements, all serious adverse events will be recorded by clinic staff and forwarded to the CC Medical Safety Officer **within 72 hours** of knowledge of the event. SAEs will be collected and reported from screening to

the end of the study follow-up period for an individual participant. SAEs will be followed until resolution, stabilization, or until it is determined that study participation is not the cause.

The Coordinating Center will be responsible for timely reporting to the NIH and the DSMB. The Coordinating Center will provide reports of serious adverse events for review by the DSMB at their meetings.

8.5 Data Safety Monitoring Board

A **Data Safety Monitoring Board (DSMB)** is established, with responsibility to monitor all aspects of the study. The **Medical Safety Officer** reports to the DSMB for issues related to participants' safety. This independent Data and Safety Monitoring Board will be established to monitor data and oversee participant safety. Members will be appointed by the NHLBI to provide oversight of the trial and its ancillary studies. The SPRINT DSMB may include experts in cardiovascular medicine (particularly hypertension), kidney disease, clinical trials, geriatrics, biostatistics, quality of life, cost effectiveness, cognitive function and other areas as needed. DSMB participants include the Steering Committee Chair and Vice-Chair, CC PI and senior staff, and representatives from the NHLBI and other NIH sponsors. The DSMB normally meets twice a year to monitor safety, to advise the NHLBI about study progress and performance, and to make recommendations to the NHLBI regarding study continuation and protocol changes. In addition, the CC may provide data to the DSMB Chair to ensure early identification of any major adverse outcomes of therapy. The DSMB has the responsibility to recommend to the NHLBI whether the trial should continue, whether the protocol should be modified, or whether there should be early termination. The DSMB will provide reports to the NHLBI through the Executive Secretary, who will be appointed by the NHLBI. Recommendations by the DSMB must be approved by the NHLBI prior to implementation.

Chapter 9 – Clinical Outcome Measures

9.0 Outcomes

This chapter describes the SPRINT primary and secondary clinical outcomes. Clinical events occurring during follow-up will be ascertained primarily through surveillance of self-reported events, laboratory, and ECG data collected by the study and classified by members of the Morbidity and Mortality subcommittee masked to treatment assignment. Additional sources, including searches of the National Death Index (NDI), will also be used to augment follow-up data.

9.1 Primary Outcome

The primary outcome measure for SPRINT will be major CVD events, defined as the composite endpoint comprised of the first occurrence of a

- fatal or non-fatal myocardial infarction (MI),
- non-MI acute coronary syndrome (non-MI ACS),
- fatal or non-fatal stroke,
- fatal or non-fatal heart failure (HF), or
- death attributable to cardiovascular disease (CVD).

MI and non-MI ACS are defined in Section 9.1.1; stroke is defined in Section 9.1.2; HF is defined in Section 9.1.3, and CVD death is defined in Section 9.1.4. The SPRINT Manual of Procedures contains the full details of these definitions.

9.1.1 MI and Non MI ACS

9.1.1.1 MI: Defined as the death of part of the myocardium due to an occlusion of a coronary artery from any cause, including spasm, embolus, thrombus or rupture of a plaque. SPRINT will use standard case definitions for both fatal and nonfatal MI based on the combination of symptoms, elevation in biomarkers, and/or ECG findings. The algorithm for classifying MI includes elements of the clinical presentation (signs and symptoms), results of cardiac biomarker determinations, and ECG readings, and is based on a 2003 Scientific Statement (Luepker and others, 2003). The definition includes MI that occurred during surgery/procedure and MI aborted by thrombolytic therapy or procedure. SPRINT adjudicators will be guided by specific, pre-specified definitions and operational rules. Adjudicators will use their clinical interpretation of the ECGs and other available evidence for the event to classify MI cases as definite, probable, or possible, with all included in the primary outcome (Luepker and others, 2003). MI will be ascertained both from adjudication of hospital records for clinical events and also from the finding of new significant Q waves from the standardized interpretation of the study visit-obtained ECG (silent or unrecognized MI). MIs that present clinically will include Q wave, ST elevation and non-ST elevation infarctions (segment elevation myocardial infarction (STEMI) and Non-ST Segment elevation myocardial infarction (NSTEMI), as well as aborted MI and post-intervention MI.

9.1.1.2 Non-MI ACS: Defined as hospitalization for evaluation and treatment of an accelerating or new symptom pattern consistent with coronary artery insufficiency without meeting the definition of MI, but requiring evaluation to rule-out MI on clinical presentation. Non-MI ACS in SPRINT will also require objective findings of coronary ischemia, including any of the following: history of previous catheterization with

significant obstruction or previous revascularization; significant obstructive lesion(s) on coronary catheterization during index hospitalization and/or intervention for revascularization; ischemic ECG changes or imaging findings on exercise or pharmacologic stress testing associated with the index hospitalization; or resting ECG findings consistent with ischemia occurring with symptoms during the index hospitalization.

9.1.2 Stroke

9.1.2.1 Stroke: SPRINT will use standard case definitions for both fatal and nonfatal stroke. Stroke will be defined based on all available data, including symptoms and signs, imaging of the brain and large vessels, and cardiac testing, e.g., echocardiography. Adjudicators will use their clinical judgment based on the available evidence to classify each case, and will be guided by pre-specified definitions and operational rules. Stroke is GENERALLY defined as neurological deficit of cerebrovascular cause that persists beyond 24 hours or is interrupted by death within 24 hours. (World Health Organization, 1978 Cerebrovascular Disorders (Offset Publications). Geneva: World Health Organization. ISBN 9241700432. Exclusionary conditions for stroke include major brain trauma, intracranial neoplasm, coma due to metabolic disorders or disorders of fluid or electrolyte balance, peripheral neuropathy, or central nervous system infections. Stroke will be classified as brain infarction, subarachnoid hemorrhage, intraparenchymal hemorrhage, other hemorrhage, other type, or unknown type. In SPRINT, brain infarction (ischemic stroke) is defined as a new lesion detected by computed tomography or magnetic resonance imaging or, in the absence of a new lesion ON AVAILABLE IMAGING, clinical findings consistent with the occurrence of stroke that lasted for more than 24 hours (N Engl J Med 2001;345:1444-51). Brain infarctions will be further sub-typed using the Causative Classification of Stroke system as evident, probable, or possible cases of large artery atherosclerosis, cardio-aortic embolism, small artery occlusion, other causes, and undetermined causes (Ay and others, 2007). Strokes following invasive cardiovascular interventions will also be classified as such.

9.1.3 HF

9.1.3.1 HF: Defined as hospitalization, or emergency department visit requiring treatment with infusion therapy, for a clinical syndrome that presents with multiple signs and symptoms consistent with cardiac decompensation/inadequate cardiac pump function. Adjudication will use the ARIC study adjudication system (Rosamond and others, 2009). The SPRINT HF outcome will include definite or possible acute decompensation, including HF with preserved left ventricular ejection fraction as well as HF with reduced ejection fraction. HF cases may also be adjudicated as chronic stable HF but this is not considered a SPRINT outcome. In SPRINT, HF will include a variety of clinical presentations, including acute or subacute HF as the primary reason for hospital admission or for emergency department visit where HF was diagnosed and intravenous treatment was given. The identification and classification of HF cases will rely on multiple pieces of key clinical data as well as adjudicators' clinical judgment, guided by specific, pre-specified definitions and operational rules. No identification of HF should rely on a single piece of data such as the presence of dyspnea or of edema, a low ejection fraction, or an increased brain naturetic peptide (BNP) value. Adjudicators will use both the data available and clinical judgment to distinguish between "definite" and "possible" decompensated HF. "Definite" decompensated HF will be assigned when

decompensation is clearly present based on available data (satisfies criteria for decompensation). “Possible” decompensation will be assigned when decompensation is possibly but not definitively present, typically where the presence of co-morbidity could account for the acute symptoms (chronic obstructive pulmonary disease (COPD) exacerbation, for example).

For participants with advanced CKD with or without chronic dialysis, the ascertainment of HF can be particularly difficult, since the fluid overload can be purely the consequence of fluid retention by the kidney or absence of kidneys. Under these circumstances, the adjudicators will again use their best judgment, utilizing all available information.

9.1.4 CVD Death

9.1.4.1 CVD Death: SPRINT will use standard case definitions for classification of CVD death. Definite CVD events will be defined based on temporal relationship to a documented event (e.g., hospitalization for MI or for stroke), or postmortem findings of an acute CVD event. Probable coronary heart disease (CHD) death (Luepker, 2003) will be defined based on autopsy findings consistent with chronic CHD, prior history of CHD or documented symptoms consistent with CHD prior to death, and the absence of another likely cause of death. Possible fatal CHD will be adjudicated based on death certificate information consistent with an underlying CHD cause and no evidence of a non-coronary cause. Stroke deaths will be categorized based on the temporal relationship between the stroke event and death, in cases where the underlying cause of death is attributed to stroke. Proximal stroke death is a death attributed to stroke and occurring within 30 days of stroke; remote stroke death is underlying cause attributed to stroke and more than 30 days from stroke to death. Other forms of CVD death will also be adjudicated and include ruptured abdominal aortic aneurysm, and documented arrhythmia.

9.2 Secondary Outcomes

In addition to the primary outcome, SPRINT will assess additional clinical outcomes in order to more fully evaluate the relative effects of treating to a SBP goal lower than the currently recommended goal. In order to do so, data will be collected on secondary and other trial outcomes.   Main secondary outcomes are included in the analysis plan in Chapter 10.

9.2.1 Main secondary cardiovascular composite outcome: The main secondary composite outcome of SPRINT is comprised of the first occurrence of any of the components of the primary outcome and all cause mortality. A major and analogous secondary outcome of CVD-free survival, defined as survival without any of the primary or secondary CVD outcomes, will also be examined because of the significant proportion of elderly in the trial and the public health importance of the issue of CVD in that age group.   All cause mortality and components of the primary outcome will also be examined.

9.2.2 Main secondary renal outcome: The main secondary renal outcome of SPRINT will be the composite of a 50% decrease in eGFR or development of ESRD requiring chronic dialysis or kidney transplantation. This outcome applies to the CKD subgroup only.

9.2.3 Main secondary cognitive outcomes: SPRINT MIND will evaluate the incidence of all-cause dementia as adjudicated by an expert panel as the most important outcome for the MIND study. The second most important outcome is cognitive impairment among the Extensive Cognitive Assessment Battery participants will be tested with the full assessment battery (6.4.1.3 and 6.6.2). Each test score from the full assessment battery will be classified as indicating “impairment (1)” or “no impairment (0)” based on norms. A sum of impairment scores will be calculated indicating the total number of impairments. Detailed definitions of these outcomes are contained in chapter 6.

9.2.4 Additional secondary outcomes: In addition to the secondary outcomes specified in Chapter 10, other outcomes will also be examined separately and combined with other outcomes in composites (e.g., CVD-free survival defined above):

- Peripheral arterial disease, including carotid and peripheral revascularization, abdominal aortic aneurysm repair, and other objectively defined PAD events
- Coronary revascularization
- Transient Ischemic Attack (TIA): TIA in SPRINT will be defined as one or more transient episodes of the sudden onset of a focal neurological deficit, no lesion on brain imaging consistent with the deficit, and no signs or symptoms consistent with seizures, migraine, or other non-vascular causes.
- ECG diagnosed Left Ventricular Hypertrophy (LVH): ECG-diagnosed LVH will be defined primarily using the sex-specific Cornell voltage criteria. Other ECG-LVH criteria mentioned in the American Heart Association (AHA)/American College of Cardiology (ACC) statement on ECG changes associated with cardiac chamber hypertrophy (Hancock and others, 2009) will be also considered.
- Atrial fibrillation or flutter: In SPRINT, atrial fibrillation/flutter will be primarily detected from the scheduled study ECGs using Minnesota ECG classification (Minnesota code 8.3). Other sources of detection include hospital discharge ICD code (ICD-10 code 148 or ICD-9 code 427.3) and self-report.
- Other renal outcomes
    - Incident CKD, defined as a >30% decrease in eGFR and an end value of <60 ml/min/1.73M^2. This outcome applies only to the non-CKD subgroup. This decrease in eGFR requires a confirmatory value in the next available official SPRINT lab check.
    - Incident albuminuria, defined as a doubling of urinary albumin-to-creatinine (ACR) ratio from a value <10 mg/g to a value of >10 mg/g. This outcome applies to CKD and non-CKD subjects. This increase in ACR requires a confirmatory value in the next available official SPRINT lab check.

Chapter 10 – Statistical Considerations

The SPRINT Trial has a single primary objective and several key secondary objectives, some of which will be addressed within a number of subgroups whose target size has been guided by power computations. The primary objective is to determine whether the intensive BP treatment strategy will, when compared to a standard BP treatment strategy, reduce the incidence of serious cardiovascular events, defined as MI, stroke, heart failure, non-MI acute coronary syndrome or other cardiovascular death. This will be tested in all SPRINT participants.

The key secondary objectives are to determine whether the intensive BP strategy reduces the incidence of:

1) total mortality,
2) progression of CKD,
3) probable dementia,
4) cognitive impairment, and
5) white matter lesions detected by MRI.

The primary analysis of each of these objectives will be in different groups of participants. The analysis plan to address the primary and each secondary objective is described below, followed by estimates of the required sample size for each.

10.1 Analysis Plan

This section describes some of the key pre-specified analyses directed at the study's primary and key secondary objectives. Many other outcomes and measurements, such as blood pressure, adverse event experiences, health related quality of life, cost, and results of assays performed on blood and urine specimens will also be analyzed.

10.1.1 Analysis of the Primary Outcome in all Randomized Participants

The primary analysis will apply Cox proportional hazards regression (Cox, 1972) to all randomized participants to compare the time from randomization to the first occurrence of the primary CVD composite endpoint between the randomized BP groups. The model will include an indicator for intervention arm as its sole predictor variable. Clinical site at randomization will be a stratifying factor. Follow-up time will be censored at the last date of event ascertainment. The p-value from the primary analysis will be based on the chi-square statistic from a likelihood ratio test obtained from proportional hazards models with and without the term for intervention arm. This likelihood ratio test will constitute the primary test of statistical significance for the primary analysis.

Primary comparisons of intervention groups will be performed according to the intention-to-treat principle. All randomized participants in these analyses will be grouped according to their intervention assignment at randomization, regardless of adherence.

10.1.2 Secondary analyses supporting the primary analysis

10.1.2.1 Secondary outcomes. A number of secondary outcomes will be analyzed to clarify the interpretation of the results of the primary analysis. These will include:

a) all myocardial infarction,
b) all stroke,

c) non-MI acute coronary syndrome,
d) all heart failure,
e) CVD mortality,
f) total mortality, and
g) a composite of total mortality and the primary composite outcome (i.e. major CVD event- free survival).

Each of these will be analyzed using a proportional hazards model as described for the primary analysis. These will be reported with 95% confidence intervals and nominal p-values without an adjustment for multiple comparisons, since the intent is to articulate a pattern of effects closely related to the primary outcome, rather than to provide additional tests of efficacy.

10.1.2.2 Subgroup analyses. In addition to the analysis of the secondary outcomes described above, a set of analyses will be reported to explore whether intervention effects on the primary and confirmatory secondary outcomes are consistent across subgroups of interest. These subgroups are:

a) CKD (defined as eGFR < 60 at randomization) vs. non-CKD,
b) senior vs. non-senior (aged ≥75 at randomization vs. aged <75),
c) male vs. female,
d) black vs. non-black,
e) with and without a history of CVD at randomization (as defined in Chapter 3), and
f) tertiles of systolic blood pressure at baseline.

The subgroups defined by CKD, age and race are motivated by biologically plausible hypotheses. For each subgroup analysis, a proportional hazards model will be used that is similar to the one described for the primary analysis above, but with additional terms identifying subgroup membership and the intervention by subgroup interaction. The nominal p-value for the interaction term using a likelihood ratio test will be reported along with within subgroup estimates of the intervention effect and associated nominal 95% confidence intervals. We will report the Hommel adjusted p-values for the interaction effects.

10.1.3 Non-cardiovascular clinical outcomes

10.1.3.1. Acute vs. chronic effects of intervention

It is possible that the intervention will have some acute adverse effects due to under-perfusion of various organs, notably the kidney and the brain, which are major targets of SPRINT. In the long term, however, lower SBP may protect these organs from hypertension-related damage. We will examine the possibility of acute effects as part of the data monitoring plan, particularly if differential adverse effects are observed early in the trial; we also will examine the possibility of acute effects as part of the data analysis at the end of the trial.

10.1.3.2 Renal outcomes

Renal outcomes are of particular importance in SPRINT, both to assess the incidence of new kidney disease among participants free of CKD at baseline and to assess the progression of kidney disease among those with CKD at baseline. Because some

outcomes are more interpretable in either people with CKD or without CKD at baseline, some analyses will be restricted to these subgroups.

The primary hypothesis for the renal outcomes is whether, in the subgroup with CKD at baseline, the rate of a composite of a 50% decrease in eGFR or ESRD undergoing chronic dialysis or kidney transplantation is lower in the intensive intervention arm. The decline in eGFR must be seen on two visits at least three months apart. This will be analyzed using a proportional hazards model as described for the primary CV analysis.

A number of additional analyses related to this hypothesis will also be performed. These will include:

a) incident CKD in the non-CKD subgroup, defined as a 30% decline from baseline eGFR to a value of <60 mL/min/1.73m^2 (observed on two visits at least 3 months apart. There must be a decrease of at least 30% AND the end value of this decrease must be <60 ml/min/1.73m^2 in order to satisfy this endpoint criterion) or ESRD
b) incident albuminuria, defined as a doubling of urinary albumin-to-creatinine (ACR) ratio from a value <10 mg/g to a value of >10 mg/g. This outcome applies to CKD and non-CKD subjects. This increase in ACR must be observed at two visits at least 3 months apart.

Subgroup analyses. Analyses of the renal outcomes will be by CKD and non-CKD strata. Within each strata, assessments of the renal composite endpoint will be by subgroups. The analytical approach will be the same as for the primary CV analysis as described in 10.1.2.2. The renal subgroups are:

a) urinary albumin/creatinine ratio (>300 mg/g and ≤ 300 mg/g),
b) black vs. non-black,
c) senior vs. non-senior (aged 75+ at randomization vs. aged <75),
d) male vs female,
e) eGFR (median split)

The subgroups defined by albumin/creatinine ratio, age and race are motivated by biologically plausible hypotheses. The main renal outcome composite is defined differently for the CKD and non-CKD strata, so that these will be separate analyses.

10.1.3.3 Dementia and cognitive outcomes.

The primary outcome for SPRINT MIND will be the first identification of adjudicated dementia. Cox proportional hazards models (as described above for the SPRINT primary outcome) will be used to compare the time from randomization to the first identification of dementia between the two treatment arms. All participants will be screened for dementia at baseline.

Secondary analyses. Secondary analyses in the areas of cognitive function, small vessel ischemic disease (SVID) lesion load, and mild cognitive impairment will also be performed to support the primary analysis.

Cognitive Function. A cognitive assessment battery will be administered at baseline and 2 and 4 years post-randomization in a subsample of 2800. The primary outcomes will be composite scores for two domains: 1) Memory, consisting of the Hopkins Verbal Learning Test, Logical Memory and the Modified Rey Osterrieth Figure, and 2)

Processing Speed, consisting of Trails Making Tests and Digit Symbol Coding Test. Changes in impairment over time will be compared between the two treatment arms.

Supporting analyses will also be conducted on the effect of the interventions on individual domains of memory over 48 months. Follow-up test scores will be compared using mixed-effects analysis of covariance models (Laird, 1982). Mixed-effects models allow for departure from linearity in the relationship between the outcome and time. Estimates of the difference in mean levels of the outcome between control and intervention groups will be obtained using maximum likelihood techniques. Sensitivity of results to missing data will be investigated through the use of multiple imputation techniques (Rubin, 1987).

Magnetic Resonance Imaging (MRI). Other than age, hypertension is the strongest correlate of SVID. Total SVID lesion load including abnormal white matter, abnormal gray matter and abnormal basal ganglia will be the SPRINT measure of total SVID lesion load. Differences in total SVID lesion between treatment groups at 48 months will be the main outcomes of the MRI component. Furthermore, differences in total brain volume will also be compared after 48 months. These measures are continuous and will be analyzed using mixed effects analysis of covariance models as described above.

Mild Cognitive Impairment (MCI). This outcome is defined as the time to the first of two consecutive occurrences of MCI. Analytical methods used for dementia will be applied to the analyses of MCI, in those free of MCI at baseline. Furthermore, these same methods will be applied to the analyses of the first cognitive impairment defined as the first event classified either as MCI or dementia in those free of MCI at baseline.

Subgroups. Analyses of the cognitive outcomes will also explore the intervention effects within subgroups. The analytic approach will be the same as for the primary CV analysis as described in 10.1.2.2. The subgroups are:

a) CKD (defined as eGFR < 60 at randomization) vs. non-CKD,
b) senior vs. non-senior (aged 75+ at randomization vs. aged <75),
c) male vs. female,
d) black vs. non-black,
e) with and without a history of CVD at randomization (as defined in Chapter 3),
f) tertiles of systolic blood pressure at baseline,
g) MCI at baseline (yes vs. no),
h) orthostatic hypotension (yes vs. no).

The subgroups of CKD, age, and MCI are motivated by biologically plausible hypotheses.

10.1.4 Other analyses

We expect to explore fully the rich set of data that SPRINT will obtain. Exploratory analyses of biologically plausible subgroups are of particular interest. Some of these will be further articulation of supporting subgroup analyses described above, such as analysis of continuous baseline factors as continuous variables rather as pre-specified categorical variables. Other analyses will involve baseline variables that are not listed in the pre-specified subgroup but which may modify treatment effect, such as diastolic blood pressure or presence of the metabolic syndrome.

10.1.5 Missing data

Consistent with an intention-to-treat analysis, we will categorize all participants by their randomization group, regardless of compliance, in our primary analyses. For those participants lost to follow-up, we plan to use all available information until the time of death or loss to follow-up.

Our approach to handling missing outcomes in clinical trials is consistent with the opinion of Molenberghs and Kenward (2007, p9), who state that while ignorable, missing-at-random (MAR) analyses are reasonable for the primary analysis, exploration of the sensitivity of conclusion to the MAR assumption may include models which allow for missingness that is not random. If loss to follow-up is related to the level of the outcome being analyzed (e.g. as often occurs when analyzing health related outcomes), then results obtained under the assumption of independent loss to follow-up may be biased. The magnitude of this problem will be investigated by using measurements taken at previous visits to predict loss to follow-up. Variables determined to predict loss to follow-up will be included in our predictive models in order to satisfy the conditions described by Little and Rubin (1987) for the data to be considered MAR. Maximum likelihood techniques will be used to estimate parameters. If necessary, other approaches may be examined in consideration of how robust the results will be and whether they provide appropriately conservative estimates for the trial.

In order to explore the possibility of a relationship between ESRD and CV outcomes, we will conduct sensitivity analyses which treat ESRD as a censoring point for the primary outcome. This exploration may include an auxiliary composite outcome combining the events in the primary outcome and ESRD.

Robustness of inferences to missing outcome data will be further explored in sensitivity analyses. These analyses will include examination of several "worst-case" scenarios, including opposite and pooled imputation approaches (Wittes, Lakatos & Probstfield 1989; Proschan et al., 2001). These types of scenarios are members of a broad class that can be parameterized as pattern mixture models (Little 1993) and allow for examination of sensitivity of conclusions to missing-not-at-random (MNAR) mechanisms (Mohlenberg and Kenward, 2007).

The MRI substudy involves two assessments—one at baseline and one at 48 months—in 640 participants, thus limiting the range of analytic strategies. We recommend using maximum likelihood based general linear models for analyzing outcomes. Intracranial volume will be included as a covariate. The validity of the MAR assumption can be improved by including baseline covariates that predict missingness. If loss to follow-up is related to the unobserved cognitive outcome then our results may be biased. Again, some modeling and sensitivity analysis options may be considered if necessary.

10.2 Sample Size Estimation and Power Calculations

10.2.1 Primary Outcome

We have assumed a 2.2 %/yr event rate of the primary outcome in the standard group, a 20% effect size for the intervention (hazard ratio of 0.8), a two-year uniform recruitment period, a total study length of 5 years and 10 months, a 2 %/yr rate of loss to follow-up,

and a two-sided test at the 5% level. With these assumptions, power for a variety of sample sizes is presented in Table 1. Power is also presented for hazard ratios of 0.78 and 0.82 and for event rates of 2.0 and 2.4 %/yr. A sample size of 9250 provides high power for a hazard ratio of 0.8 (representing a 20% effect) and a 2.2 %/yr event rate. This sample size would also provide over 80% power for an effect of 18% (hazard ratio of 0.82) with an event rate of 2.2 %/yr and would have reasonable power of 77.3% even with a smaller than assumed event rate of 2.0 %/yr and an 18% effect. Depending on the observed event rate and treatment effect, the table below shows that sample sizes of 8500 to 10000 would be consistent with study goals.

Table 1: Power for the SPRINT primary outcome.

	Event Rate								
	2.0 %/yr			2.2 %/yr			2.4 %/yr		
N\Hazard Ratio	0.78	0.8	0.82	0.78	0.8	0.82	0.78	0.8	0.82
8500	89.4	82.7	73.7	91.9	85.9	77.6	93.9	88.6	80.9
8750	90.3	83.7	75.0	92.6	86.9	78.7	94.5	89.5	82.0
9000	91.0	84.7	76.1	93.3	87.8	79.8	95.0	90.3	83.0
**9250**	**91.7**	**85.7**	**77.3**	**93.9**	**88.7**	**80.9**	**95.5**	**91.0**	**84.0**
9500	92.4	86.6	78.3	94.4	89.4	81.9	95.9	91.7	85.0
9750	93.0	87.4	79.4	94.9	90.2	82.9	96.4	92.4	85.9
10000	93.6	88.2	80.4	95.4	90.9	83.8	96.7	93.0	86.7

If the event rate in the standard therapy arm is substantially less than 2.2%, we may ask that the DSMB consider recommending a two year extension of the trial.

10.2.2 Summary

For the primary outcome under the assumptions detailed below, with 9250 participants, the SPRINT study is designed to have

- 88.7% power to detect a treatment effect of 20% of intensive blood pressure control compared with standard blood pressure control,
- 81.9% power to detect a treatment effect of 20% of intensive blood pressure control compared with standard blood pressure control among participants with estimated glomerular filtration rates of <60 ml/min/1.73m^2 at baseline,
- 84.5% power to detect a treatment effect of 25% of intensive blood pressure control compared with standard blood pressure control among participants at least 75 years old at baseline,
- 96% power to detect a 20% effect and 80% power to detect a 15% effect for incident dementia, the primary outcome for SPRINT MIND.

These estimates of power are valid under the following assumptions:

- The primary outcome for SPRINT is a composite of fatal CVD, MI, stroke, heart failure, and non-MI acute coronary syndrome.
- The event rate for this composite outcome is
  - 2.2 %/yr in the standard BP arm,
  - 4 %/yr among participants with eGFR <60 ml/min/1.73m^2, and
  - 3.5 %/yr among participants ≥75 years old.
- The event rate for the SPRINT MIND primary outcome of incident dementia is 3.1%/yr.
- There are

  - 9250 participants in SPRINT,
  - 4300 participants with eGFR < 60 ml/min/1.73m^2, and
  - 3250 participants ≥75 years old.
- Participants are recruited uniformly over 2 years.
- Minimum follow-up is 3 years, 10 months which assumes that closeout visits occur uniformly over a 4 month period.
- Two-sided tests at the 0.05 level are used.
- Annual loss to follow-up is 2 %/yr (3 %/yr for incident dementia).

Additional computational details and a justification for the assumed event rates are included in the appendix.

10.2.3 Power for the MIND primary outcome

Power for the MIND primary outcome is presented in Table 10.2 for a range of event rates with 9250 participants, 5 years and 10 months of follow-up, 2 years of recruitment, and 3 %/yr loss to follow-up. Details of the event rate estimation are given in Appendix 3.

Table 10.2. Power for the SPRINT MIND primary outcomes.					
	Event Rate (%/yr)				
Hazard Ratio	3.1	3.2	3.3	3.4	3.5
0.80	96.3	96.7	97.1	97.4	97.7
0.85	79.0	80.2	81.3	82.4	83.4

10.3 Statistical Reports

10.3.1 Steering Committee Reports

Periodic reports will be generated for the Steering Committee, CCNs and Clinical Sites. These reports will include information on recruitment, loss to follow-up, adherence, baseline covariate information on the comparability of treatment groups, and adverse events. Information will be stratified by CCNs and Clinical Sites. Other reports will include information on quality control for central facilities and data entry.

10.3.2 Data and Safety Monitoring Board Reports

The role and composition of the Data and Safety Monitoring Board are described elsewhere (Chapter 13.6). Meetings of the DSMB will be held at least annually. Material for these meetings will be distributed two weeks in advance of the meetings. Up-to-date statistical analyses will be provided to the DSMB in preparation for their meetings. The analyses will include data on recruitment, outcome measures, any side-effects or safety concerns, adherence, and quality control, and will be designed in cooperation with the DSMB. Interim analyses of the intervention effectiveness will be performed at times coinciding with the meetings of the DSMB, and will be controlled to protect the overall Type I error of the trial. These results will be for the use of the DSMB and will not be revealed to the investigators. The purpose of these analyses will be for the DSMB to assess the trial progress with respect to intervention efficacy and safety, for possible recommendations regarding early termination of the trial.

We will work with the DSMB to finalize the monitoring plan. We include here a potential starting point for those discussions.
Interim analyses will be performed periodically for the DSMB. Monitored parameters will include the following:

1. SBP separation between groups
2. SBP distribution within groups
3. Primary outcome results
4. Adverse events
5. Laboratory alerts
6. Recruitment progress
7. Other event rates, and event rates by subgroups
8. Enrollment overall and by subgroups such as level of eGFR and CKD category

Sequential monitoring and early stopping. Incidence rates of outcomes will be monitored throughout the trial and used for interim analyses of efficacy and futility. Group sequential methods for event rates will be used to control the Type I error to be 0.05 across these repeated analyses. Critical values for interim testing will be defined based on an O'Brien-Fleming type bound and will use a spending function to allow flexibility in the number and timing of interim analyses. With this approach, interim tests early in the trial are conservative and the reduction in the overall power of the trial caused by interim testing is small. If needed, conditional power calculations will be used to assess the futility of continuation in the presence of a negative treatment effect.

The monitoring plan will include consideration of the hypothesis that early adverse effects may occur and then be followed by long-term beneficial effects. Because kidney function will be measured at baseline, 1, 3, and 6 months, we will be able to analyze the acute impact of our intervention on kidney function. Because of the study design, episodes of acute kidney injury (AKI) that are of more than a transient nature will be identified as changes in chronic kidney function, consistent with contemporary paradigms acknowledging the interrelationships between AKI and CKD. Episodes of AKI will be specifically sought in review of medical records in appropriate patients as adverse events. Regarding the possibility of acute cognitive decline, spontaneously reported SAEs would be the source of such information.

At each meeting, the DSMB will review data on adverse events and other safety issues to make an overall recommendation to the NIH concerning the safety of continuing SPRINT. Consistent with NIH policy, each SPRINT CCN Principal Investigator will receive a report summarizing the DSMB review of the adverse event data. Principal Investigators are responsible for providing this report to their sites and institutional IRB.

10.3.3 Website Reports

The Coordinating Center will prepare many reports and place them on the SPRINT website. These reports enable a user to click on a static link which starts a real-time report processed by SAS and returned as output in the user's web browser. These reports access live data and run within seconds. Examples of real-time reports on randomization and screening activities include: number of clinics actively recruiting, percent at target (overall, to date, and by demographic subgroups such as women and race/ethnic group). Clinical Sites will have access to live data showing exactly where their clinic stands in relation to their recruitment goals and those of the other Clinical Sites, as well as projections of activity needed to meet their goals. Committee members

will have expanded access to information across all Clinical Sites for the purpose of monitoring recruitment performance for the trial as a whole.

Chapter 11 – Data Management

11.1 Overview: Use of the World Wide Web

All Clinical Center Networks and Clinical Sites will use the World Wide Web (WWW) to enter SPRINT data collected on forms from participants seen within the Clinical Sites. Each Clinical Site will have a password protected area on the SPRINT home page through which data will be entered. Documentation of the data entry system will be maintained at the CC. In addition, training materials for measurement and data entry personnel will be available in downloadable format on the SPRINT web site. Site-specific reports relating to participant demographics, recruitment goals, etc., among other reports, will be available on the web site.

Data security in the web-based data system uses 128-bit encryption and Secure Socket Layer (SSL). Once data has been received at the CC, recovery from disasters such as natural phenomenon (water, fire, or electrical) is possible through the ability to reconstruct both the database management system and the data up to the last back-up through the use of nightly backups. This will ensure optimal recovery of data systems in the event of a disaster. Back-up tapes are kept in a locked, fire and waterproof storage cabinet away from the computer room. Additional back-up tapes will be stored at another location on the Wake Forest University Health Sciences campus. CCNs and clinical sites have local procedures for back-up and recovery of data following a disaster. As a supplement to those plans, the SPRINT CC will have all participant contact information to minimize the chance for disruption of communication with participants regarding study medications and test results.

11.2 Flow of Data from Trial Units to Databases

11.2.1 Data from Clinical Sites and Clinical Center Networks

Participant Randomization: SPRINT will use an internet-based, web browser randomization procedure. Clinical Sites access the randomization application through the study web site. Access to this application is password protected and its communications are encrypted. Once security requirements are satisfied, a series of questions identify and verify the eligibility of the participant. When the session is complete, an e-mail is sent to the Clinic Coordinator, the appropriate CCN, and the CC indicating that the participant has been properly randomized and appended to the database.

Participant Tracking: The Participant Tracking System (PTS) is a fully integrated tracking and notification system that advises clinic staff about participant follow-up windows, and projects clinic and laboratory workload for a week at a time (longer if necessary). Tracking a participant begins at screening and continues automatically throughout the project by integrating participant follow-up data with predetermined follow-up "windows". When a participant is enrolled into the study, a schedule of target dates for each of the visits is automatically generated. The report details the recommended "windows" that each visit should fall into and a case file is created for the participant.

Data Entry: The images on the data entry screens mirror the data collection forms for ease and accuracy of entry. Typically, as participant visits are completed, and hard copy

forms are filled out, the clinic coordinator reviews each form for accuracy and completeness, including laboratory reports and any supporting documentation (hospital records, etc.). Once any data problems have been resolved, data are entered by clinic staff into the computer via the web-based browser application. During data entry, a variety of programmed edit checks are performed for key variables. When the edit checks fail, data may be flagged for further review or prevented from becoming part of the study database. Also, a sample of key forms may be double-keyed for additional quality control.

11.2.2 Data from Central Laboratory and ECG Reading Center

Laboratory specimens and electrocardiographic data are sent to the Central Laboratory and ECG Reading Center from the Clinical Sites on a fixed schedule. The Central Laboratory and ECG Reading Center provide results to the CC on live internet feed. Depending on clinic needs, reports will be sent to assist in the clinical functions (e.g., providing timely feedback to the clinic on any measurement that exceeds a predefined alert level).

11.2.3 Central Database Edits

At regular intervals, data queries will be carried out on the computerized databases at the CC to perform consistency checks on key variables and forms. Although much of this will have been done at the data entry level in the clinic, this additional pass through the data serves as a quality control check.

11.3 Feedback to Clinical Sites and Clinical Center Networks

Data edit reports will be generated to help ensure that data are entered in timely and complete manner. These reports will include both the assessment for each Clinical Site of the time between data collection and entry, and the generation of reports by the CC of missing items. These reports will be provided to the Clinical Center Networks, Clinical Sites, and study committees on a regular basis so that data collection items that are troublesome can be identified and Clinical Sites not meeting study standards can be notified. CCN Coordinators will be copied on all data reports for Clinical Sites within their network and asked to follow-up on any action that needs to be taken.

11.4 Confidentiality

The confidentiality of all participant information (including but not limited to any genetic analysis) must be protected at the Clinical Sites, the CCNs, and the CC. Paper records and computer files must be appropriately safeguarded from unauthorized access.

Paper and/or electronic records for study participants will be stored at the Clinical Sites. Copies of records identified by participant identification number pertaining to SAEs and study-defined clinical events, including necessary medical records, will be stored at the CC. These records will receive the same care as would ordinary medical records. They will be stored in locked filing cabinets and/or filing rooms within secure office space. Only study personnel who have completed SPRINT training in data handling will have access to study forms.

Similar care will be used in the handling of the computer records of study data stored at each Clinical Site. Access to the data in any local SPRINT database will be controlled by a system of user identification names and passwords. Each Clinical Site staff member must complete the SPRINT data handling training program before being given an ID and password to use the data system. The privileges allowed to each ID can be individually specified by the local CCN Coordinator. All passwords stored within the system will be encrypted using SSL encryption.

Confidentiality of information within the CC will be protected through a variety of procedures and facilities:

1. The confidential nature of the data collected, processed, and stored at the CC is explained to all new personnel.

2. All access to CC office space containing data is controlled through a single door, which is locked with a keypunch lock. This door remains locked at all times.

3. All participant data sent to the CC is encrypted as described above.

4. All participant data stored on the Wake Forest University's mainframe computers are likewise encrypted. In addition, all such databases are protected by passwords that must be supplied before the data can be accessed. Passwords are released only to CC staff with a need to use the particular file, and are changed on a regular schedule.

5. All printouts, plots, and reports containing individually identifiable data are produced on printers and plotters within the CC's secure office space.

6. The CC will obtain a Certificate of Confidentiality for SPRINT, which prevents researchers from being forced to disclose identifying information by certain legal proceedings.

SPRINT Protocol
Chapter 12 – Quality Control

12.1 Introduction

Data integrity and quality are among the highest priorities in SPRINT. This feature is reflected in the details provided in the protocol regarding initial screening and recruitment of participants, data acquisition at baseline and follow-up visits, outcome definition and assessments, reading and/or interpretation of the results, and their analysis and publication. There are two primary purposes for quality control: to document the level of quality and to provide feedback to the clinical, reading and laboratory centers in order to maintain and improve the quality of the study data over the course of the trial. The Measurement Procedures and Quality Control Committee will establish guidelines for quality assurance and quality control, detailed in the Manual of Procedures.

Quality control monitoring in SPRINT will involve the CC, the CCN hubs, and various SPRINT committees and other groups, although the Measurement Procedures and Quality Control Subcommittee will monitor quality control and quality assurance activities for the study overall, integrating input from these other groups. For example, the Recruitment, Retention and Adherence Subcommittee will monitor progress toward achieving recruitment goals, and the SPRINT MIND subcommittee will monitor the quality of assessment with the cognitive battery. The CC will generate reports and supply them to the CCN hubs for their sites, to the Measurement Procedures and Quality Control Subcommittee for all sites and entities, and to other involved groups for the activities in their purview. The CCN hubs will be responsible for tracking the performance of sites within their Networks, and for following up with their sites on areas of concern. The Measurement Procedures and Quality Control Subcommittee will conduct monitoring for the trial overall, will raise issues on specific sites and communicate them to the CCN hub for follow-up, will monitor the central facilities (ECG reading center and central lab), and will report any areas of concern to the Steering Committee for consideration, as needed.

This chapter outlines the type of quality assurance activities that will be conducted in the SPRINT Trial. Two phrases are used. The first, quality assurance, is the collection of manuals and procedures that will be in place to assure the integrity of the data. A subset of these procedures is referred to as quality control, which describes the monitoring and analytic activities that assess performance during data collection and its processing.

12.2 Manual of Procedures

As with any multicenter study, standardization of study procedures is very important in the SPRINT Trial. The MOP includes the detailed descriptions of all trial procedures. This MOP is used for training purposes and as a reference for all study investigators and staff. The MOP is an important aspect of efforts to standardize study procedures across clinical sites in the SPRINT Trial.

Key study procedures will be standardized; these include the use of a central lab and ECG reading center, and standard forms, equipment, and procedures in the clinics for

BP measurement and other data collection procedures. Furthermore, standard event definitions and event validation procedures will be used.

12.3 Study Forms and Data Entry Procedures

Quality assurance concepts were employed during the development of forms. Forms are printed with accompanying question-by-question instructions for easy reference. Web-based data entry screens will be developed from the forms, and enable the incorporation of range and logical checks at the time of data entry. These features will contribute to quality assurance.

12.4 Training

Training of staff and pilot testing of procedures will be crucial to standardize procedures and assure data quality. SPRINT uses two different training models: central training for study staff and the train-the-trainer approach. In the central training aspects of the SPRINT training effort, all relevant staff members from all clinical sites will be convened in a single, centrally administered face-to-face training session. This approach is cost-efficient and contributes to uniformity of the training experience and thereby to uniformity of data quality across sites. In the train-the-trainer aspect of the SPRINT training effort, CCN hub staff will provide training sessions to persons who were unable to attend the central training session and to newly hired staff as turnover occurs. In addition, the CCN hubs will organize training and refresher training sessions, as needed, including CCN remedial training in specific areas targeted by quality control monitoring for a specific site.

12.5 Data Queries

The Coordinating Center will be responsible for data editing, which will include checks for missing data, unrealistic values, and crosschecks for inconsistencies. Data will be checked on form submission and any data queries presented to the data entry staff for immediate resolution, if possible. The CC will also produce data query reports on the website that summarize the number and types of queries by clinic and network. Clinical center staff will be responsible for reviewing and resolving the data queries in a timely manner. Reports, including reports on timeliness of data entry and query resolution, will be shared with the Measurement Procedures and Quality Control Subcommittee and the corresponding CCN hub investigators and staff for quality control purposes.

12.6 Quality Control Reports

The Measurement Procedures and Quality Control Subcommittee will develop quality indicators, both to document data quality and to provide feedback to individual clinical sites, that will be tracked in routine quality control reports in the SPRINT Trial. All reports will be generated by the CC and distributed to the Subcommittee, to the corresponding CCN hub, and/or to other relevant groups (e.g., the SPRINT MIND subcommittee for those measures). Investigators and staff at the CCN hubs will be responsible for disseminating reports and feedback to the appropriate investigators and staff at the clinics in their networks. These reports will be used to inform discussions that will take

place during regularly scheduled telephone contacts and site visits. Additional information about these processes is contained in the MOP.

Quality Control reports will focus on measures of process, impact, and outcomes. Examples of process measures that will be tracked for quality control purposes include:

1. Days between data collection and data entry
2. Percent of forms with late data entry
3. Number of participants with missed or late visits by contact, number of missed or late visits clinic-wide, and number of participants missing two or more consecutive visits
4. Number, name and dose of prescribed antihypertensive medications for individual participants

Examples of impact measures that will be tracked for quality control purposes include:

1. Number (and percent) of participants at goal according to the BP target assignment as assessed by in-clinic BP measurements.

Examples of outcome measures that will be tracked for quality control purposes include:

1. Submission of medical record documentation for reported study events by the clinical site (e.g., timeliness, completeness)
2. Proportion of participants with ECG submitted to central ECG Reading Center overall and by quality grade
3. Proportion of participants with urine samples submitted for albuminuria assessment
4. Proportion of participants with blood samples submitted to central lab
5. Percent agreement of individual study adjudicators with the final outcome assignments for cases adjudicated

Details of the various quality control procedures are contained in the Manual of Procedures. In general, the CC will generate reports and analyses on progress at the clinical sites on an agreed upon schedule appropriate to the study phase. Reports will most often be developed at the level of the clinical site but may also include patient-level reports by site, technician-level reports by site, and summary reports study-wide and within and across CCNs. The CC will supply these reports to the Measurement Procedures and Quality Control Subcommittee, to other relevant Subcommittees, and to the corresponding CCN hub investigators and staff.

12.6.1 Deviations from protocol

Adherence to the study protocol is crucial to collection of high quality data and to the internal validity of the trial. Thus, the Intervention Subcommittee will define important deviations from the intervention protocol for tracking purposes. A clinic-site-specific report describing important protocol deviations will be disseminated by the CC to the respective CCNs for quality control purposes. Copies of these reports and a summary report describing important protocol deviations and plan for corrective actions on a study-wide basis will be shared with the Measurement Procedures and Quality Control Subcommittee and the Steering Committee.

12.6.2 Monitoring the Clinical Centers in the Networks

Primary responsibility for clinical site monitoring in SPRINT will be assigned to the corresponding CCN hub. CCN hub investigators and staff will be responsible for monitoring performance at each of their clinical sites. The CCN hub monitoring team will coordinate research activities of the study within their network and maintain effective communications with clinical sites, other clinical center networks, the coordinating center, project office and study central units (Central Lab, ECG Reading center, MRI Reading Center and Drug Distribution Center). One of the primary roles of CCN hubs is to monitor clinical sites in all aspects of trial operations and performance and to assist in problem solving related to all aspects of the main study and ancillary studies. Site monitoring can and will be performed using regular communications including email, conference calls, site visits and other means.

12.7 Site Initiation

Clinical site initiation to enroll and randomize participants is dependent upon completion of a series of preliminary tasks. These include completion of appropriate regulatory approvals (IRBs), and letters of agreement. Site staff training, certification, and receipt of all study supplies including medications will need to be completed as well as the development of a recruitment plan. CCNs will provide the appropriate assistance to their clinical sites toward these ends, which may include site visits to ensure that the study enrollment and randomization process follows proper study procedures.

12.8 Site Visits

12.8.1 CCNs to clinical sites

During the course of the trial, clinical center network personnel will site visit clinical sites in their network at specified intervals, and as needed. The scope of these visits is broad and can include but is not limited to regulatory requirements, study communications, site initiation, site staffing, and general site performance. A minimum standard for all site visits content and frequency is detailed in the MOP; however, areas of emphasis and/or additional monitoring may vary according to the circumstances of a specific site and site visit. Site visits may be conducted to evaluate performance deficits in one or more critical areas, such as consistent departures from the protocol or MOP. Site visits are also an opportunity for refresher training and/or training of new staff, as needed. Site visit frequency and visit procedures can be found in more detail within the appropriate section of the MOP.

Site visitors will include CCN hub and site staff and investigators as deemed appropriate. As needed, representatives from the coordinating center, project office, other CCNs, and study committees may attend these visits.

A summary of the site visit will be presented to the clinical site investigator and staff at the conclusion of the site visit. The CCN staff will prepare a written site visit report within a reasonable time-frame post visit. Copies of the site visit report will be sent to the clinical site investigator, the coordinating center, the project office, and the CCN. Additional copies of the site visit report may be requested by other SPRINT Study entities.

A sample of site visit reports may be reviewed by the Measurement Procedures and Quality Control Committee or other study committees with recommendations for follow-up actions and/or reporting changes as needed.

12.8.2 Coordinating Center to CCN hubs

The SPRINT Coordinating Center will periodically site visit each CCN hub in order to monitor and ensure high performance throughout the trial. Representatives from the NIH SPRINT project office (including NHLBI, NIA, NIDDK, and NINDS) and study leadership may also attend.

12.8.3 Project Office to Coordinating Center

Representatives from the NIH SPRINT project office and study leadership will visit the coordinating center in order to monitor and ensure high performance throughout the trial.

12.9 Laboratory and ECG Center Quality Control

The SPRINT Measurement Procedures and Quality Control Subcommittee will work with the Coordinating Center, the Central Laboratory and the ECG Reading Center to develop quality control procedures to ensure high quality data, including monitoring clinical site performance as well as performance of the Central Laboratory and ECG Reading Center. The results of quality control procedures performed at the Central Laboratory and the ECG Reading Center will be reported on a regular basis to the Measurement Procedures and Quality Control Subcommittee and by them to the Steering Committee.

<u>Core Laboratory for Blood and Urine Assays</u>
Clinical site performance in acquisition, handling, storage and shipping of specimens will be tracked by the Central Laboratory and the Measurement Procedures and Quality Control Subcommittee. The first step in quality assurance at the site level consists of the training and certification process for staff within the clinical sites. Other steps include maintaining logs of equipment checks at each clinical site according to the Manual of Operations; observation of technicians performing all steps of sample collection and processing during site visits; reviewing study forms, documentation of staff certification/re-certification, and other records to ensure only certified staff are obtaining and processing specimens; reviewing and tracking the condition of samples received at the Central Laboratory for problems in shipment; and periodic analysis of the study data for participant compliance with fasting, where required, and for signs of problems in drawing or processing, such as hemolysis. Reports on clinical center performance will be submitted regularly by the Central Laboratory to the CCN hubs and the SPRINT Measurement Procedures and Quality Control Subcommittee.

Performance of the Central Laboratory will be monitored regularly by the SPRINT Measurement Procedures and Quality Control Subcommittee. Quality Control procedures in the laboratory for assays include the use of the internal Laboratory Manual, training and certification of Laboratory staff, Laboratory participation in external standardization and certification quality control programs, and implementation of the SPRINT internal quality control program. Process measures, such as turn-around time for the Laboratory reporting back relevant analyte results to the clinical sites, will also be

monitored. Particular attention will be paid to the feed-back of pre-specified laboratory alerts to the Clinical Sites by the Central Laboratories.

As part of the internal quality control program specified in the manual of operations, the Central Laboratory will regularly provide summaries of the internal quality control results to the Coordinating Center, including the following information for each assay: (1) monthly summary statistics (n, mean, and standard deviation) on all quality control pools, including new pools being overlapped to replace established QC pools; (2) summaries of any unusual problems or conditions noted. The SPRINT Measurement Procedures and Quality Control Subcommittee will review these reports for evidence of trends with time in results on these pools.

ECG

Clinical site performance in acquisition and submission of ECG tracings will be tracked by the Reading Center and by the Measurement Procedures and Quality Control Subcommittee. The first step in quality assurance at the site level consists of the training and certification process. All SPRINT staff acquiring ECGs must be certified, consisting of the successful recording and transmission to EPICARE of three successive, adequate quality ECGs. The ECG Reading Center will continuously monitor ECG quality and will identify errors in acquisition. Each tracing submitted will be graded for quality and used to compile continuous quality trend analysis data for each clinical site. Quality control grade reports will be regularly submitted to the CCN hubs and to the SPRINT Measurement Procedures and Quality Control Subcommittee.

The ECG Reading Center has an internal quality control protocol that monitors performance of ECG coding and measurement. This includes regular monitoring of the repeatability and accuracy of editing ECG waveforms of the digital (electronic) ECGs, and procedures to safeguard against change in trends due to change in ECG reading software. The SPRINT Measurement Procedures and Quality Control Subcommittee will monitor performance of ECG coding and measurement within the ECG Reading Center by regularly reviewing the results of the center's quality control reports.

Chapter 13 – Study Organization

13.1 Overview

The SPRINT organizational structures and responsibilities are similar to those of other large multicenter clinical trials sponsored by government or industry. The National Heart, Lung, and Blood Institute (NHLBI) initiated this study, and the National Institute of Diabetes and Digestive and Kidney Diseases (NIDDK) is a co-sponsor of the main SPRINT trial. The National Institute of Neurological Disorders and Stroke (NINDS) and the National Institute on Aging (NIA) are jointly sponsoring the SPRINT MIND study. Five Clinical Center Networks and a Coordinating Center work together through the Steering Committee to successfully design and conduct the trial (see Figure 13.1). In addition, there is a Central Laboratory, an ECG Reading Center, an MRI Reading Center and a Drug Distribution Center. Scientific leadership is provided by the Steering Committee. External oversight is provided by Institutional Review Boards and a Data and Safety Monitoring Board.

13.2 Clinical Center Networks and Clinical Sites

SPRINT participants will be recruited, randomized, treated, and followed through a system of five CCNs. Each CCN consists of collaborating clinical sites, which are medical facilities and/or individual practices involved in the initial evaluation, enrollment, treatment and follow-up of participants in the trial. Each CCN and clinical site will be responsible for timely recruitment and protocol adherence in accordance with the SPRINT protocol and MOP. In addition, the CCNs will contribute to the study's scientific leadership and operational management, and each CCN Principal Investigator (PI) will participate in Steering Committee and other investigator meetings. The clinical sites will collect data at the local level in accordance with the study protocol and the manual of operations, and will manage each participant's hypertension treatment. For all participants recruited, the CCNs and clinical sites will be responsible for achieving the goals specified in the protocol for adherence to study treatment and retention of study participants. The CCN will have the primary responsibility for overseeing their clinical sites and timely evaluation and correction of recruitment, adherence, and retention problems, including development and implementation of alternative strategies to achieve the stipulated goals, and funding the related activities. It is anticipated that each CCN will conduct periodic site visits within its network of clinical sites to supervise recruitment, adherence, and retention activities and to ensure high quality performance. The CCN activities will be coordinated with the CC, and may include site visits conducted by the CC, along with other organizational components of the study. The CCNs will collaborate closely with and assist the CC in implementation and standardization of the protocol within its network.

13.3 The Coordinating Center

The CC, with input from the SPRINT Steering Committee, will be responsible for coordinating protocol writing activities, including protocol drafting and finalization; developing and distributing forms and the MOP; training trial personnel in standardized protocol implementation and data collection; generating and distributing numerous reports (including specific recruitment goals and projections); providing rapid feedback to the CCN and Central Units on the quality of data submitted and proposed corrections;

developing and maintaining trial databases and related internal and public websites; collecting, managing, and analyzing all trial data; developing and overseeing the web-based adjudication of clinical events and endpoints; preparing reports for the DSMB; ensuring that the provisions of the manual of operations are carried out by all investigating groups; and providing timely and high quality statistical analysis expertise as required to prepare presentations and manuscripts. The CC will conduct periodic visits to each CCN in order to monitor and ensure high performance throughout the trial.

The CC will oversee 4 Central Units: the Drug Distribution Center, the Central Laboratory, the ECG Reading Center, and the MRI Reading Center.

The Central Laboratory will serve as a repository for immediate and future analyses of urine and blood specimens. The Central Laboratory will be responsible for the development and distribution of specific measurement procedures, and laboratory analyses, and for participating in quality assurance activities related to laboratory measures. Periodic reports will be generated to address sample acquisition quality for each clinical site and assay performance, and these will be provided to the CCNs and the Measurements, Procedures and Quality Control (MPQC) Subcommittee for review.

The ECG Reading Center will provide central interpretation of ECGs. The ECG Reading Center will develop procedures for obtaining and transmitting ECG data from the clinical sites to ensure the highest quality data collection. Periodic reports will be generated to address ECG quality for each clinical site, and these will be provided to the CCNs and the MPQC for review.

In collaboration with each CCN participating in the MRI study, the MRI Reading Center will identify an MRI site which is located in geographic proximity to the CCN's clinical sites. The MRI Reading Center will develop a detailed protocol and manual of procedures to ensure that the MRIs taken over time are of the highest quality with the smallest variation due to changes in technique and to allow the most precise estimate of change over time. The MRI Reading Center will provide training and certification for MRI site staff in order to ensure uniformity of methods, and will monitor carefully the quality of their work. Working with the CC, the MRI Reading Center will develop an analytical plan to estimate as precisely as possible the change in brain MRI over time for each SPRINT-MIND-MRI participant. Periodic reports will be generated to address MRI quality for each scanning site, and these will be provided to the CCNs and the MPQC for review.

The Drug Distribution Center will be responsible for developing and implementing plans for cost-effective drug acquisition; packaging, labeling, and dispensing drugs according to the study protocol; and providing data to the CC for further analyses. The DDC will design the technical aspects of drug packaging and labeling to facilitate participants' ability to understand and adhere to the drug regimen. The DDC will work with the clinical sites and CCNs to develop cost-effective inventory management procedures.

13.4 NHLBI Project Office and Other Government Representatives

The NHLBI Project Office will be responsible for the scientific conduct and administration of SPRINT. Representatives from the Project Office participate in the scientific, general organizational and fiscal management of the trial. NHLBI staff includes scientific representation from the Project Office team and members of the Office of Acquisitions

and the Office of Biostatistics Research. In addition, the NIH SPRINT team includes scientific staff from the NIDDK, the NINDS and the NIA.

13.5 The SPRINT Steering Committee, Executive Committee, Conflict of Interest Committee and the Subcommittees of the Steering Committee

The SPRINT Steering Committee provides the overall leadership for the study and establishes scientific and administrative policy. It is composed of the Principal Investigators from the five Clinical Center Networks, the Principal Investigator from the Coordinating Center, the NHLBI Project Officer, representatives from NIDDK, NINDS, NIA, the Steering Committee Chair, and the Steering Committee Vice-Chair. This committee oversees the overall conduct of the trial throughout all phases, develops the trial design, prepares the final protocol, and approves the study forms and manual of operations. During the data collection phases of the trial, this committee oversees data collection practices and procedures to identify and correct deficiencies. The Steering Committee also will consider and adopt changes in the study protocol or procedures as necessary during the course of the trial.

The SPRINT Steering Committee is chaired by the Steering Committee Chair, who serves as the senior executive officer of the investigative group. A Vice-Chair assists the Chair with Steering Committee responsibilities. Voting Steering Committee members are the Principal Investigators from the five CCNs, the Principal Investigator from the Coordinating Center, and the NHLBI Project Officer. If a CCN PI or the CC PI cannot make a meeting at which a vote is taken, then the Co-Principal Investigator may vote (with the understanding that the Co-PI is fully informed about the issue). The Steering Committee Chair, or Vice-Chair in his/her absence, votes only to break a tie. CCN and Site Co-investigators and Coordinators, CC staff, NIH staff, consultants, and opinion leaders may also be invited to attend meetings.

The SPRINT Executive Committee will oversee the day-to-day operations of the trial as an extension of the Steering Committee to ensure efficient and quality performance. The members include the Steering Committee Chair, Steering Committee Vice-Chair, Coordinating Center personnel, Project Office personnel, and one CCN PI (rotated annually so that each PI has the opportunity to serve). Other key study personnel (e.g., Chair of the Operations/Project Coordinators Subcommittee, Director of the DDC) may be asked to participate as either ad hoc or regular members.

The SPRINT Conflict of Interest Committee reviews potential conflict of interest issues. The NIH Project Office, Steering Committee Chair, and CC PI comprise this committee, which has the overall responsibility for the trial’s ethical oversight policy and procedures.

There are a number of standing subcommittees and working groups which report to the Steering Committee. These subcommittees and groups and their charges are detailed in Appendix 5.

13.6 The Data and Safety Monitoring Board

An independent Data and Safety Monitoring Board will be established to monitor data and oversee participant safety. Members will be appointed by the NHLBI to provide oversight of the trial and its ancillary studies. The SPRINT DSMB may include experts in cardiovascular medicine (particularly hypertension), kidney disease, clinical trials,

geriatrics, biostatistics, bioethics, quality of life, cost effectiveness, cognitive function and other areas as needed. DSMB participants include the Steering Committee Chair (who is unblinded) and Vice-Chair (who is blinded), CC PI and senior staff, and representatives from the NHLBI and other NIH sponsors. The DSMB normally meets twice a year to monitor safety, to advise the NHLBI about study progress, including contractor performance, and to make recommendations to the NHLBI regarding study continuation and protocol changes. In addition, the CC may provide data to the DSMB Chair to ensure early identification of any major adverse outcomes of therapy. The DSMB has the responsibility to recommend to the NHLBI whether the trial should continue, whether the protocol should be modified, or whether there should be early termination. The DSMB will provide reports to the NHLBI through the Executive Secretary, who will be appointed by the NHLBI. Recommendations by the DSMB must be approved by the NHLBI prior to implementation.

13.7 Role of Industry

Industry may contribute resources to the study and will be acknowledged appropriately. However, the scientific decisions and governance of the trial will be determined by the Steering Committee, as per NHLBI Policy.

13.8 Conflict of Interest Policy

The SPRINT investigators have established a policy regarding Conflict of Interest, which is presented in the MOP. This policy was developed to meet two goals. First, the investigators wished to maintain the confidence that advice was being given, and decisions made, in as unbiased and fully informed manner as possible. Second, the investigators wished that the processes and results of the trial would meet public standards of conduct.

13.9 Timeline

SPRINT will begin recruiting and randomizing during the fall of 2010. Recruitment will continue for approximately two years. The minimum length of participant planned follow-up will be four years, and maximum length of follow-up will be approximately six years, so the final study visits will occur in late 2016 or early 2017. If the event rate in the standard therapy arm is substantially less than 2.2%, we may ask that the DSMB consider recommending a two year extension of the trial.

13.10 Ancillary Studies

13.10.1 Introduction

In addition to the main SPRINT protocol, investigators may wish to perform Ancillary Studies using the SPRINT population, blood or urine samples, or other collected data. An ancillary study is an investigation not initiated by the SPRINT Steering Committee, with objectives that are not within the main SPRINT specific objectives and not part of the SPRINT protocol but uses SPRINT participants, samples, and/or data collected by SPRINT. In most cases, an ancillary study will involve acquisition of additional data that are not compiled as part of the SPRINT data set. An ancillary study may or may not use all randomized participants. Investigators are encouraged to propose and conduct ancillary studies. Such studies enhance the value and productivity of SPRINT and help

ensure the continued interest of the diverse group of investigators who are critical to the success of the trial as a whole. These studies provide an exceptional opportunity for investigators, either within or outside of SPRINT, to conduct additional projects at relatively low cost. In general, ancillary studies will require additional funding from the NIH or other sources.

13.10.2 Application Review Process

To protect the integrity of SPRINT, all ancillary studies must be reviewed and approved by the SPRINT Steering Committee before access to SPRINT data, samples, or participants is permitted. Investigators will not be allowed access to the SPRINT participants, samples, or database without approval. New ancillary study proposals will be submitted to the SPRINT Ancillary Science (AS) Subcommittee, which will review all ancillary study proposals and make a recommendation to the Steering Committee. In the event that investigators wish to modify an ancillary science protocol that have already been approved by the SPRINT SC, they will need to first obtain AS Subcommittee and SC approval. Ancillary study forms can be obtained by contacting the Coordinating Center or accessing the SPRINT website.

Studies submitted for approval less than four months prior to a funding application deadline may not receive timely approval. When the application is complete, the study proposal will be sent to the AS Subcommittee for review. The AS Subcommittee will have monthly calls to discuss proposals, which will be circulated at least one week prior to the calls. After review and approval by the AS Subcommittee, preliminary approval/disapproval will be made by the Steering Committee, with a final recommendation for approval/disapproval made by the Data and Safety Monitoring Board to the NHLBI Director. Ancillary Science investigators must include one or more SPRINT investigators in their ancillary study proposals.

The Coordinating Center will usually be responsible for all data management and analysis for all ancillary studies. Specialized expertise external to the coordinating center (e.g., processing of images) may be needed at the coordinating center's discretion. Costs associated with ancillary study data management and analysis must be budgeted into each ancillary study, even if the applicants have the necessary expertise in data management and analysis.

Prior to grant submission (or study initiation if no external funding is required), the CCN PI must approve participation of sites in her/his network. This is required as the CCN PI is responsible for the conduct of all aspects of SPRINT within her/his network. Part of this is management and oversight of clinic and participant burden. As needed, the CCN will include funding for oversight (e.g., investigator, coordinator, and fiscal personnel time, travel). The SPRINT Steering Committee also reserves the right to review the burden of ancillary studies on an on-going basis and take appropriate actions as necessary. Investigators with approved ancillary studies will report the status of the studies annually to the Chair of the AS Subcommittee.

Additional detail on the review process and criteria for judging proposals can be found in the MOP.

13.10.3 Additional Requirements of Ancillary Science Investigators

All ancillary study investigators will be required to budget adequately for all necessary resources for their studies. This includes, but may not be limited to, costs for data collection, sample collection, sample shipping, sample extraction, sample analysis, data entry, website development, data analysis, dataset preparation, data storage and publication of results. The final budget may be determined after AS and SC approval.

Each ancillary study will cause an increase in utilization of main SPRINT study resources, particularly by the SPRINT Presentations and Publications (P&P)Subcommittee. To help with study operations, each ancillary science proposal team should budget for and may be asked to contribute efforts to the main SPRINT study by, for example, assigning a person to serve as a reviewer for the P&P Subcommittee.

Investigators proposing the use of laboratory measurements are encouraged to use the SPRINT Central Laboratory if at all possible. This will facilitate sample processing and shipping and may reduce the amount of sample required.

All images (e.g., MRI) or tracings (e.g., ECG) must be available for other investigators to use in the spirit of the NIH policy available at http://grants.nih.gov/grants/sharing.htm. To achieve this goal, ancillary studies must budget for the costs associated with archiving these images and making them available to others. If there are legitimate reasons why this cannot be accomplished, this can be discussed on a case-by-case basis by the investigators, the funding agency, and the SPRINT SC.

13.11 Publication Policy

The purpose of the policy is to encourage and facilitate the presentation and publication of SPRINT Study background, rationale, design, and analyses; ensure appropriate use of the SPRINT data, timely completion of manuscripts and presentations, equitable access to authorship, and adherence to established principles of authorship; and coordinate the reporting of trial results. The policy applies to all investigators analyzing, presenting, and publishing data from main SPRINT, SPRINT-MIND, SPRINT-Senior (hereafter collectively called “SPRINT”)and ancillary studies, except for those using the NHLBI Data Repository data (see *https://biolincc.nhlbi.nih.gov/home/*).

There are several principles underlying this policy:

1. Research questions and hypotheses to be addressed using SPRINT Study data should be formulated *a priori* and clearly stated in a manuscript proposal to reduce the likelihood that study results are attributable to type I error.

2. Publication of scientific findings from the SPRINT Study should proceed in a timely fashion once relevant analyses are complete.

3. The publications arising from the SPRINT Study should avoid overlap and conflicting representation of SPRINT Study findings. Overlaps are, however, acceptable for review articles.

4. Recognition through authorship will be distributed among the SPRINT investigators so that:
    i) all SPRINT investigators and team members have equitable opportunity to lead and co-author SPRINT publications and, if appropriate, publications from ancillary studies;
    ii) all Ancillary Study investigators have the opportunity to lead and be co-authors on publications resulting from their ancillary studies.

5. The SPRINT Study should promote the career development of trainees and junior faculty by providing them the opportunity to lead and be recognized as co-authors of SPRINT publications, as appropriate.

6. Standards for authorship on SPRINT publications will adhere to the Uniform Requirements for Manuscripts Submitted to Biomedical Journals of the International Committee of Medical Journal Editors (NEJM 1997;336:309-315) and those established by the destination journals.

7. The concept, in the form of a proposal, for all manuscripts must be approved by the P&P Subcommittee prior to preparation.

There are three categories of manuscripts and anticipated authorship:

i) Main results developed based on core SPRINT data and study aims/hypotheses (which will bear the corporate authorship, "The SPRINT Research Group"). The design and main baseline papers will also be corporate authored.

ii) Manuscripts developed and authored by investigators using data that are not considered to be main SPRINT results.

iii) Ancillary study results led by investigators bringing external funding or resources into SPRINT for a specific project.

    (1) Unless specific justifications and alternative arrangements are made, all SPRINT analyses will be performed by the Coordinating Center (CC), with specialized expertise external to the Coordinating Center as needed at the Coordinating Center's discretion. Ancillary study budgets should include funds allocated to the CC for that purpose.

    (2) Ancillary study manuscripts are subject to similar review and tracking procedures as other SPRINT manuscripts.

During the operational phase of the trial, manuscripts proposing to use data other than baseline data will be reviewed closely to ensure that the SPRINT study objectives are not compromised. In general, the following will not be allowed:

(1) Publication of follow-up data according to randomized group

(2) Longitudinal analyses of outcomes pre-specified in the main protocol

All such proposals will be considered on a case-by-case basis, with input from the Data and Safety Monitoring Board.

The final responsibility for review and approval of manuscript proposals, including composition of writing committees, readiness for submission, and abstracts and material for presentations at meetings and conferences, rests with the Steering Committee. The P&P Subcommittee will oversee and facilitate these processes, assisted by a Publications Coordinator based at the Coordinating Center.

Figure 13.1: SPRINT Organizational Chart

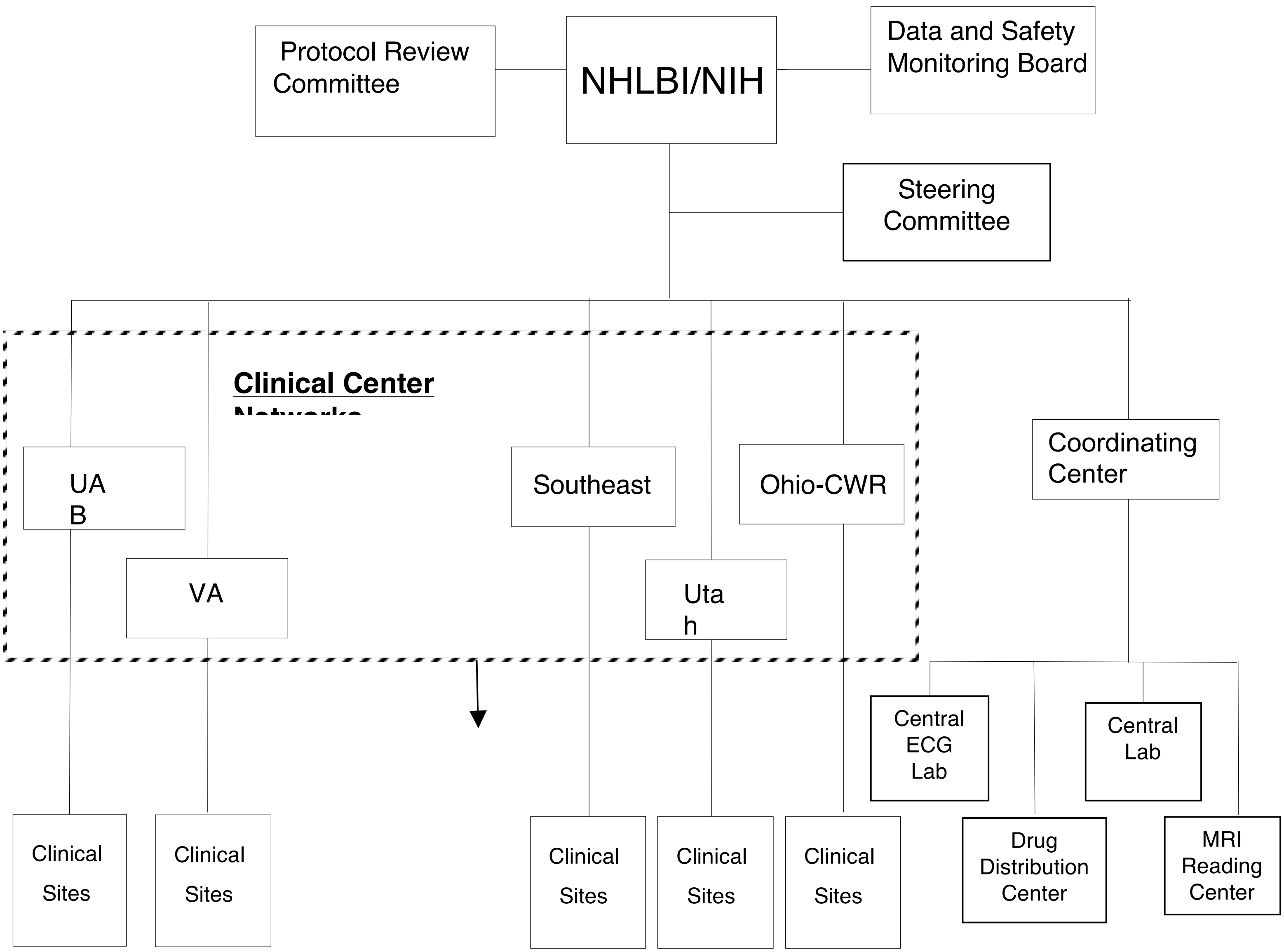

Reference List

1. Working Group Report: Expert Panel on a Hypertension Treatment Trial Initiative Meeting Summary. 2007.
Ref Type: Internet Communication

2. Abernethy, J., Borhani, N. O., Hawkins, C. M., Crow, R., Entwisle, G., Jones, J. W., Maxwell, M. H., Langford, H., and Pressel, S., 1986, Systolic blood pressure as an independent predictor of mortality in the Hypertension Detection and Follow-up Program: Am.J Prev.Med, v. 2, p. 123-132.

3. ALLHAT, 2002, Major outcomes in high-risk hypertensive patients randomized to angiotensin-converting enzyme inhibitor or calcium channel blocker vs diuretic: The Antihypertensive and Lipid-Lowering Treatment to Prevent Heart Attack Trial (ALLHAT): JAMA, v. 288, p. 2981-2997.

4. ALLHAT, 2003, Diuretic versus alpha-blocker as first-step antihypertensive therapy: final results from the Antihypertensive and Lipid-Lowering Treatment to Prevent Heart Attack Trial (ALLHAT): Hypertension, v. 42, p. 239-246.

5. Appel, L. J., Wright, J. T., Jr., Greene, T., Kusek, J. W., Lewis, J. B., Wang, X., Lipkowitz, M. S., Norris, K. C., Bakris, G. L., Rahman, M., Contreras, G., Rostand, S. G., Kopple, J. D., Gabbai, F. B., Schulman, G. I., Gassman, J. J., Charleston, J., and Agodoa, L. Y., 2008, Long-term effects of renin-angiotensin system-blocking therapy and a low blood pressure goal on progression of hypertensive chronic kidney disease in African Americans: Archives of Internal Medicine, v. 168, p. 832-839.

6. Ay, H., Benner, T., Arsava, E. M., Furie, K. L., Singhal, A. B., Jensen, M. B., Ayata, C., Towfighi, A., Smith, E. E., Chong, J. Y., Koroshetz, W. J., and Sorensen, A. G., 2007, A computerized algorithm for etiologic classification of ischemic stroke - The causative classification of stroke system: Stroke, v. 38, p. 2979-2984.

7. Bakris, G. L., Williams, M., Dworkin, L., Elliott, W. J., Epstein, M., Toto, R., Tuttle, K., Douglas, J., Hsueh, W., and Sowers, J., 2000, Preserving renal function in adults with hypertension and diabetes: a consensus approach. National Kidney Foundation Hypertension and Diabetes Executive Committees Working Group: Am.J Kidney Dis., v. 36, p. 646-661.

8. Basile, A. M., Pantoni, L., Pracucci, G., Asplund, K., Chabriat, H., Erkinjuntti, T., Fazekas, F., Ferro, J. M., Hennerici, M., O'brien, J., Scheltens, P., Visser, M. C., Wahlund, L. O., Waldemar, G., Wallin, A., and Inzitari, D., 2006, Age, hypertension, and lacunar stroke are the major determinants of the severity of age-related white matter changes. The LADIS (Leukoaraiosis and Disability in the Elderly) Study: Cerebrovasc.Dis., v. 21, p. 315-322.

9. Beckett, N. S., Peters, R., Fletcher, A. E., Staessen, J. A., Liu, L., Dumitrascu, D., Stoyanovsky, V., Antikainen, R. L., Nikitin, Y., Anderson, C.,

Belhani, A., Forette, F., Rajkumar, C., Thijs, L., Banya, W., and Bulpitt, C. J., 2008, Treatment of hypertension in patients 80 years of age or older: N Engl J Med, v. 358, p. 1887-1898.

10. Berl, T., Hunsicker, L. G., Lewis, J. B., Pfeffer, M. A., Porush, J. G., Rouleau, J. L., Drury, P. L., Esmatjes, E., Hricik, D., Pohl, M., Raz, I., Vanhille, P., Wiegmann, T. B., Wolfe, B. M., Locatelli, F., Goldhaber, S. Z., Lewis, E. J., and for the Collaborative Study Group, 2005, Impact of Achieved Blood Pressure on Cardiovascular Outcomes in the Irbesartan Diabetic Nephropathy Trial: Journal of the American Society of Nephrology, v. 16, p. 2170-2179.

11. Berlowitz, D. R., Ash, A. S., Hickey, E. C., Friedman, R. H., Glickman, M., Kader, B., and Moskowitz, M. A., 1998, Inadequate management of blood pressure in a hypertensive population: New England Journal of Medicine, v. 339, p. 1957-1963.

12. Bharmal, M., Payne, K., Atkinson, M. J., Desrosiers, M. P., Morisky, D. E., and Gemmen, E., 2009, Validation of an abbreviated Treatment Satisfaction Questionnaire for Medication (TSQM-9) among patients on antihypertensive medications: Health Qual.Life Outcomes., v. 7, p. 36.

13. Birns, J., Morris, R., Donaldson, N., and Kalra, L., 2006, The effects of blood pressure reduction on cognitive function: a review of effects based on pooled data from clinical trials
BIRNS2006: J.Hypertens., v. 24, p. 1907-1914.

14. Brandt, J. and Benedict, R. H. B., 2001, Hopkins Verbal Learning Test-Revised. Professional manual: Lutz, FL, Psychological Assessment Resources, Inc.

15. Braunwald, E., Domanski, M. J., Fowler, S. E., Geller, N. L., Gersh, B. J., Hsia, J., Pfeffer, M. A., Rice, M. M., Rosenberg, Y. D., and Rouleau, J. L., 2004, Angiotensin-converting-enzyme inhibition in stable coronary artery disease: N Engl J Med., v. 351, p. 2058-2068.

16. Brookmeyer, R., Corrada, M. M., Curriero, F. C., and Kawas, C., 2002, Survival following a diagnosis of Alzheimer disease: Arch.Neurol., v. 59, p. 1764-1767.

17. Calhoun, D. A., Jones, D., Textor, S., Goff, D. C., Murphy, T. P., Toto, R. D., White, A., Cushman, W. C., White, W., Sica, D., Ferdinand, K., Giles, T. D., Falkner, B., and Carey, R. M., 2008, Resistant hypertension: Diagnosis, evaluation, and treatment - A Scientific Statement from the American Heart Association Professional Education Committee of the Council for High Blood Pressure Research: Hypertension, v. 51, p. 1403-1419.

18. Calhoun, D. A., Lacourciere, Y., Chiang, Y. T., and Glazer, R. D., 2009, Triple Antihypertensive Therapy With Amlodipine, Valsartan, and Hydrochlorothiazide A Randomized Clinical Trial: Hypertension, v. 54, p. 32-39.

19. Campbell, N. R., Khan, N. A., Hill, M. D., Tremblay, G., Lebel, M., Kaczorowski, J., McAlister, F. A., Lewanczuk, R. Z., and Tobe, S., 2009, 2009 Canadian Hypertension Education Program recommendations: the scientific summary--an annual update: Can.J Cardiol., v. 25, p. 271-277.

20. Chobanian, A. V., Bakris, G. L., Black, H. R., Cushman, W. C., Green, L. A., Izzo, J. L., Jr., Jones, D. W., Materson, B. J., Oparil, S., Wright, J. T., Jr., and Roccella, E. J., 2003, The Seventh Report of the Joint National Committee on Prevention, Detection, Evaluation, and Treatment of High Blood Pressure: the JNC 7 report: JAMA, v. 289, p. 2560-2572.

21. Coca, S. G., Krumholz, H. M., Garg, A. X., and Parikh, C. R., 2006, Underrepresentation of renal disease in randomized controlled trials of cardiovascular disease: JAMA, v. 296, p. 1377-1384.

22. Coker L.H., Hogan P.E., Bryan N.R., and et al. Effects of postmenopausal hormone therapy on volumetric sub-clinical cerebrovascular disease: The Women's Health Initiative Memory Study - Magnetic Resonance Imaging Study (WHIMS-MRI). 2008.
Ref Type: Unpublished Work

23. Collins, R., Peto, R., Macmahon, S., Hebert, P., Fiebach, N. H., Eberlein, K. A., Godwin, J., Qizilbash, N., Taylor, J. O., and Hennekens, C. H., 1990, Blood pressure, stroke, and coronary heart disease. Part 2, Short-term reductions in blood pressure: overview of randomised drug trials in their epidemiological context: Lancet, v. 335, p. 827-838.

24. Coresh, J., Selvin, E., Stevens, L. A., Manzi, J., Kusek, J. W., Eggers, P., Van Lente, F., and Levey, A. S., 2007, Prevalence of chronic kidney disease in the United States: JAMA, v. 298, p. 2038-2047.

25. Cox, D. R., 1972, Regression models and life-tables: Journal of the Royal Statistical Society, Series B: Methodological, v. 34, p. 187-220.

26. Cruickshank, J. M., 2000, Antihypertensive treatment and the J-curve: Cardiovasc.Drugs Ther., v. 14, p. 373-379.

27. Cruickshank, J. M., Pennert, K., Sorman, A. E., Thorp, J. M., Zacharias, F. M., and Zacharias, F. J., 1987, Low mortality from all causes, including myocardial infarction, in well-controlled hypertensives treated with a beta-blocker plus other antihypertensives: J Hypertens., v. 5, p. 489-498.

28. Cushman, W. C., Ford, C. E., Cutler, J. A., Margolis, K. L., Davis, B. R., Grimm, R. H., Black, H. R., Hamilton, B. P., Holland, J., Nwachuku, C., Papademetriou, V., Probstfield, J., Wright, J. T., Jr., Alderman, M. H., Weiss, R. J., Piller, L., Bettencourt, J., and Walsh, S. M., 2002, Success and predictors of blood pressure control in diverse North American settings: the antihypertensive and lipid-lowering treatment to prevent heart attack trial (ALLHAT): J.Clin.Hypertens.(Greenwich.), v. 4, p. 393-404.

29. Cushman, W. C., Ford, C. E., Einhorn, P. T., Wright, J. T., Preston, R. A., Davis, B. R., Basile, J. N., Whelton, P. K., Weiss, R. J., Bastien, A., Courtney, D. L., Hamilton, B. P., Kirchner, K., Louis, G. T., Retta, T. M., and Vidt, D. G., 2008, Blood Pressure Control by Drug Group in the Antihypertensive and Lipid-Lowering Treatment to Prevent Heart Attack Trial (ALLHAT): Journal of Clinical Hypertension, v. 10, p. 751-760.

30. Cushman, W. C., Grimm, R. H., Cutler, J. A., Evans, G. W., Capes, S., Corson, M. A., Sadler, L. S., Alderman, M. H., Peterson, K., Bertoni, A., and Basile, J. N., 2007, Rationale and design for the blood pressure intervention of the Action to Control Cardiovascular Risk in Diabetes (ACCORD) trial: American Journal of Cardiology, v. 99, p. 44I-55I.

31. Cutler, J. A., MacMahon, S. W., and Furberg, C. D., 1989, Controlled clinical trials of drug treatment for hypertension. A review: Hypertension, v. 13, p. I36-I44.

32. Cutler, J. A., Sorlie, P. D., Wolz, M., Thom, T., Fields, L. E., and Roccella, E. J., 2008, Trends in Hypertension Prevalence, Awareness, Treatment, and Control Rates in United States Adults Between 1988-1994 and 1999-2004: Hypertension, v. 52, p. 818-827.

33. Debette, S., Beiser, A., DeCarli, C., Au, R., Himali, J. J., Kelly-Hayes, M., Romero, J. R., Kase, C. S., Wolf, P. A., and Seshadri, S., 2010, Association of MRI markers of vascular brain injury with incident stroke, mild cognitive impairment, dementia, and mortality: the Framingham Offspring Study: Stroke, v. 41, p. 600-606.

34. DeKosky, S. T., Williamson, J. D., Fitzpatrick, A. L., Kronmal, R. A., Ives, D. G., Saxton, J. A., Lopez, O. L., Burke, G., Carlson, M. C., Fried, L. P., Kuller, L. H., Robbins, J. A., Tracy, R. P., Woolard, N. F., Dunn, L., Snitz, B. E., Nahin, R. L., Furberg, C. D., and for the Ginkgo Evaluation of Memory (GEM) Study Investigators, 2008, Ginkgo biloba for Prevention of Dementia: A Randomized Controlled Trial: JAMA: The Journal of the American Medical Association, v. 300, p. 2253-2262.

35. Di Angelantonio, E., Danesh, J., Eiriksdottir, G., and Gudnason, V., 2007, Renal function and risk of coronary heart disease in general populations: new prospective study and systematic review: PLoS.Med, v. 4, p. e270.

36. Di Bari, M., Pahor, M., Franse, L. V., Shorr, R. I., Ferrucci, L., Wan, J. Y., Somes, G. W., and Applegate, W. B., 2001, Dementia and disability outcomes in large hypertension trials: lessons learned from the Systolic Hypertension in the Elderly Program (SHEP) Trial: Am.J.Epidemiol., v. 153, p. 72-78.

37. Dufouil, C., Chalmers, J., Coskun, O., Besancon, V., Bousser, M. G., Guillon, P., Macmahon, S., Mazoyer, B., Neal, B., Woodward, M., Tzourio-Mazoyer, N., and Tzourio, C., 2005, Effects of blood pressure lowering on cerebral white matter hyperintensities in patients with stroke: the

PROGRESS (Perindopril Protection Against Recurrent Stroke Study) Magnetic Resonance Imaging Substudy: Circulation, v. 112, p. 1644-1650.

38. Dufouil, C., Godin, O., Chalmers, J., Coskun, O., Macmahon, S., Tzourio-Mazoyer, N., Bousser, M. G., Anderson, C., Mazoyer, B., and Tzourio, C., 2009, Severe cerebral white matter hyperintensities predict severe cognitive decline in patients with cerebrovascular disease history: Stroke, v. 40, p. 2219-2221.

39. Elias, M. F., Wolf, P. A., D'Agostino, R. B., Cobb, J., and White, L. R., 1993, Untreated blood pressure level is inversely related to cognitive functioning: the Framingham Study: Am.J.Epidemiol., v. 138, p. 353-364.

40. Ellis, R. J., Jan, K., Kawas, C., Koller, W. C., Lyons, K. E., Jeste, D. V., Hansen, L. A., and Thal, L. J., 1998, Diagnostic validity of the dementia questionnaire for Alzheimer disease: Arch.Neurol., v. 55, p. 360-365.

41. Ernst, M. E., Carter, B. L., Goerdt, C. J., Steffensmeier, J. J. G., Phillips, B. B., Zimmerman, M. B., and Bergus, G. R., 2006, Comparative antihypertensive effects of hydrochlorothiazide and chlorthalidone on ambulatory and office blood pressure: Hypertension, v. 47, p. 352-358.

42. Ferri, C. P., Prince, M., Brayne, C., Brodaty, H., Fratiglioni, L., Ganguli, M., Hall, K., Hasegawa, K., Hendrie, H., Huang, Y., Jorm, A., Mathers, C., Menezes, P. R., Rimmer, E., and Scazufca, M., 2005, Global prevalence of dementia: a Delphi consensus study: Lancet, v. 366, p. 2112-2117.

43. Forette, F., Seux, M. L., Staessen, J. A., Thijs, L., Birkenhager, W. H., Babarskiene, M. R., Babeanu, S., Bossini, A., Gil-Extremera, B., Girerd, X., Laks, T., Lilov, E., Moisseyev, V., Tuomilehto, J., Vanhanen, H., Webster, J., Yodfat, Y., and Fagard, R., 1998, Prevention of dementia in randomised double-blind placebo-controlled Systolic Hypertension in Europe (Syst-Eur) trial: Lancet, v. 352, p. 1347-1351.

44. Foster, M. C., Hwang, S. J., Larson, M. G., Parikh, N. I., Meigs, J. B., Vasan, R. S., Wang, T. J., Levy, D., and Fox, C. S., 2007, Cross-classification of microalbuminuria and reduced glomerular filtration rate: associations between cardiovascular disease risk factors and clinical outcomes: Archives of Internal Medicine, v. 167, p. 1386-1392.

45. Fox, K. M., 2003, Efficacy of perindopril in reduction of cardiovascular events among patients with stable coronary artery disease: randomised, double-blind, placebo-controlled, multicentre trial (the EUROPA study): Lancet., v. 362, p. 782-788.

46. Franklin, S. S., 1999, Cardiovascular risks related to increased diastolic, systolic and pulse pressure. An epidemiologist's point of view: Pathol.Biol.(Paris), v. 47, p. 594-603.

47. Franklin, S. S., Jacobs, M. J., Wong, N. D., L'Italien, G. J., and Lapuerta, P., 2001, Predominance of isolated systolic hypertension among middle-aged

and elderly US hypertensives: analysis based on National Health and Nutrition Examination Survey (NHANES) III: Hypertension, v. 37, p. 869-874.

48. Freitag, M. H., Peila, R., Masaki, K., Petrovitch, H., Ross, G. W., White, L. R., and Launer, L. J., 2006, Midlife pulse pressure and incidence of dementia: the Honolulu-Asia Aging Study: Stroke, v. 37, p. 33-37.

49. Fried, L. F., Katz, R., Cushman, M., Sarnak, M., Shlipak, M. G., Kuller, L., and Newman, A. B., 2009, Change in cardiovascular risk factors with progression of kidney disease: Am.J Nephrol., v. 29, p. 334-341.

50. Gillum, R. F., 1991, Cardiovascular disease in the United States: an epidemiologic overview: Cardiovasc.Clin., v. 21, p. 3-16.

51. Go, A. S., Chertow, G. M., Fan, D., McCulloch, C. E., and Hsu, C. Y., 2004, Chronic kidney disease and the risks of death, cardiovascular events, and hospitalization: N.Engl.J.Med., v. 351, p. 1296-1305.

52. Greenlund, K. J., Croft, J. B., and Mensah, G. A., 2004, Prevalence of heart disease and stroke risk factors in persons with prehypertension in the United States, 1999-2000: Archives of Internal Medicine, v. 164, p. 2113-2118.

53. guero-Torres, H., von, S. E., Viitanen, M., Winblad, B., and Fratiglioni, L., 2001, Institutionalization in the elderly: the role of chronic diseases and dementia. Cross-sectional and longitudinal data from a population-based study: J.Clin.Epidemiol., v. 54, p. 795-801.

54. Guralnik, J. M., Leveille, S. G., Hirsch, R., Ferrucci, L., and Fried, L. P., 1997, The impact of disability in older women: J.Am.Med.Womens Assoc., v. 52, p. 113-120.

55. Hajjar, I. and Kotchen, T. A., 2003, Trends in prevalence, awareness, treatment, and control of hypertension in the United States, 1988-2000: JAMA, v. 290, p. 199-206.

56. Hancock, E. W., Deal, B. J., Mirvis, D. M., Okin, P., Kligfield, P., and Gettes, L. S., 2009, AHA/ACCF/HRS Recommendations for the Standardization and Interpretation of the Electrocardiogram Part V: Electrocardiogram Changes Associated With Cardiac Chamber Hypertrophy A Scientific Statement From the American Heart Association Electrocardiography and Arrhythmias Committee, Council on Clinical Cardiology; the American College of Cardiology Foundation; and the Heart Rhythm Society Endorsed by the International Society for Computerized Electrocardiology: Journal of the American College of Cardiology, v. 53, p. 992-1002.

57. Hansson, L., Zanchetti, A., Carruthers, S. G., Dahlof, B., Elmfeldt, D., Julius, S., Menard, J., Rahn, K. H., Wedel, H., and Westerling, S., 1998, Effects of intensive blood-pressure lowering and low-dose aspirin in patients with hypertension: principal results of the Hypertension Optimal Treatment (HOT) randomised trial. HOT Study Group: Lancet., v. 351, p. 1755-1762.

58. Haroun, M. K., Jaar, B. G., Hoffman, S. C., Comstock, G. W., Klag, M. J., and Coresh, J., 2003, Risk factors for chronic kidney disease: a prospective study of 23,534 men and women in Washington County, Maryland: J Am Soc Nephrol, v. 14, p. 2934-2941.

59. HDFP, 1979a, Five-year findings of the hypertension detection and follow-up program. I. Reduction in mortality of persons with high blood pressure, including mild hypertension. Hypertension Detection and Follow-up Program Cooperative Group: JAMA, v. 242, p. 2562-2571.

60. HDFP, 1979b, Five-year findings of the hypertension detection and follow-up program. II. Mortality by race-sex and age. Hypertension Detection and Follow-up Program Cooperative Group: JAMA, v. 242, p. 2572-2577.

61. HDFP, 1982, The effect of treatment on mortality in "mild" hypertension: results of the hypertension detection and follow-up program: The New England Journal of Medicine, v. 307, p. 976-980.

62. HOT, 1998, Cost effectiveness of intensive treatment of hypertension. Based on presentations by Donald S. Shepard, PhD; and Dominic Hodgkin, PhD: Am.J Manag.Care, v. 4, p. S765-S769.

63. Hsia, J., Margolis, K. L., Eaton, C. B., Wenger, N. K., Allison, M., Wu, L., LaCroix, A. Z., and Black, H. R., 2007, Prehypertension and cardiovascular disease risk in the Women's Health Initiative: Circulation, v. 115, p. 855-860.

64. Hsu, C. Y., McCulloch, C. E., Darbinian, J., Go, A. S., and Iribarren, C., 2005, Elevated blood pressure and risk of end-stage renal disease in subjects without baseline kidney disease: Archives of Internal Medicine, v. 165, p. 923-928.

65. Ikram, M. A., van, O. M., de Jong, F. J., Kors, J. A., Koudstaal, P. J., Hofman, A., Witteman, J. C., and Breteler, M. M., 2008, Unrecognized myocardial infarction in relation to risk of dementia and cerebral small vessel disease: Stroke, v. 39, p. 1421-1426.

66. Jackson, R., 2000, Guidelines on preventing cardiovascular disease in clinical practice: BMJ., v. 320, p. 659-661.

67. Julius, S., Alderman, M. H., Beevers, G., Dahlof, B., Devereux, R. B., Douglas, J. G., Edelman, J. M., Harris, K. E., Kjeldsen, S. E., Nesbitt, S., Randall, O. S., and Wright, J. T., 2004, Cardiovascular risk reduction in hypertensive black patients with left ventricular hypertrophy - The LIFE study: Journal of the American College of Cardiology, v. 43, p. 1047-1055.

68. Kaplan E, Goodglass F., and Weintraub S., 1983, Boston Naming Test: Philadelphia PA, Lea and Febiger.

69. Kawas, C., Segal, J., Stewart, W. F., Corrada, M., and Thal, L. J., 1994, A validation study of the Dementia Questionnaire: Arch.Neurol., v. 51, p. 901-906.

70. Kearney, P. M., Whelton, M., Reynolds, K., Muntner, P., Whelton, P. K., and He, J., 2005, Global burden of hypertension: analysis of worldwide data: The Lancet, v. 365, p. 217-223.

71. Keeley, E. C., Kadakia, R., Soman, S., Borzak, S., and McCullough, P. A., 2003, Analysis of long-term survival after revascularization in patients with chronic kidney disease presenting with acute coronary syndromes: Am.J Cardiol., v. 92, p. 509-514.

72. Kivipelto, M., Helkala, E. L., Hanninen, T., Laakso, M. P., Hallikainen, M., Alhainen, K., Soininen, H., Tuomilehto, J., and Nissinen, A., 2001a, Midlife vascular risk factors and late-life mild cognitive impairment: A population-based study: Neurology, v. 56, p. 1683-1689.

73. Kivipelto, M., Helkala, E. L., Hanninen, T., Laakso, M. P., Hallikainen, M., Alhainen, K., Soininen, H., Tuomilehto, J., and Nissinen, A., 2001b, Midlife vascular risk factors and late-life mild cognitive impairment: A population-based study: Neurology, v. 56, p. 1683-1689.

74. Kivipelto, M., Helkala, E. L., Laakso, M. P., Hanninen, T., Hallikainen, M., Alhainen, K., Soininen, H., Tuomilehto, J., and Nissinen, A., 2001c, Midlife vascular risk factors and Alzheimer's disease in later life: longitudinal, population based study: BMJ, v. 322, p. 1447-1451.

75. Klag, M. J., Whelton, P. K., Randall, B. L., Neaton, J. D., Brancati, F. L., Ford, C. E., Shulman, N. B., and Stamler, J., 1996, Blood pressure and end-stage renal disease in men: N.Engl.J.Med., v. 334, p. 13-18.

76. Klahr, S., Levey, A. S., Beck, G. J., Caggiula, A. W., Hunsicker, L., Kusek, J. W., and Striker, G., 1994, The effects of dietary protein restriction and blood-pressure control on the progression of chronic renal disease. Modification of Diet in Renal Disease Study Group: N.Engl.J.Med., v. 330, p. 877-884.

77. Knopman, D., Boland, L. L., Mosley, T., Howard, G., Liao, D., Szklo, M., McGovern, P., and Folsom, A. R., 2001, Cardiovascular risk factors and cognitive decline in middle-aged adults: Neurology, v. 56, p. 42-48.

78. Kshirsagar, A. V., Carpenter, M., Bang, H., Wyatt, S. B., and Colindres, R. E., 2006, Blood pressure usually considered normal is associated with an elevated risk of cardiovascular disease: Am.J Med, v. 119, p. 133-141.

79. Kuller, L. H., Margolis, K. L., Gaussoin, S. A., Bryan, N. R., Kerwin, D., Limacher, M., Wassertheil-Smoller, S., Williamson, J., and Robinson, J. G., 2010, Relationship of hypertension, blood pressure, and blood pressure control with white matter abnormalities in the Women's Health Initiative Memory Study (WHIMS)-MRI trial: J Clin.Hypertens.(Greenwich.), v. 12, p. 203-212.

80. Lachin, J. M. and Foulkes, M. A., 1986, Evaluation of sample size and power for analyses of survival with allowance for nonuniform patient entry, losses

to follow-up, noncompliance, and stratification: Biometrics, v. 42, p. 507-519.

81. Lakatos, E., 1988, Sample sizes based on the log-rank statistic in complex clinical trials: Biometrics, v. 44, p. 229-241.

82. Lawes, C. M., Vander, H. S., and Rodgers, A., 2008, Global burden of blood-pressure-related disease, 2001: Lancet, v. 371, p. 1513-1518.

83. Levey, A. S., Beto, J. A., Coronado, B. E., Eknoyan, G., Foley, R. N., Kasiske, B. L., Klag, M. J., Mailloux, L. U., Manske, C. L., Meyer, K. B., Parfrey, P. S., Pfeffer, M. A., Wenger, N. K., Wilson, P. W., and Wright, J. T., Jr., 1998, Controlling the epidemic of cardiovascular disease in chronic renal disease: what do we know? What do we need to learn? Where do we go from here? National Kidney Foundation Task Force on Cardiovascular Disease: Am.J Kidney Dis., v. 32, p. 853-906.

84. Levy, D., Larson, M. G., Vasan, R. S., Kannel, W. B., and Ho, K. K., 1996, The progression from hypertension to congestive heart failure: JAMA, v. 275, p. 1557-1562.

85. Lewington, S., Clarke, R., Qizilbash, N., Peto, R., and Collins, R., 2002, Age-specific relevance of usual blood pressure to vascular mortality: a meta-analysis of individual data for one million adults in 61 prospective studies: Lancet, v. 360, p. 1903-1913.

86. Liao, D., Cooper, L., Cai, J., Toole, J. F., Bryan, N. R., Hutchinson, R. G., and Tyroler, H. A., 1996, Presence and severity of cerebral white matter lesions and hypertension, its treatment, and its control. The ARIC Study. Atherosclerosis Risk in Communities Study: Stroke, v. 27, p. 2262-2270.

87. Lindholm, L. H., Carlberg, B., and Samuelsson, O., 2005, Should beta blockers remain first choice in the treatment of primary hypertension? A meta-analysis: Lancet, v. 366, p. 1545-1553.

88. Liu, L., Wang, J. G., Gong, L., Liu, G., and Staessen, J. A., 1998, Comparison of active treatment and placebo in older Chinese patients with isolated systolic hypertension. Systolic Hypertension in China (Syst-China) Collaborative Group: J Hypertens., v. 16, p. 1823-1829.

89. Longstreth, W. T., Jr., Manolio, T. A., Arnold, A., Burke, G. L., Bryan, N., Jungreis, C. A., Enright, P. L., O'Leary, D., and Fried, L., 1996, Clinical correlates of white matter findings on cranial magnetic resonance imaging of 3301 elderly people. The Cardiovascular Health Study: Stroke, v. 27, p. 1274-1282.

90. Luchsinger, J. A. and Mayeux, R., 2004, Cardiovascular risk factors and Alzheimer's disease: Curr.Atheroscler.Rep., v. 6, p. 261-266.

91. Luepker, R. V., Apple, F. S., Christenson, R. H., Crow, R. S., Fortmann, S. P., Goff, D., Goldberg, R. J., Hand, M. M., Jaffe, A. S., Julian, D. G., Levy, D.,

Manolio, T., Mendis, S., Mensah, G., Pajak, A., Prineas, R. J., Reddy, K. S., Roger, V. L., Rosamond, W. D., Shahar, E., Sharrett, A. R., Sorlie, P., and Tunstall-Pedoe, H., 2003, Case Definitions for Acute Coronary Heart Disease in Epidemiology and Clinical Research Studies: A Statement From the AHA Council on Epidemiology and Prevention; AHA Statistics Committee; World Heart Federation Council on Epidemiology and Prevention; the European Society of Cardiology Working Group on Epidemiology and Prevention; Centers for Disease Control and Prevention; and the National Heart, Lung, and Blood Institute: Circulation, v. 108, p. 2543-2549.

92. Macmahon, S., Peto, R., Cutler, J., Collins, R., Sorlie, P., Neaton, J., Abbott, R., Godwin, J., Dyer, A., and Stamler, J., 1990, Blood pressure, stroke, and coronary heart disease. Part 1, Prolonged differences in blood pressure: prospective observational studies corrected for the regression dilution bias: Lancet., v. 335, p. 765-774.

93. Magsi, H. and Malloy, T., 2005, Underrecognition of cognitive impairment in assisted living facilities: J.Am.Geriatr.Soc., v. 53, p. 295-298.

94. Mancia, G., De, B. G., Dominiczak, A., Cifkova, R., Fagard, R., Germano, G., Grassi, G., Heagerty, A. M., Kjeldsen, S. E., Laurent, S., Narkiewicz, K., Ruilope, L., Rynkiewicz, A., Schmieder, R. E., Struijker Boudier, H. A., Zanchetti, A., Vahanian, A., Camm, J., De, C. R., Dean, V., Dickstein, K., Filippatos, G., Funck-Brentano, C., Hellemans, I., Kristensen, S. D., McGregor, K., Sechtem, U., Silber, S., Tendera, M., Widimsky, P., Zamorano, J. L., Kjeldsen, S. E., Erdine, S., Narkiewicz, K., Kiowski, W., gabiti-Rosei, E., Ambrosioni, E., Cifkova, R., Dominiczak, A., Fagard, R., Heagerty, A. M., Laurent, S., Lindholm, L. H., Mancia, G., Manolis, A., Nilsson, P. M., Redon, J., Schmieder, R. E., Struijker-Boudier, H. A., Viigimaa, M., Filippatos, G., Adamopoulos, S., gabiti-Rosei, E., Ambrosioni, E., Bertomeu, V., Clement, D., Erdine, S., Farsang, C., Gaita, D., Kiowski, W., Lip, G., Mallion, J. M., Manolis, A. J., Nilsson, P. M., O'Brien, E., Ponikowski, P., Redon, J., Ruschitzka, F., Tamargo, J., van, Z. P., Viigimaa, M., Waeber, B., Williams, B., Zamorano, J. L., The task force for the management of arterial hypertension of the European Society of Hypertension, and The task force for the management of arterial hypertension of the European Society of Cardiology, 2007, 2007 Guidelines for the management of arterial hypertension: The Task Force for the Management of Arterial Hypertension of the European Society of Hypertension (ESH) and of the European Society of Cardiology (ESC): Eur.Heart J, v. 28, p. 1462-1536.

95. Mancia, G., Laurent, S., gabiti-Rosei, E., Ambrosioni, E., Burnier, M., Caulfield, M. J., Cifkova, R., Clement, D., Coca, A., Dominiczak, A., Erdine, S., Fagard, R., Farsang, C., Grassi, G., Haller, H., Heagerty, A., Kjeldsen, S. E., Kiowski, W., Mallion, J. M., Manolis, A., Narkiewicz, K., Nilsson, P., Olsen, M. H., Rahn, K. H., Redon, J., Rodicio, J., Ruilope, L., Schmieder, R. E., Struijker-Boudier, H. A., Van Zwieten, P. A., Viigimaa, M., and Zanchetti, A., 2009, Reappraisal of European guidelines on hypertension management: a European Society of Hypertension Task Force document: J Hypertens..

96. McCullough, P. A., Jurkovitz, C. T., Pergola, P. E., McGill, J. B., Brown, W. W., Collins, A. J., Chen, S. C., Li, S., Singh, A., Norris, K. C., Klag, M. J., and Bakris, G. L., 2007, Independent components of chronic kidney disease as a cardiovascular risk state: results from the Kidney Early Evaluation Program (KEEP): Archives of Internal Medicine, v. 167, p. 1122-1129.

97. Nasreddine, Z. S., Phillips, N. A., Bedirian, V., Charbonneau, S., Whitehead, V., Collin, I., Cummings, J. L., and Chertkow, H., 2005, The montreal cognitive assessment, MoCA: A brief screening tool for mild cognitive impairment: Journal of the American Geriatrics Society, v. 53, p. 695-699.

98. National Collaborating Centre for Chronic Conditions. Hypertension: management in adults in primary care: pharmacological update. 2006. London, Royal College of Physicians.
Ref Type: Report

99. National Heart Foundation of Australia (National Blood Pressure and Vascular Disease Advisory Committee). Guide to management of hypertension 2008. 2009. *National Heart Foundation of Australia*.
Ref Type: Report

100. National Institute on Aging. Progress Report on Alzheimer's Disease. NIH Publication no. 00-4859. 2000. Department of Health and Human Services, Public Health Service.
Ref Type: Report

101. Neal, B., Macmahon, S., and Chapman, N., 2000, Effects of ACE inhibitors, calcium antagonists, and other blood-pressure-lowering drugs: results of prospectively designed overviews of randomised trials. Blood Pressure Lowering Treatment Trialists' Collaboration: Lancet, v. 356, p. 1955-1964.

102. Nilsson, S. E., Read, S., Berg, S., Johansson, B., Melander, A., and Lindblad, U., 2007, Low systolic blood pressure is associated with impaired cognitive function in the oldest old: longitudinal observations in a population-based sample 80 years and older: Aging Clin.Exp.Res., v. 19, p. 41-47.

103. Nissen, S. E., Tuzcu, E. M., Libby, P., Thompson, P. D., Ghali, M., Garza, D., Berman, L., Shi, H., Buebendorf, E., and Topol, E. J., 2004, Effect of antihypertensive agents on cardiovascular events in patients with coronary disease and normal blood pressure: the CAMELOT study: a randomized controlled trial: JAMA., v. 292, p. 2217-2225.

104. Ong, K. L., Cheung, B. M., Man, Y. B., Lau, C. P., and Lam, K. S., 2007, Prevalence, awareness, treatment, and control of hypertension among United States adults 1999-2004: Hypertension, v. 49, p. 69-75.

105. Petersen, R. C., 2000, Aging, mild cognitive impairment, and Alzheimer's disease: Neurol.Clin., v. 18, p. 789-806.

106. Peterson, J. C., Adler, S., Burkart, J. M., Greene, T., Hebert, L. A., Hunsicker, L. G., King, A. J., Klahr, S., Massry, S. G., and Seifter, J. L., 1995, Blood pressure control, proteinuria, and the progression of renal disease. The Modification of Diet in Renal Disease Study: Ann.Intern Med, v. 123, p. 754-762.

107. Pfeffer, R. I., Kurosaki, T. T., Harrah, C. H., Jr., Chance, J. M., and Filos, S., 1982, Measurement of functional activities in older adults in the community: J Gerontol., v. 37, p. 323-329.

108. Plassman, B. L., Langa, K. M., Fisher, G. G., Heeringa, S. G., Weir, D. R., Ofstedal, M. B., Burke, J. R., Hurd, M. D., Potter, G. G., Rodgers, W. L., Steffens, D. C., Willis, R. J., and Wallace, R. B., 2007, Prevalence of dementia in the United States: the aging, demographics, and memory study: Neuroepidemiology, v. 29, p. 125-132.

109. PROGRESS Collaborative Group, 2001, Randomised trial of a perindopril-based blood-pressure-lowering regimen among 6105 individuals with previous stroke or transient ischaemic attack: The Lancet, v. 358, p. 1033-1041.

110. Prospective Studies Collaboration, 2002, Age-specific relevance of usual blood pressure to vascular mortality: a meta-analysis of individual data for one million adults in 61 prospective studies: The Lancet, v. 360, p. 1903-1913.

111. Psaty, B. M., Smith, N. L., Siscovick, D. S., Koepsell, T. D., Weiss, N. S., Heckbert, S. R., Lemaitre, R. N., Wagner, E. H., and Furberg, C. D., 1997, Health outcomes associated with antihypertensive therapies used as first-line agents. A systematic review and meta-analysis: JAMA, v. 277, p. 739-745.

112. Qiu, C., Winblad, B., and Fratiglioni, L., 2005, The age-dependent relation of blood pressure to cognitive function and dementia: Lancet Neurol., v. 4, p. 487-499.

113. Rahman, M., Pressel, S., Davis, B. R., Nwachuku, C., Wright, J. T., Jr., Whelton, P. K., Barzilay, J., Batuman, V., Eckfeldt, J. H., Farber, M. A., Franklin, S., Henriquez, M., Kopyt, N., Louis, G. T., Saklayen, M., Stanford, C., Walworth, C., Ward, H., and Wiegmann, T., 2006, Cardiovascular outcomes in high-risk hypertensive patients stratified by baseline glomerular filtration rate: Ann.Intern.Med., v. 144, p. 172-180.

114. Rashidi, A., Sehgal, A. R., Rahman, M., and O'Connor, A. S., 2008, The case for chronic kidney disease, diabetes mellitus, and myocardial infarction being equivalent risk factors for cardiovascular mortality in patients older than 65 years: Am.J Cardiol., v. 102, p. 1668-1673.

115. Reitan R.M. Validity of the trail making test as an indicator of organic brain damage. [8], 271-276. 1958. Percept Mot Skills.
Ref Type: Serial (Book,Monograph)

116. Reitz, C., Tang, M. X., Manly, J., Mayeux, R., and Luchsinger, J. A., 2007, Hypertension and the risk of mild cognitive impairment: Arch.Neurol., v. 64, p. 1734-1740.

117. Rosamond, W. D., Chang, P., Baggett, C., Bertoni, A., Shahar, E., Deswal, A., Heiss, G., and Chambless, L., 2009, Classification of Heart Failure in the Atherosclerosis Risk in Communities (ARIC) Study: A Comparison With Other Diagnostic Criteria: Circulation, v. 120, p. S506.

118. Rosano, C., Kuller, L. H., Chung, H., Arnold, A. M., Longstreth, W. T., Jr., and Newman, A. B., 2005, Subclinical brain magnetic resonance imaging abnormalities predict physical functional decline in high-functioning older adults: J.Am.Geriatr.Soc., v. 53, p. 649-654.

119. Rosendorff, C., Black, H. R., Cannon, C. P., Gersh, B. J., Gore, J., Izzo, J. L., Kaplan, N. M., O'Connor, C. M., O'Gara, P. T., and Oparil, S., 2007, Treatment of hypertension in the prevention and management of ischemic heart disease - A scientific statement from the American Heart Association council for high blood pressure research and the councils on clinical cardiology and epidemiology and prevention: Hypertension, v. 50, p. E28-E55.

120. Sacco, R. L., Boden-Albala, B., Abel, G., Lin, I. F., Elkind, M., Hauser, W. A., Paik, M. C., and Shea, S., 2001, Race-ethnic disparities in the impact of stroke risk factors: the northern Manhattan stroke study: Stroke., v. 32, p. 1725-1731.

121. Sarnak, M. J., Greene, T., Wang, X., Beck, G., Kusek, J. W., Collins, A. J., and Levey, A. S., 2005, The effect of a lower target blood pressure on the progression of kidney disease: long-term follow-up of the modification of diet in renal disease study: Ann.Intern Med, v. 142, p. 342-351.

122. Sarnak, M. J., Levey, A. S., Schoolwerth, A. C., Coresh, J., Culleton, B., Hamm, L. L., McCullough, P. A., Kasiske, B. L., Kelepouris, E., Klag, M. J., Parfrey, P., Pfeffer, M., Raij, L., Spinosa, D. J., and Wilson, P. W., 2003, Kidney Disease as a Risk Factor for Development of Cardiovascular Disease: A Statement From the American Heart Association Councils on Kidney in Cardiovascular Disease, High Blood Pressure Research, Clinical Cardiology, and Epidemiology and Prevention: Circulation, v. 108, p. 2154-2169.

123. Schaeffner, E. S., Kurth, T., Bowman, T. S., Gelber, R. P., and Gaziano, J. M., 2008, Blood pressure measures and risk of chronic kidney disease in men: Nephrol.Dial.Transplant, v. 23, p. 1246-1251.

124. SHEP, 1991, Prevention of stroke by antihypertensive drug treatment in older persons with isolated systolic hypertension. Final results of the Systolic Hypertension in the Elderly Program (SHEP). SHEP Cooperative Research Group: JAMA, v. 265, p. 3255-3264.

125. Shlipak, M. G., Heidenreich, P. A., Noguchi, H., Chertow, G. M., Browner, W. S., and McClellan, M. B., 2002, Association of renal insufficiency with treatment and outcomes after myocardial infarction in elderly patients: Ann.Intern Med, v. 137, p. 555-562.

126. Shlipak, M. G., Katz, R., Kestenbaum, B., Siscovick, D., Fried, L., Newman, A., Rifkin, D., and Sarnak, M. J., 2009, Rapid decline of kidney function increases cardiovascular risk in the elderly: J Am.Soc.Nephrol., v. 20, p. 2625-2630.

127. Shumaker, S. A., Legault, C., Kuller, L., Rapp, S. R., Thal, L., Lane, D. S., Fillit, H., Stefanick, M. L., Hendrix, S. L., Lewis, C. E., Masaki, K., and Coker, L. H., 2004, Conjugated equine estrogens and incidence of probable dementia and mild cognitive impairment in postmenopausal women: Women's Health Initiative Memory Study: JAMA, v. 291, p. 2947-2958.

128. Sipahi, I., Tuzcu, E. M., Schoenhagen, P., Wolski, K. E., Nicholls, S. J., Balog, C., Crowe, T. D., and Nissen, S. E., 2006, Effects of normal, pre-hypertensive, and hypertensive blood pressure levels on progression of coronary atherosclerosis: J Am.Coll.Cardiol., v. 48, p. 833-838.

129. Skoog, I. and Gustafson, D., 2006, Update on hypertension and Alzheimer's disease: Neurol.Res., v. 28, p. 605-611.

130. Skoog, I. and Gustafson, D., 2003, Hypertension, hypertension-clustering factors and Alzheimer's disease: Neurol.Res., v. 25, p. 675-680.

131. Skoog, I., Lithell, H., Hansson, L., Elmfeldt, D., Hofman, A., Olofsson, B., Trenkwalder, P., and Zanchetti, A., 2005, Effect of baseline cognitive function and antihypertensive treatment on cognitive and cardiovascular outcomes: Study on COgnition and Prognosis in the Elderly (SCOPE): Am.J Hypertens., v. 18, p. 1052-1059.

132. Somes, G. W., Pahor, M., Shorr, R. I., Cushman, W. C., and Applegate, W. B., 1999, The role of diastolic blood pressure when treating isolated systolic hypertension: Archives of Internal Medicine, v. 159, p. 2004-2009.

133. Somes, G. W., Shorr, R. I., and Pahor, M., 1999, A new twist in the J-shape curve?: J Am.Geriatr.Soc., v. 47, p. 1477-1478.

134. Staessen, J. A., Fagard, R., Thijs, L., Celis, H., Arabidze, G. G., Birkenhager, W. H., Bulpitt, C. J., de Leeuw, P. W., Dollery, C. T., Fletcher, A. E., Forette, F., Leonetti, G., Nachev, C., O'Brien, E. T., Rosenfeld, J., Rodicio, J. L., Tuomilehto, J., and Zanchetti, A., 1997, Randomised double-blind comparison of placebo and active treatment for older patients with isolated systolic hypertension. The Systolic Hypertension in Europe (Syst-Eur) Trial Investigators: Lancet, v. 350, p. 757-764.

135. Staessen, J. A., Wang, J. G., and Thijs, L., 2001, Cardiovascular protection and blood pressure reduction: a meta-analysis: Lancet, v. 358, p. 1305-1315.

136. Staessen, J. A., Wang, J. G., Thijs, L., and Fagard, R., 1999, Overview of the outcome trials in older patients with isolated systolic hypertension: J Hum.Hypertens., v. 13, p. 859-863.

137. The ACCORD Study Group, 2010, Effects of Intensive Blood-Pressure Control in Type 2 Diabetes Mellitus: The New England Journal of Medicine, p. NEJMoa1001286.

138. Tzourio, C., Anderson, C., Chapman, N., Woodward, M., Neal, B., Macmahon, S., and Chalmers, J., 2003, Effects of blood pressure lowering with perindopril and indapamide therapy on dementia and cognitive decline in patients with cerebrovascular disease: Arch.Intern Med, v. 163, p. 1069-1075.

139. Vasan, R. S., Larson, M. G., Leip, E. P., Evans, J. C., O'Donnell, C. J., Kannel, W. B., and Levy, D., 2001, Impact of high-normal blood pressure on the risk of cardiovascular disease: N Engl J Med, v. 345, p. 1291-1297.

140. Verdelho, A., Madureira, S., Ferro, J. M., Basile, A. M., Chabriat, H., Erkinjuntti, T., Fazekas, F., Hennerici, M., O'brien, J., Pantoni, L., Salvadori, E., Scheltens, P., Visser, M. C., Wahlund, L. O., Waldemar, G., Wallin, A., and Inzitari, D., 2007, Differential impact of cerebral white matter changes, diabetes, hypertension and stroke on cognitive performance among non-disabled elderly. The LADIS study: J Neurol.Neurosurg.Psychiatry, v. 78, p. 1325-1330.

141. Vermeer, S. E., Prins, N. D., den, H. T., Hofman, A., Koudstaal, P. J., and Breteler, M. M., 2003, Silent brain infarcts and the risk of dementia and cognitive decline: N Engl J Med, v. 348, p. 1215-1222.

142. Wang, Y. and Wang, Q. J., 2004, The prevalence of prehypertension and hypertension among US adults according to the new joint national committee guidelines: new challenges of the old problem: Arch.Intern Med, v. 164, p. 2126-2134.

143. Wechsler D., 1981, Manual for the Wechsler Adult Intelligence Scale: The Psychological Corporation, Harcourt Brace Janvanovich, Inc..

144. Weiner, D. E., Tighiouart, H., Amin, M. G., Stark, P. C., MacLeod, B., Griffith, J. L., Salem, D. N., Levey, A. S., and Sarnak, M. J., 2004, Chronic kidney disease as a risk factor for cardiovascular disease and all-cause mortality: a pooled analysis of community-based studies: J.Am.Soc.Nephrol., v. 15, p. 1307-1315.

145. Weiner, D. E., Tighiouart, H., Levey, A. S., Elsayed, E., Griffith, J. L., Salem, D. N., and Sarnak, M. J., 2007, Lowest systolic blood pressure is associated with stroke in stages 3 to 4 chronic kidney disease: J.Am.Soc.Nephrol., v. 18, p. 960-966.

146. Welsh, K. A., Breitner, J. C. S., and Magruderhabib, K. M., 1993, Detection of Dementia in the Elderly Using Telephone Screening of Cognitive Status:

Neuropsychiatry Neuropsychology and Behavioral Neurology, v. 6, p. 103-110.

147. Whitworth, J. A., 2003, 2003 World Health Organization (WHO)/International Society of Hypertension (ISH) statement on management of hypertension: J Hypertens., v. 21, p. 1983-1992.

148. Winblad, B., Palmer, K., Kivipelto, M., Jelic, V., and Fratiglioni, L., 2004, Introduction: Mild cognitive impairment: beyond controversies, towards a consensus: Journal of Internal Medicine, v. 256, p. 181-182.

149. World Health Organization. World Health Report 2002: Reducing Risk, promoting healthy life. 2002. World Health Organization.
Ref Type: Report

150. Wright, J. T., Jr., Agodoa, L., Contreras, G., Greene, T., Douglas, J. G., Lash, J., Randall, O., Rogers, N., Smith, M. C., and Massry, S., 2002a, Successful blood pressure control in the African American Study of Kidney Disease and Hypertension: Arch.Intern.Med., v. 162, p. 1636-1643.

151. Wright, J. T., Jr., Bakris, G., Greene, T., Agodoa, L. Y., Appel, L. J., Charleston, J., Cheek, D., Douglas-Baltimore, J. G., Gassman, J., Glassock, R., Hebert, L., Jamerson, K., Lewis, J., Phillips, R. A., Toto, R. D., Middleton, J. P., and Rostand, S. G., 2002b, Effect of blood pressure lowering and antihypertensive drug class on progression of hypertensive kidney disease: results from the AASK trial: JAMA, v. 288, p. 2421-2431.

152. Wright, J. T., Dunn, J. K., Cutler, J. A., Davis, B. R., Cushman, W. C., Ford, C. E., Haywood, L. J., Leenen, F. H. H., Margolis, K. L., Papademetriou, V., Probstfield, J. L., Whelton, P. K., and Habib, G. B., 2005, Outcomes in hypertensive black and nonblack patients treated with chlorthalidone, amlodipine, and lisinopril: Jama-Journal of the American Medical Association, v. 293, p. 1595-1607.

153. Wright, J. T., Harris-Haywood, S., Pressel, S., Barzilay, J., Bairnbridge, C., Bareis, C. J., Basile, J. N., Black, H. R., Dart, R., Gupta, A. K., Hamilton, B. P., Einhorn, P. T., Haywood, L. J., Jafri, S. Z. A., Louis, G. T., Whelton, P. K., Scott, C. L., Sinnnons, D. L., Stanford, C., and Davis, B. R., 2008, Clinical outcomes by race in hypertensive patients with and without the metabolic syndrome - Antihypertensive and Lipid-Lowering Treatment to Prevent Heart Attack Trial (ALLHAT): Archives of Internal Medicine, v. 168, p. 207-217.

154. Yusuf, S., Sleight, P., Pogue, J., Bosch, J., Davies, R., and Dagenais, G., 2000, Effects of an angiotensin-converting-enzyme inhibitor, ramipril, on cardiovascular events in high-risk patients. The Heart Outcomes Prevention Evaluation Study Investigators [published errata appear in N Engl J Med 2000 Mar 9;342(10):748 and 2000 May 4;342(18):1376]: N Engl J Med, v. 342, p. 145-153.

APPENDIX 1: Abbreviations Used

AAA:	Abdominal Aortic Aneurysm
AASK:	African American Study of Kidney Disease and Hypertension
ABI:	Ankle Brachial Index
ACC:	American College of Cardiology
ACCORD:	Action to Control Cardiovascular Risk in Diabetes
ACE:	Angiotensin Converting Enzyme
ACR:	Albumin to Creatinine Ratio
ACS:	Acute Coronary Syndrome
AD:	Alzheimer's Disease
AE:	Adverse Event
AHA:	American Heart Association
ALLHAT:	Antihypertensive and Lipid-Lowering Treatment to Prevent Heart Attack Trial
ARB:	Angiotensis Receptor Blocker
ARIC:	Atherosclerosis Risk in Communities
AS:	Ancillary Science
ASCOT:	Anglo-Scandinavian Cardiac Outcomes Trial
BID:	Twice Daily
BNT:	Boston Naming Test
BP:	Blood Pressure
BPH:	Benign Prostatic Hyperplasia
CABG:	Coronary Artery Bypass Grafting
CAD:	Coronary Artery Disease
CAMELOT:	Comparision of Amlodipine vs Enalapril to Limit Occurrences of Thrombosis Trial
CC:	Coordinating Center
CCB:	Calcium Channel Blockers
CCN:	Clinical Center Network
CE:	Carotid Endarterectomy
CEA:	Cost-Effectiveness Analysis
CHD:	Coronary Heart Disease
CHF:	Chronic Heart Failure
CHS:	Cardiovascular Health Study
CKD:	Chronic Kidney Disease
Co-PI:	Co-Principal Investigator
CPT:	Current Procedural Terminology
CUA:	Cost-Utility Analysis
CV:	Cardiovascular
CVD:	Cardiovascular Disease
DASH:	Dietary Approaches to Stop Hypertension
DBP:	Diastolic Blood Pressure
DDC:	Drug Distribution Center
DHP:	Dihydropyridine
DQ:	Dementia Questionnaire
DSC:	Digit Symbol Coding test
DSMB:	Data Safety Monitoring Board
DSM-IV:	Diagnostic and Statistical Manual of Mental Disorders - Fourth Edition
DSST:	Digit Symbol Substitution Test
DST:	Digit Span Test
ECG:	Electrocardiogram
ED:	Erectile Dysfunction
eGFR:	Estimated Glomerular Filtration Rate
EnaC Inhibitor:	Epithelial Sodium Channel Inhibitor
EPICARE:	Epidemiological Cardiology Research Center
EQ-5D:	EuroQol 5 Dimensional Descriptive System
ESRD:	End Stage Renal Disease
EUROPA:	European Trial on Reduction of Cardiac Events with Perindopril in Stable Coronary Artery Disease
FAQ:	Functional Activities Questionnaire
FDA:	Food and Drug Administration
FES-I:	Falls Self-Efficacy Scale International
FRS:	Framingham Risk Score
FSFI:	Female Sexual Function Assessment
GCP:	Good Clinical Practice
GEMS:	Gingko Evaluation of Memory Study
GFR:	Glomerular Filtration Rate
GXT:	Graded Exercise Test
HDFP:	Hypertension Detection and Follow-up Program
HF:	Heart Failure
HIPAA:	Health Information Portability and Accountability Act
HOPE:	Hospital Outcomes Project for the Elderly

HOT:	Hypertension Optimal Treatment trial
HRQL:	Health Related Quality of Life
HTN:	Hypertension
HVLT:	Hopkins Verbal Learning Test
HYVET:	Hypertension in the Very Elderly Trial
HYVET COG:	Hypertension in the Very Elderly Trial – cognitive function assessment
ICER:	Incremental Cost-Effectiveness Ratio
ID:	Identification
IIEF:	International Index of Erectile Function
IRB:	Institutional Review Board
ISH:	Isolated Systolic Hypertension
JNC:	Joint National Committee
JNC-7:	The Seventh Report of the Joint National Committee on Prevention, Detection, Evaluation, and Treatment of High Blood Pressure
LMT:	Logical Memory Test
LVH:	Left Ventricular Hypertrophy
MAP:	Mean Arterial Pressure
MAR:	Missing-at-Random Analyses
MCI:	Mild Cognitive Impairment
MDRD:	Modification of Diet in Renal Disease Study
MI:	Myocardial Infarction
MIND:	Memory and Cognition In Decreased Hypertension
MoCA:	Montreal Cognitive Assessment
MOP:	Manual of Procedures
MPQC:	Measurement Procedures and Quality Control
mRey-O:	Modified Rey-Osterrieth Complex Figure
MRI:	Magnetic Resonance Imaging
NEJM:	New England Journal of Medicine
NKF:	National Kidney Foundation
NHANES:	National Health and Nutrition Examination Survey
NHLBI:	National Heart, Lung, and Blood Institute
NIA:	National Institute on Aging
NIDDK:	National Institute of Diabetes and Digestive and Kidney Diseases
NIH:	National Institutes of Health
NINDS:	National Institute of Neurological Disorders and Stroke
OH:	Orthostatic Hypotension
P&P:	Publications and Presentations
PAD:	Peripheral Artery Disease
PCI:	Percutaneous Coronary Intervention
PEACE:	Prevention of Events with Angiotensin Coverting Enzyme
PHI:	Private Health Information
PHQ:	Patient Health Questionnaire
PI:	Principal Investigator
PKD:	Polycystic Kidney Disease
PROGRESS:	Perindopril Protection Against Recurrent Stroke Study
PTS:	Participant Tracking System
QALY:	Quality Adjusted Life Years
QC:	Quality Control
RAAS:	Renin-angiotensin-aldosteribe system
RAS:	Renin Angiotensin System
SAE:	Serious Adverse Event
SBP:	Systolic Blood Pressure
SCOPE:	Study on Cognition and Prognosis in the Elderly
SHEP:	Systolic Hypertension in the Elderly Program
SPRINT:	Systolic Blood Pressure Intervention Trial
SPRINT MIND:	SPRINT Memory and Cognition In Decreased Hypertension
SSL:	Secure Socket Layer
SVID:	Small Vessel Ischemic Disease
Syst-Eur:	Systolic Hypertension in Europe Trial
TICS-M:	Modified Telephone Interview for Cognitive Status
TMT:	Trail Making Test
UKPDS:	United Kingdom Prospective Diabetes Study
WHI:	Women’s Health Initiative
WHIMS:	Women’s Health Initiative Memory Study
WWW:	World Wide Web

APPENDIX 2: Computational Details and Sensitivity Analyses for the CVD outcome

Power computations were developed using event rates observed in ALLHAT. The ALLHAT Coordinating Center provided us with summary data across all three arms allowing us to calculate event rates using different combinations of baseline characteristics. Event rates were calculated using a composite outcome including fatal CVD, non-MI acute coronary syndrome, and nonfatal MI, stroke, and heart failure. For ALLHAT participants without diabetes, the annual event rate was 4.39 %/yr. (Note: ALLHAT used hospitalized angina rather than non-MI acute coronary syndrome.)

This rate of 4.39 %/yr provides a starting point for the estimation of event rates we will expect in SPRINT. Several factors can be considered which suggest that these rates should be either increased or decreased. Factors arguing for an increased event rate include (1) SPRINT will have an older cohort of participants than did ALLHAT, (2) SPRINT will use the Framingham risk score of ≥15% 10-year CVD risk as an inclusion criterion, and (3) inclusion of a substantial group of participants with Stage 3 or Stage 4 CKD. Factors that are expected to reduce the event rate include (1) the temporal trend towards a reduction in CVD event rates in the U.S. and (2) a more rigorous definition of non-MI acute coronary syndrome that will be used in SPRINT. It is difficult to precisely estimate the impact that these five factors will have on the SPRINT event rate.

In ALLHAT, event rates increased substantially with age. The event rate for participants 70 to <75 years old was 5.19 %/yr; for participants ≥75 years old, the event rate was 6.99 %/yr. In ALLHAT 17.7% of the participants were 70 to <75 years old, while 18.5% were ≥75 years old. We expect that participants in these age categories will represent a greater fraction of the SPRINT cohort. Approximately 50% (4625 participants) are expected to be at least 70 years old, while 35.1% (3250 participants) are expected to be ≥75 years old. This will likely yield a higher event rate in SPRINT, compared to ALLHAT.

The event rate in ALLHAT among participants with 10-year Framingham risk ≥15% at baseline was 4.67 %/yr. Our including people with ≥15% 10-year risk will help to ensure a higher event rate.

We expect that 4300 SPRINT participants will have eGFR 20 to <60 mL/min/1.73m^2 with equal numbers above and below 45 mL/min/1.73m^2. In ALLHAT, the event rate was 5.89 %/yr for those with eGFR 45 to <60 mL/min/1.73m^2. Among those <45, the event rate was 8.24 %/yr. In ALLHAT, 18.6% had eGFR <60 mL/min/1.73m^2 as compared with the expected 46.7% in SPRINT. Increasing the numbers of participants with CKD in SPRINT will help increase the event rates.

We compared ALLHAT participants with diabetes to participants in the ACCORD BP trial (all of whom have diabetes) using outcome variables that are as similar as possible. In ALLHAT the event rate was 5.90 %/yr. The corresponding event rate in ACCORD was 3.43 %/yr. The reduction in event rates between ALLHAT and ACCORD could be due to a temporal trend (ALLHAT was 1994—1999, ACCORD was 2001—2009), because ALLHAT participants were older (mean 67 years) than ACCORD (mean 62.2 years), or for other reasons.

Exactly how we should use the ALLHAT data to estimate the event rates for SPRINT is unclear. Since the rates in ACCORD were approximately half of those in ALLHAT, *for the purposes of power we will assume that the SPRINT rates will also be half of the ALLHAT rates.* This assumption balances the possibility of a further temporal trend in event rate reduction with the fact that participants recruited for SPRINT will be older, have lower kidney function, and have greater Framingham CVD risk scores than those recruited in either ALLHAT or ACCORD. We expect that this may be slightly conservative. Thus, we assume that the event rate in SPRINT will be approximately 2.2 %/yr for the composite outcome including non-fatal MI, non-fatal stroke, cardiovascular death, hospitalized heart failure, and non-MI acute coronary syndrome.

We have assumed a 2-year uniform accrual period, 3 years 10 months minimum follow-up (assumes that closeout visits occur uniformly over a 4-month period), and a 2 sided significance level of 0.05. The effect size for the primary outcome is assumed to be 20% in the entire sample and the CKD subsample, and 25% in the Senior subsample. Loss to follow-up and events are assumed to follow an exponential model. We expect that the annual rate of loss to follow-up will be approximately 2% but have included rates up to 3% to be conservative. Calculations made using two methods (Lachin and Foulkes, 1986;Lakatos, 1988) were similar. Power for the primary outcome for a range of event rates and annual loss rates is presented in Table 1 for the assumed effect size of 20%.

Table 1. Power for the primary outcome in entire sample of 9250 participants for a 20% effect (Hazard Ratio of 0.8).					
Annual Loss Rate (%/yr)	Annual Standard Arm Event Rate (%/yr)				
	1.8	2.0	2.2	2.4	2.6
1	82.9	86.5	89.4	91.7	93.5
2	82.0	85.7	88.7	91.0	93.0
3	81.1	84.8	87.9	90.4	92.4

In ALLHAT the event rates were 5.89 %/yr and 8.24 %/yr for people whose eGFR was 45 to <60 or <45 mL/min/1.73m^2. We will assume that the event rate for the primary outcome in SPRINT will be 4 %/yr among participants with eGFR <60 mL/min/1.73m^2. Power for the primary outcome among SPRINT participants with CKD for a range of event rates and annual loss rates is presented in Table 2 for the assumed effect size of 20%.

Table 2. Power for the primary outcome in CKD subsample (eGFR < 60 mL/min/1.73m^2) of 4300 participants for a 20% effect (Hazard Ratio of 0.8).					
Annual Loss Rate (%/yr)	Annual Standard Arm Event Rate (%/yr)				
	3.5	3.75	4.0	4.25	4.5
1	77.9	80.5	82.7	84.8	86.6
2	76.9	79.5	81.9	83.9	85.8
3	75.9	78.6	80.9	83.1	85.0

In ALLHAT, the event rate was 6.99 %/yr among participants at least 75 years old. Applying the same halving as was done above for the entire sample, we will assume that the event rate in

SPRINT will be 3.5 %/year among participants ≥75 years old. Power for the primary outcome among SPRINT Senior for a range of event rates and annual loss rates is presented in Table 3 for the assumed effect size of 25%.

Table 3. Power for the primary outcome in Senior subsample (≥75 years old) of 3250 participants for a 25% effect (Hazard Ratio of 0.75).					
Annual Loss Rate (%/yr)	Annual Standard Arm Event Rate (%/yr)				
	3.0	3.25	3.5	3.75	4.0
1	79.9	82.8	85.3	87.5	89.4
2	79.0	81.9	84.5	86.7	88.6
3	78.0	81.0	83.6	85.9	87.9

APPENDIX 3: Computational Details and Sensitivity Analyses for the MIND outcomes

Dementia. The primary outcome for SPRINT MIND is all-cause dementia. Table 1 summarizes dementia rates from HYVET-COG (Peters, 2008), the Ginkgo Evaluation of Memory Study (GEMS) (DeKosky, 2008), the Cardiovascular Health Study (CHS) (Fitzpatrick, 2004) and the Women's Health Initiative Memory Study (WHIMS) (Shumaker, 2004). In HYVET-COG, there was a 14% non-significant decline in dementia. Overall annual dementia rate varied from 0.13% to 3.86%. The Women's Health Initiative Memory Study (WHIMS) (Shumaker, 2004) recruited women 65 and older with a mean age of 69 in two hormone replacement therapy interventions. Both trials were stopped early because of unexpected increased health risks in women receiving the hormone therapy. Of the studies reported here, WHIMS may be the least similar to SPRINT.

Table 1. Annual rates of dementia from previous studies.

Age	eGFR	HYVET-COG	GEMS	CHS	WHIMS
<75				1.29	0.08
75+			3.09 (3.86)[1]	4.55	0.81
	<45		4.87 (6.39)		
	45-59.9		3.02 (3.20)		
	60-89.9		2.87 (3.70)		
	90+		3.86 (4.51)		
80+		3.50			
ALL		3.50	3.09 (3.86)	2.62	0.13

[1] With prior CVD

Based on these data and the expected number of SPRINT participants 75 or older, and with CKD or MCI at baseline, we expect the annual event rate in SPRINT to be 3.1%-3.5%. In meta-analyses performed by the HYVET investigators, three of the four trials had hazard ratios ranging from 0.84 to 0.90. A reasonable goal for SPRINT MIND is to detect a relative difference between arms expressed by a hazard ratio of 0.5 to 0.8 for dementia. Using a 2-sided proportional hazards regression test of time until first incidence of dementia, we can expect at least 79% power for annual dementia rates of 3.1%-3.5% and an effect size of 0.15 and 96% power for annual dementia rates of 3.2%-3.5% and an effect size of 0.20.

Cognitive Function. SPRINT will include 2,800 participants receiving the extended cognitive battery at baseline, and years 2 and 4 post randomization. We obtained the standard deviations for several of the tests included in the SPRINT battery to determine detectable differences. The standard deviation for the Digit Symbol Substitution Test is from actual ACCORD MIND data 40 months post randomization adjusted for baseline and stratifying factors. Actual means were not available so we used the ACCORD MIND assumptions in their sample size calculations based on CHS data. GEMS provided us with standard deviations and means for Trails A & B, Digit

Span and the Boston Naming Test. Table 2 shows that we can detect mean differences for each test of 5.1% or less between the two SPRINT treatment groups at year 4, with 90% statistical power, assuming 3%/year loss to follow-up. The statistical power will even be increased when combining the scores for these tests in each domain.

Table 2. Means, standard deviations and power for cognitive tests.

**Cognitive Test**	**Mean (STD)**	**Power**	
		80%	90%
Effect Size		0.114	0.132
Digit Symbol Substitution Test	39.5[1] (7.9)[2]	0.90 (2.4%)	1.05 (2.7%)
Trails A[3]	47.5 (18.1)	2.07 (4.4%)	2.40 (5.1%)
Trails B[3]	124.4 (40.6)	4.65 (3.7%)	5.38 (4.3%)
Digit Span[3]	13.9 (2.6)	0.30 (2.2%)	0.34 (2.4%)
Boston Naming Test[3]	26.2 (2.6)	0.30 (1.1%)	0.34 (1.3%)

[1] From ACCORD MIND assumptions in sample size calculations based on CHS data
[2] From actual ACCORD MIND data at 40 months post randomization
[3] From GEMS at 48 months post randomization

MRI. We will perform MRI in 640 of SPRINT MIND participants. The standard deviations for total abnormal tissue volume and total brain volume from the ACCORDMIND study 40 months post randomization adjusted for baseline and cranial size are 2.77 cm^3 and 16.45 cm^3. The final analysis of the MRI data collected in SPRINT MIND will compare the mean total abnormal tissue and mean total brain volumes between the groups, controlling for the baseline MRI value and cranial side. With 640 participants (320 participants in each treatment group), after accounting for a 3%/yr loss to-follow-up, and assuming a 0.05 two-sided significance level, we will be able to detect group differences in total abnormal vascular lesion volumes of 0.65 cm^3 and 0.76 cm^3, and in total brain volumes of 3.9 cm^3 and 4.5 cm^3 over 4 years, with 80% and 90% power, respectively.
